# Functional Group Properties and Position Drive Differences in Xenobiotic Plant Uptake Rates, but Metabolism Shares a Similar Pathway

**DOI:** 10.1021/acs.estlett.3c00282

**Published:** 2023-06-21

**Authors:** Claire P. Muerdter, Megan M. Powers, Danielle T. Webb, Sraboni Chowdhury, Kaitlyn E. Roach, Gregory H. LeFevre

**Affiliations:** † Department of Civil and Environmental Engineering, The University of Iowa, 4105 Seamans Center, Iowa City, Iowa 52242, United States; ‡ IIHR-Hydroscience and Engineering, The University of Iowa, 100 C. Maxwell Stanley Hydraulics Laboratory, Iowa City, Iowa 52242, United States; § University of Iowa Secondary Student Training Program, Belin-Blank Center, 600 Blank Honors Center, Iowa City, Iowa 52242, United States

**Keywords:** phytotransformation, phytoremediation, molecular descriptors, metabolites, benzimidazoles, benzotriazoles, glutathione, modeling

## Abstract

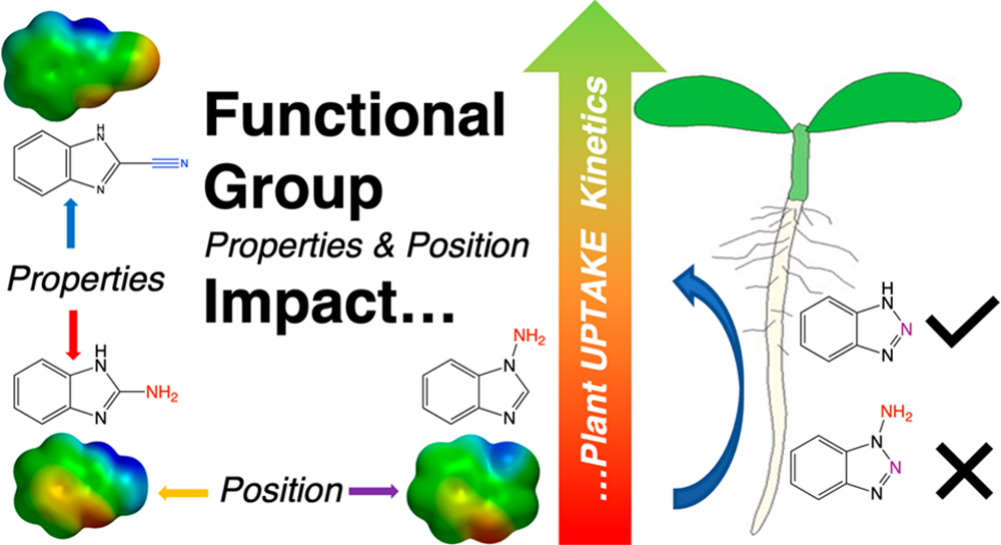

Plant uptake of xenobiotic compounds is crucial for phytoremediation (including green stormwater infrastructure) and exposure potential during crop irrigation with recycled water. Experimentally determining the plant uptake for every relevant chemical is impractical; therefore, illuminating the role of specific functional groups on the uptake of trace organic contaminants is needed to enhance predictive power. We used benzimidazole derivatives to probe the impact of functional group electrostatic properties and position on plant uptake and metabolism using the hydroponic model plant *Arabidopsis thaliana*. The greatest plant uptake rates occurred with an electron-withdrawing functional group at the 2 position; however, uptake was still observed with an electron-donating group. An electron-donating group at the 1 position significantly slowed uptake for both benzimidazole- and benzotriazole-based molecules used in this study, indicating possible steric effects. For unsubstituted benzimidazole and benzotriazole structures, the additional heterocyclic nitrogen in benzotriazole increased plant uptake rates compared to benzimidazole. Analysis of quantitative structure–activity relationship parameters for the studied compounds implicates energy-related molecular descriptors as uptake drivers. Despite significantly varied uptake rates, compounds with different functional groups yielded shared metabolites, including an impact on endogenous glutathione production. Although the topic is complex and influenced by multiple factors in the field, this study provides insights into the impact of functional groups on plant uptake, with implications for environmental fate and consumer exposure.

## Introduction

Plant uptake can be a critical factor in the environmental fate of trace organic contaminants (TOrCs).^1,2^ For example, plants can take up and metabolize TOrCs in green stormwater infrastructure^3^ and treatment wetlands,^4^ or crops may take up TOrCs upon application to fields (e.g., pesticides) or residual TOrCs are present during irrigation with recycled water.^5−7^ Plants may take up and transform the chemicals into less toxic compounds (phytoremediation),^8^ or TOrCs may present a possible exposure risk to consumers if they accumulate in edible plant tissue.^5^ Prediction of these behaviors is complicated because the propensity and extent of plant uptake and metabolism of compounds vary with both chemical characteristics^9−12^ and plant properties.^13^

Distinct pathways exist for the uptake of organic compounds into plants. Chemicals may be transported passively across root membranes with the transpiration stream.^14−16^ Alternatively, chemicals may be actively taken up into the plant by a transporter protein.^16,17^ Transporter protein-mediated processes yield chemical uptake rates greater than that of the passive transpiration stream (i.e., “active uptake”)^18^ but are poorly understood. Plants have specialized transporters for amino acid uptake^19^ that facilitate rapid, selective uptake into the plant.^20^ Amino acid transporters are believed to be sufficiently nonspecific to permit transport of other organic, nitrogenous TOrCs.^18,21^ For example, the levels of multiple genes for amino acid transporters increased in rice exposed to the pesticide isoproturon,^22^ and organic cation transporters are thought to mediate plant uptake of the antidiabetic drug metformin.^23^ Transporter-facilitated active uptake could be a critical fate mechanism in some natural treatment systems in which plant–TOrC interaction time may be short (e.g., stormwater infiltration^24,25^) and may be important for direct plant conjugation of TOrCs (i.e., rapid formation of phase II metabolites^18,26^).

Existing plant uptake models generally focus on transpiration-based passive uptake relevant to nonpolar, nonionizable compounds and exclude transporter-mediated uptake^14^ that may be relevant to many TOrCs.^27,28^ Previous uptake work (e.g., refs (10), (11), and (29−31)) has focused on whole-compound characteristics (e.g., log *K*_OW_, molecular mass, and number of rotatable bonds) to predict plant translocation or bioaccumulation, which are important chemical properties but do not always adequately predict plant uptake,^28^ particularly for active uptake mechanisms. With transporter-mediated plant uptake, the fit of the substrate into the transporter protein can be driven by the compound functional group;^32,33^ thus, probing the influence of functional groups (e.g., sizes, position, and electrostatic character) on plant uptake presents a critical need. Functional group polarity and/or partial charge distribution can also be important for toxicological impacts of xenobiotics.^34^ The vast number of anthropogenic chemicals in the environment makes individual testing of TOrCs impractical and instead demands predictive capabilities based on chemical structure.^5^ Therefore, the objective of this work was to systematically test a suite of compounds with the same chemical base structure and slightly varied functional group properties to evaluate differences in plant uptake kinetics (note that the “base” structure herein indicates the shared molecule structure without added functional groups, not referencing acid/base). We hypothesized that slight changes in chemical structure (i.e., functional group position on the base molecule and its electrostatic properties) would yield significant measurable changes in bulk plant uptake rates, likely driven by active uptake. We discovered distinct differences in uptake kinetics based on the properties (electron-withdrawing vs -donating) and position of functional groups. Additionally, we report multiple novel plant metabolites and a shared impact on endogenous plant glutathione production, despite the wide variety of functional group differences featured in parent compounds and uptake kinetics. The outcomes of this work uncover a deeper understanding of plant uptake of TOrCs and have urgent implications for water recycling for irrigation with concomitant exposure to humans and livestock.

## Materials and Methods

### Chemicals

We employed benzimidazole as the model base structure (see note above) due to the wide variety of commercially available benzimidazole compounds with slight differences in chemical structure [i.e., different functional groups and positions; eight benzimidazole derivatives (Table S1)]. We also compared the plant uptake kinetics of benzimidazoles with benzotriazoles. Both benzimidazoles and benzotriazoles are taken up by plants,^18,35^ with the rate of benzotriazole uptake known to exceed the transpiration rate^18^ (i.e., active uptake; the rate of uptake of benzotriazole by hydroponic plants was 6–14-fold greater than that of passive evapotranspiration), and the base structures vary by only a single nitrogen in the heterocyclic ring. Benzimidazole derivatives are commonly used fungicides,^36−38^ and benzotriazole is a widely used corrosion inhibitor.^39^ Both have high solubilities and are present in environmental waters.^40^ Specific chemicals used in the experiments are fully described in the Supporting Information.

### Experimental Design

#### Plant Exposure Experiments

*Arabidopsis* seeds were surface-sterilized using a previously published bleach procedure,^18,41−43^ with minor modifications (detailed in the Supporting Information). Seeds were then grown aseptically in hydroponic medium in washed/autoclaved Magenta GA-7-3 boxes (Bioworld), also using a previously published procedure, with minor modifications. The exposure experiments were modeled on previous work^18,42,44^ and are described in detail in the Supporting Information (Figure S1). Briefly, after an 11–13 day period of *Arabidopsis* growth in unspiked sterile hydroponic medium, the medium was exchanged for sterile medium spiked with a single compound at a concentration of 20 μg/L [i.e., benzimidazole, benzotriazole, or a derivative (Table S1)] under an exposure treatment condition. A no-plant abiotic control was also created from the same medium master mix to quantify non-plant-related losses (e.g., photolysis, hydrolysis). Each treatment and control consisted of three or four experiments. Sampling of the hydroponic medium occurred throughout the duration of the 48 h exposure period [with limited 10 day exposure sampling (Figure S3)] to quantify depletion of TOrCs from the hydroponic medium (details in the Supporting Information). Quantifying TOrC depletion in the hydroponic solution was employed in the experiments because any subsequent in-plant transformation would first require uptake into plants, and transporter-facilitated uptake is unlikely to be influenced by *in planta* TOrC concentration gradients like passive processes; sorption was quantified separately (see below). Second-order rates were calculated from the kinetic data (details in the Supporting Information); second-order kinetics have been previously reported for similar results of hydroponic plant uptake kinetics.^26,42,44^ Except during active sampling, boxes were maintained in a Percival growth chamber alternating between light for 16 h at 23 °C and dark for 8 h at 21 °C. Sorption of each compound to plant tissue was quantified individually using 11- to 12-day-old *Arabidopsis* plants unexposed to chemicals grown in parallel with the experimental treatments (i.e., same biomass); sorption samples were taken 5 min after plants and chemicals were combined to isolate immediate sorption to plant tissue (details in the Supporting Information). Paired *t* tests determined significant differences between the removal rates of compounds. Departure from the linear null slope at the 95% confidence interval determined whether a significant change in compound concentration occurred over time. Benzimidazole concentrations were quantified using LC-MS/MS (detailed in the Supporting Information).

#### Plant Metabolomics

We probed the effects of specific functional groups on the plant metabolism of three representative benzimidazoles with the goal of discovering shared and/or unique metabolites when the TOrC functional group varied substantially: benzimidazole (BZ), 2-cyanobenzimidazole (CN-BZ), and carbendazim (Carb-BZ). To ensure a sufficiently high concentration of metabolites for detection, a nominal concentration of 400 μg/L for the compound of interest (BZ, CN-BZ, or Carb-BZ) was used in each treatment group of *Arabidopsis* plants grown in parallel with an unspiked control group. Each group had seven biological replicate sample boxes except Carb-BZ (*n* = 3). Plant tissue was harvested at 24 h following chemical exposure (at a point of rapid yet substantial plant metabolism, consistent with prior work^18,44^) by separating tissue from the liquid medium and then frozen at −20 °C until plant tissue was extracted. Freeze-drying and plant tissue extraction followed a previously published procedure,^18,44^ detailed in the Supporting Information. Extracted plant tissues were analyzed on a Thermo Q-Exactive Orbitrap high-resolution mass spectrometer, run in MS and both positive and negative data-dependent MS/MS modes, and metabolomics data were analyzed via Compound Discoverer version 3.1 (described in full in the Supporting Information). Compounds with a treatment versus control *p* value of ≤0.05 and a peak area fold change ratio of ≥100 were selected for further analysis. The compound was listed as a proposed structure if the accurate mass deviation between the proposed compound and measured *m*/*z* was <10 ppm.

## Results and Discussion

### Functional Group Properties and Position Drive Differences in Plant Uptake Kinetics

**Figure 1:**
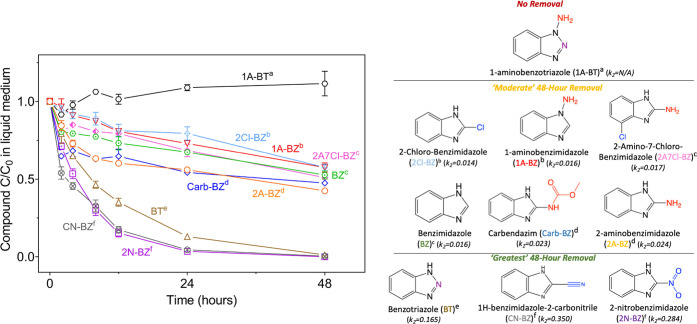
Removal of each compound, each tested separately with *Arabidopsis*, from the hydroponic plant growth medium. Abiotic controls (Figures S2–S5) and sorption tests (Table S4) demonstrate that plant uptake is responsible for the majority of removal for all compounds. The compounds formed three distinct groups: no removal, moderate 48 h removal (*C*/*C*_0_ = 43–58%), and greatest 48 h removal (*C*/*C*_0_ = 0–1%). Superscript letters a–f are used to indicate significant differences (*p* < 0.05) between the compounds with respect to removal rates. Full *p* values for comparisons of plant uptake rates for different pairs of compounds are listed in Table S6. Three significantly different groups occurred among the moderate removal compounds: (b) 2Cl-BZ and 1A-BZ, (c) 2A7Cl-BZ and BZ, and (d) Carb-BZ and 2A-BZ. Two significantly different groups occurred among the greatest removal compounds: (e) BT and (f) CN-BZ and 2N-BZ. Functional groups are colored blue for electron-withdrawing and red for electron-donating. The heterocyclic ring nitrogen found in the two benzotriazole compounds and not in the benzimidazole compounds is colored purple. The second-order rate constant (*k*_2_) value for depletion kinetics is provided; note values here are listed as unitless on the basis of the relative initial concentration (full details and data in the Supporting Information). Error bars represent the standard error (*n* = 2–4 for *t* = 0 data points, and *n* = 4 for all other data points), with nonvisible error bars obscured by the symbols.

We discovered that plant uptake kinetics of benzimidazole compounds was driven by functional group position and electrostatic properties, with kinetics consistent with active uptake for multiple compounds. TBZ removal was primarily abiotic (Figure S3) and therefore is not included further in our analysis. For the other measured compounds, three groupings emerge on the basis of the rapid uptake (48 h) *C*/*C*_0_ values (Figure 1). We focused on the 48 h period because the contact time between plants and water is often limited (e.g., irrigation, bioinfiltration). 1A-BT was not removed from the medium (“no removal”). In the second group, 2Cl-BZ, 1A-BZ, 2A7Cl-BZ, BZ, Carb-BZ, and 2A-BZ all exhibited “moderate” removal (*C*/*C*_0_ = 43–58%) at 48 h. In the third grouping, BT, CN-BZ, and 2N-BZ exhibited complete or nearly complete removal (*C*/*C*_0_ = 0–1%; “greatest removal”) at 48 h. For a limited number of compounds, we collected data 10 days after exposure to the chemicals (Figure S3). No-plant abiotic controls (Figures S2–S5) for all remaining compounds demonstrated no significant removal of >3% (Table S5 for details), indicating that plant processes (plant uptake or sorption to plant tissue) were the dominant removal mechanisms. Sorption to plant tissue ranged from 0% to 12% for all compounds (except CN-BZ at 33%) based on sorption tests for individual compounds (Table S4), indicating that plant uptake was indeed responsible for the majority of removal.

The position and electrostatic nature of functional groups on the base molecule structure significantly impacted plant uptake kinetics in this study. For molecules with the benzimidazole base, a strongly electron-withdrawing group at the 2 position (i.e., cyano in CN-BZ or nitro in 2N-BZ, 2.77 and 3.30 Pauling units, respectively^45,46^) exhibited the most rapid uptake (*p* = 0.003 and 0.008, respectively, for each compound vs BZ). Even moderate to strong electron-donating groups at the 2 position (i.e., 2A-BZ and Carb-BZ; the 2A-BZ amino group electronegativity = 2.42 Pauling units^46^) resulted in significantly more rapid plant uptake than BZ alone (*p* = 0.003 and 0.004, respectively). The weakly electron-withdrawing chlorine^45^ at the 2 position (i.e., 2Cl-BZ) significantly decreased plant uptake kinetics compared to those of other compounds containing only a 2 position functional group [either strongly electron-withdrawing groups in CN-BZ (*p* = 0.002) and 2N-BZ (*p* = 0.004), or electron-donating groups in Carb-BZ (*p* = 0.003) and 2A-BZ (*p* = 0.003)]. Addition of the chlorine to the aromatic ring decreased the uptake rate (i.e., 2A7Cl-BZ vs 2A-BZ; *p* = 0.03), similar to our previous observations^18^ in which addition of a methyl group to the BT aromatic ring decreased plant uptake kinetics.

In contrast to the 2 position, the addition of a strongly activating electron-donating amino group at the 1 position on the BZ base structure (1A-BZ) resulted in a significantly decreased rate of plant uptake compared to that of BZ (*p* = 0.006). The same trend occurred for 1A-BT compared to BT (*p* = 0.01). Although both base structures demonstrated decreased rates of uptake with the addition of the 1-amino group, the effect was especially pronounced on the benzotriazole base structure, with nearly complete removal at 48 h for BT compared to no significant removal for 1A-BT. This difference is likely due to the availability of a reactive bonding site on the 2 position carbon in 1A-BZ, allowing for some transporter interaction even if the 1 position reactive heterocyclic ring nitrogen is occupied by an amino group.^47,48^ In contrast, in 1A-BT no such bonding site is available in the triazole ring (even accounting for 1H/2H resonance^49^) when an amino group is present.

Upon comparison of the base structures alone, BT exhibits a rate of plant uptake significantly higher than that of BZ (*p* = 0.02). This may result from the ability of BT to form 1H and 2H tautomers^49^ due to resonance, enabling formation of an N:H bonding site at the 1 or 2 position in the heterocyclic triazole ring. The 2 position in benzimidazoles is known to be important for interactions with other compounds; for example, benzimidazole functional groups at the 2 position promote the binding of benzimidazole to metal surfaces.^50^ In this work, we observed that functional groups attached at position 2 increased plant uptake rates (e.g., 2A-BZ, Carb-BZ, CN-BZ, 2N-BZ). The observed uptake rates may be affected by substrate steric effects with the putative plant transporter protein, such that a functional group at position 2 results in more effective protein binding. Steric effects with transporter proteins have been reported previously in plants.^32,33,47^ Further knowledge related to the putative plant transporter protein structure and binding properties is required for additional fundamental predictions.

These findings are consistent with work demonstrating the importance of polarity and electrostatic interactions in plant transporter uptake of amino acids and sugars.^47,48^ In this study, the plant uptake rate correlated positively with the overall dipole moment of the molecule [*R*^2^ = 0.73 for molecules that could be calculated (Supporting Information)]; however, other uninvestigated electrostatic interactions and/or molecular descriptors cannot be excluded. Indeed, our analysis of quantitative structure–activity relationship (QSAR) parameters for the studied compounds (Supporting Information) implicates energy-related molecular descriptors as drivers of uptake (Tables S7–S9 and Figure S7). For example, the uptake rate constant was negatively correlated with the energy of the lowest unoccupied molecular orbital (LUMO; ρ = −0.81, and *p* = 0.004) and positively correlated with the minimum local ionization potential at the electron density surface (ρ = 0.76, and *p* = 0.012), both energy-related molecular descriptors. Although sample size limits differentiation between “moderate” (*n* = 6) and “greatest” (*n* = 3) removal groups and cannot provide insights into specific mechanisms, the molecular descriptors are consistent with the aforementioned literature describing energy-related factors in transporter-mediated plant uptake.

BT can be taken up into hydroponic *Arabidopsis* at a rate exceeding transpiration and is thought to be the result of active transporter uptake,^18^ which may likewise apply to the other compounds in the “greatest removal” category. Existing plant uptake models for xenobiotics often focus on transpiration-based passive uptake assumptions rather than transporter-mediated processes. For example, a recent machine learning model of root concentration factor of organic compounds^12^ did not include polar/ionic compounds. When a neural network plant uptake model^9^ was specifically applied for the compounds used in this study (see the Supporting Information for method details), the model predicted high uptake rates for both BZ and Carb-BZ but slightly higher uptake rates for BZ (TSCF of 0.62) versus Carb-BZ (TSCF of 0.52). In contrast, we observed the reverse: slightly greater Carb-BZ uptake than BZ uptake (*p* = 0.004). Both compounds were indeed in the “moderate” removal group, however, as predicted by the model. Future plant uptake models could include more polar compounds and account for possible transporter-driven uptake.

### Benzimidazoles with Varied Functional Groups Result in Shared and Unique Plant Metabolites

**Figure 2:**
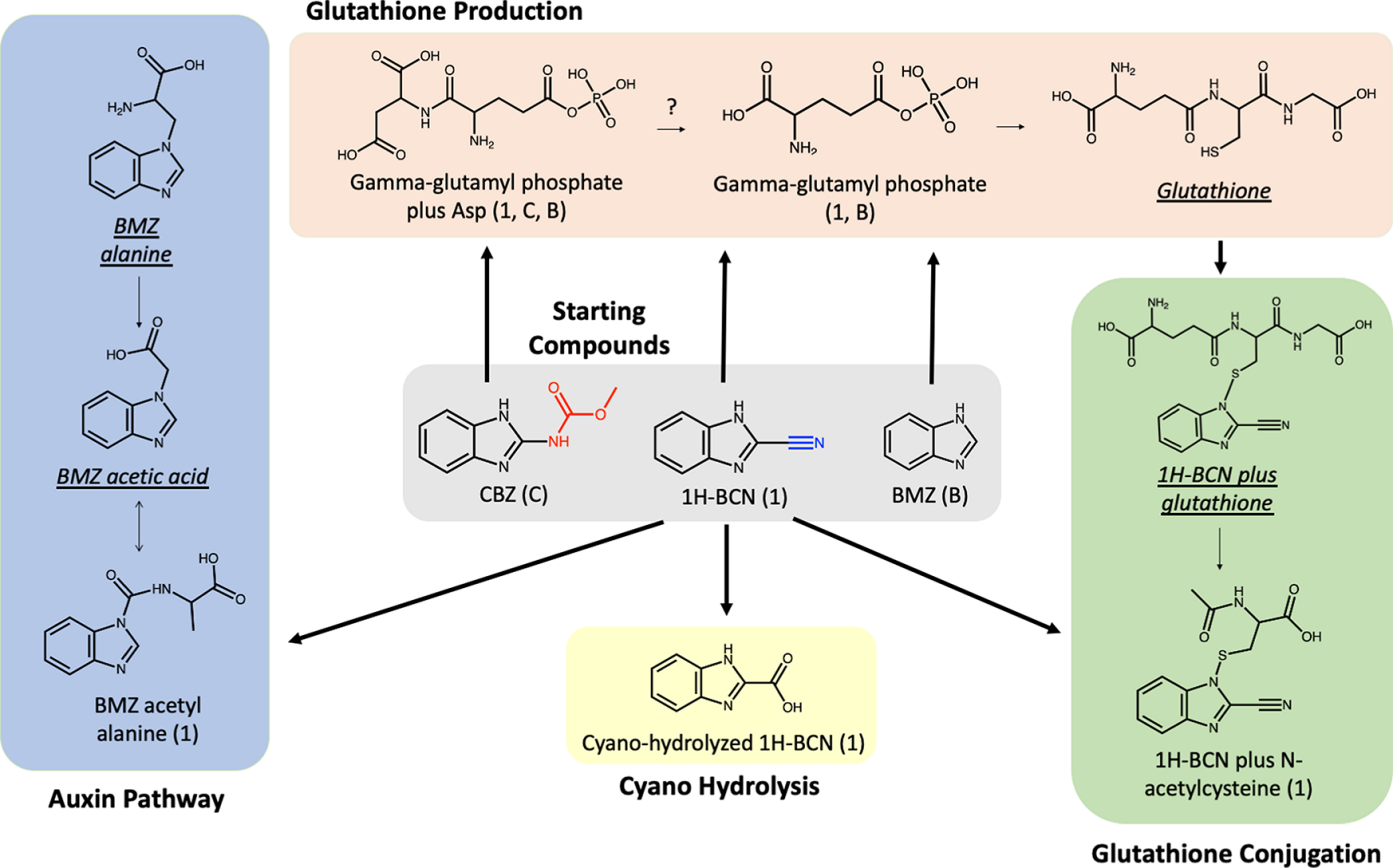
Proposed pathways for *Arabidopsis* metabolism of CN-BZ, Carb-BZ, and BZ. Major observed metabolites with a ≥100-fold change between treatment and unexposed plant control and an accurate mass deviation of <10 ppm are pictured, along with inferred intermediates (in underlined italics) that were not among the major metabolites. Compounds identified as a level 5 accurate mass of interest are listed in the Supporting Information and are not shown here.

The purpose of integrating a metabolomics study into the uptake kinetics work was to determine if exposure of plants to TOrCs with varied functional groups that demonstrated significant differences in uptake kinetics also yielded differences in plant metabolism. Pathways represented in the plant metabolism of three fungicides (BZ, Carb-BZ, and CN-BZ) include endogenous glutathione production, glutathione conjugation, auxin synthesis, and cyano-hydrolysis (Figure 2). We considered only the most highly represented metabolites, i.e., ≥100-fold increased peak area following chemical exposure, yielding 14 compounds (metabolite details in the Supporting Information): six major proposed metabolites (error of <10 ppm) and eight unknown accurate masses of interest. We also detected 235 BZ metabolites, 229 Carb-BZ metabolites, and 48 CN-BZ metabolites with a ≥5-fold increase in peak area in exposed plants; thus, the impact on plant metabolism extends beyond the major metabolites we identify here.

Exposure of plants to all three tested benzimidazole-based compounds generated metabolites consistent with the glutathione production pathway^51^ (Figure 2). To the best of our knowledge, γ-glutamyl phosphate–amino acid conjugates have not been previously documented; however, glutathione production in plant cells is known to involve similar γ-glutamyl–amino acid conjugates.^51,52^ Increased glutathione production is likely due to glutathione conjugation with the benzimidazole compounds, a common phase II xenobiotic detoxification mechanism.^8,53−58^ Glutathione conjugation allows for the transport of the conjugate into the vacuole, sequestering the xenobiotic to limit harm to major plant metabolic processes.^55,56,59^ Indeed, CN-BZ-exposed plants produced CN-BZ conjugated with *N*-acetylcysteine, which is likely derived from CN-BZ conjugated with glutathione. Alteration of glutathione production represents an impact on endogenous plant metabolism rather than mere generation of conjugated products with the xenobiotic and may have broader biological implications such as the control of reactive oxygen species (ROS).^60,61^

The two remaining pathways occurred only in CN-BZ-exposed plants. BZ acetyl alanine is the proposed metabolite in the auxin pathway, with a presumed BZ–alanine conjugate in the pathway. BT, with a base structure similar to that of benzimidazoles, is known to conjugate with alanine in hydroponic *Arabidopsis* to form a tryptophan-like molecule.^18^ The putative BT alanine intermediate in that study was a low-represented intermediate, consistent with a similar conjugate not being among the major metabolites in this study. BT acetyl alanine has been subsequently reported in a field bioretention cell^62^ and crops,^41^ highlighting how reductionist hydroponic research can drive field-relevant discoveries. The final pathway represented by major metabolites (i.e., >100-fold change) was cyano-hydrolysis. Cyano groups in xenobiotics are known to undergo such hydrolysis during plant metabolism, e.g., -CN to -CONH_2_^63^ or -CN to -COOH.^54^ This reaction is catalyzed by the nitrilase superfamily of enzymes in plants, which are active in detoxifying xenobiotics among other roles.^64^ In contrast to the other compounds, a significant portion of the untransformed parent compound remained in the BZ-exposed plants; the reason for this phenomenon is not currently known.

### Environmental Implications

Using a systematic evaluation with representative compounds, we documented significant differences in plant uptake rate with changes in compound functional group properties and position. These initial findings are urgently needed to better predict the propensity for TOrC plant uptake based on key molecular features rather than a compound-by-compound approach. Strongly electronegative groups (i.e., nitro and cyano groups) corresponded with the fastest plant uptake, which may be due to energetic binding interactions with plant transporter proteins; however, we cannot currently elucidate mechanisms. Active TOrC plant uptake via possible transporter-mediated processes is an environmentally relevant interaction^18^ for which limited predictive capability currently exists.^28^ For example, plant uptake of some PFAS^65−67^ and biocides^44^ is thought to occur via active processes. Therefore, data from this study can inform plant uptake models that incorporate transporter-mediated uptake to more accurately represent plant–chemical interactions.^28^ Despite functional group differences yielding dramatic differences in plant uptake kinetics, the benzimidazole fungicides tested in this work all impacted endogenous glutathione production. Limitations of this work include the relatively small sample size for the suite of compounds in our experimental TOrCs; we aimed in this first-of-a-kind study to focus on using closely related compounds with slight structural differences to probe effects, but other compound classes may behave differently. Additionally, this is a reductionist hydroponic study, and the relationships presented here may be complicated or muted by other factors in the field (e.g., hydrophobicity, bioavailability, metabolism rates, ionization states). Nevertheless, our past fundamental hydroponic plant research^18^ has successfully translated to field observations,^41,62^ demonstrating transferability to plant–soil systems. Understanding how compound chemistry influences both plant uptake kinetics and plant metabolism allows for more efficacious phytoremediation efforts^55^ and can inform the risk to humans and livestock from exposure to crops irrigated with recycled water.^5^

## Supporting Information Available

Additional experimental details, materials and methods, supplementary results, chemical data, plant growth/extraction experimental details, analytical methods, experimental methods and design, analytical methods, kinetics and rate calculations, QSAR descriptors of numerical results, and high-resolution MS analysis and spectra (PDF)
