# Supplementary material for: Functional Group Properties and Position Drive Differences in Xenobiotic Plant Uptake Rates, but Metabolism Shares a Similar Pathway
Source: Environ Sci Technol Lett. 2023 Jun 21;10(7):596–603. doi: 10.1021/acs.estlett.3c00282 (PMC10339724; doi:10.1021/acs.estlett.3c00282)
Supplement: Supplementary file 1 — ez3c00282_si_001.pdf [file ez3c00282_si_001.pdf]

Supporting Information

Functional Group Properties and Position Drive Differences in Xenobiotic Plant Uptake Rates but Metabolism Shares a Similar Pathway

Claire P. Muerdter,[†,‡] *Megan M. Powers,*[†,‡] *Danielle T. Webb,*[†,‡] *Sraboni Chowdhury,*[†,‡] *Kaitlyn E. Roach,*[^] and *Gregory H. LeFevre*[†,‡,*]

[†] Department of Civil and Environmental Engineering, University of Iowa, 4105 Seamans Center, Iowa City, Iowa, 52242, United States

[‡] IIHR—Hydroscience and Engineering, University of Iowa, 100 C. Maxwell Stanley Hydraulics Laboratory, Iowa City, Iowa, 52242, United States

^ University of Iowa Secondary Student Training Program, Belin-Blank Center, 600 Blank Honors Center, Iowa City, IA 52242, United States

[*] Corresponding Author: gregory-lefevre@uiowa.edu; Phone: +319 335 5655, Department of Civil and Environmental Engineering, 4105 Seamans Center for Engineering, University of Iowa, Iowa City IA, 52242, United States

Supporting Information Table of Contents [70 pages]

Additional Methods Details 5

Chemicals 5

Table S1: Compounds used in this work 7

Seed Sterilization Procedure 9

Arabidopsis Growth Procedure 9

Plant Exposure Experiment Details 9

Figure S1: 11

Neural network plant uptake model 11

Plant Tissue Harvest Details 12

Plant Extraction for Metabolomics 12

Analytical Methods 13

Dipole Moment and Plant Uptake Correlation 13

Table S2: Dipole moment values for molecules in this work 13

Figure S2: Dipole (Debye) vs second order rate for the molecules used in this work 14

QSAR Descriptors 14

LC-MS/MS and MRM Transition Details 16

Table S3: LC-MS/MS and MRM Transition Details 16

Supplementary Results 26

Figure S3: 7-day 1A-BT and 10-day benzimidazole plant uptake data 26

Figure S4: 1A-BZ 48-hour plant uptake data 27

Figure S5: 2N-BZ 48-hour plant uptake data 27

Figure S6: 2A7Cl-BZ and 2Cl-BZ 48-hour plant uptake data 28

Table S4: Sorption results 28

Table S5: Uptake rate k values, including abiotic control info, for compounds in this study 29

Table S6: p-values for comparisons between benzimidazole uptake rates from Figure 1 30

Table S7: QSAR descriptors as obtained from Spartan'20 parallel suite computations 31

Table S8: QSAR data obtained in Spartan '20 for the fungicides studied 32

Figure S7: Principal Component Analysis (PCA) of QSAR descriptors and uptake rate constants 36

Table S9: Pearson rho correlations and p-values (for descriptors with p <0.1) for the correlation of QSAR parameters to experimental rate constants (for all compounds) 37

Plant Metabolomics Settings 37

Compound Discoverer Analysis 38

Figure S8: Workflow tree showing the components of the Compound Discoverer automated analysis (screenshot). 39

Workflow node details: 39

Volcano Plots of Metabolite Results 43

Figure S9: Volcano plots of features changed in plant tissue exposed 44

Metabolite Details 45

Table S10: Metabolites Shared Between All Three Fungicides (Benzimidazole, Carbendazim, and CN-BZ) 45

Table S11: Metabolites Shared by Benzimidazole and CN-BZ **46**

Table S12: Benzimidazole-Only Metabolites **47**

Table S13: CN-BZ-Only Metabolites **47**

Table S14: CDM-Only Metabolite **49**

Metabolite Mass Spectra and Structures **50**

Metabolites Shared Between All Three Fungicides 50

Gamma-glutamyl phosphate with Asp 50

Figure S10: Mass Spectra (a: MS1, b: MS2) and proposed structures for gamma-glutamyl phosphate with Asp 51

Unknown M209 51

Figure S11: Mass spectra (MS1 only) for unknown M209 51

Unknown M275 52

Figure S12: Mass Spectra (a and b: MS1, c: MS2) and proposed structures for unknown M275 53

Metabolites Shared Between Benzimidazole and CN-BZ 53

Unknown M412 53

Figure S13: Mass Spectra (a: MS1 b: MS2) and proposed structures for unknown M412 54

Unknown M427 54

Figure S14: Mass Spectra (a: MS1 b: MS2) and proposed structures for unknown M427 55

Unknown M437 55

Figure S15: Mass Spectra (a: MS1 b: MS2 c: Additional MS1 peak) and proposed structures for unknown M437 56

L-gamma-glutamyl phosphate 57

Figure S16: Mass Spectra (a: MS1 b: MS2) and proposed structures for L-gamma-glutamyl phosphate 57

Benzimidazole-Only Metabolites 58

Unknown M297 58

Figure S17: Mass Spectra (a: MS1 b: MS2) and proposed structures for unknown M297 58
118.05327 Accurate Mass: Benzimidazole (parent compound) 59
Figure S19: Results of standard addition for benzimidazole 60
Figure S20: Mass spectra of standard addition for benzimidazole (a: Standard addition MS spectra b: Standard MS spectra).. 61
CN-BZ-Only Metabolites.. 62
Unknown M179 62
Figure S21: Mass spectra of unknown M179 (a: MS1 b: MS1, additional peak in negative mode c: MS2) 63
Cyano-hydrolyzed CN-BZ 63
Figure S22: Mass spectra of cyano-hydrolyzed CN-BZ (a: MS1 b: MS2).. 64
CN-BZ plus N-acetylcysteine 65
Figure S23: Mass spectra of cyano-hydrolyzed CN-BZ plus N-acetylcysteine (a: MS1 b: MS2) 65
234.08989 Accurate Mass: BZ acetyl alanine 66
Figure S24: Mass spectrum (MS1) of BZ acetyl alanine.. 66
CDM-Only Metabolites... 67
Unknown M780 67
Figure S25: Mass spectra of unknown M780 (a: MS1 b: MS2)... 67

ADDITIONAL METHODS DETAILS

Chemicals

Benzimidazole and benzotriazole-based compounds were used in this work because they are taken up by plants,[1,2] with benzotriazole uptake known to exceed transpiration rate,[1] and their base structures vary by only a single nitrogen in the heterocyclic ring. Benzimidazole derivatives

are commonly used fungicides[3–5] and benzotriazole is a widely used corrosion inhibitor;[6] both have high solubilities (Table S1) and are present in environmental waters.[7]

Chemicals used in these experiments include (Table S1): Benzimidazole ("BZ," CAS 51-17-2, ACROS Organics, 98%), 2-aminobenzimidazole ("2A-BZ," CAS 934-32-7, Aldrich, 97%), 1-aminobenzimidazole ("1A-BZ," CAS 6299-92-9, Aldrich, 97% purity), 2-cyanobenzimidazole ("CN-BZ," CAS 6868-37-7, Chem Bridge, 100%), 2-chlorobenzimidazole ("2Cl-BZ," CAS 4857-06-1, Sigma-Aldrich (592277), purity 98%), 2-nitrobenzimidazole ("2N-BZ," CAS 5709-67-1, AmBeed, 95%+ purity), 2-amino-7-chloro-benzimidazole ("2A7Cl-BZ," CAS 701-14-4, Sigma-Aldrich (761672), purity 95%), carbendazim ("Carb-BZ," CAS 10605-21-7, Aldrich, 97%), thiabendazole ("TBZ," CAS 148-79-8, Sigma, >99% purity), benzotriazole ("BT," CAS 95-14-7, Fluka Analytical, ≥98% purity), and 1-aminobenzotriazole ("1A-BT," CAS 1614-12-6, TCI, >98% purity). All LC-MS/MS solvents (acetonitrile, water, and formic acid) were Fisher Optima LC/MS grade.

Liquid plant medium was generated by combining (for 1 L medium): 4.43 g Murashige and Skoog (MS) Basal Medium powder (PhytoTech Labs M519), 0.5 g 2-morpholin-4-ylethanesulfonic acid (MES) free acid monohydrate (Fisher), and deionized water to ~900 mL. Then 5 g sucrose (Research Products International) and DI water to 1 L was added. The pH was checked and adjusted to a 5.7 as needed with potassium hydroxide or hydrochloric acid. Before experimental use, the medium was filter-sterilized using a bottle top filter (Corning #431118, 0.22 µm pore size) into an autoclaved bottle.

Table S1: Compounds used in this work (purity, source, etc. used in this work described above)

Compound Name *(Abbreviation Used)*	***CAS Number***	***Molecular Weight (g/mol)***	***Log K_{OW} (Experimental unless marked with an *, indicating a calculated value)***	***Structure***
**Benzimidazole** (BZ)	51-17-2	118.1	1.32[8]	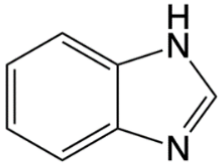
**2-aminobenzimidazole** (2A-BZ)	934-32-7	133.15	0.91[8]	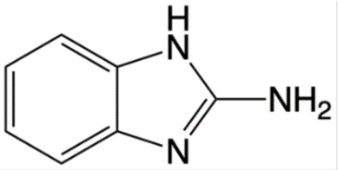
**1-amino benzimidazole** (1A-BZ)	6299-92-9	133.15	1.2*[9]	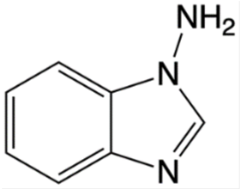
**2-cyanobenzimidazole** (CN-BZ)	6868-37-7	143.15	1.3*[10]	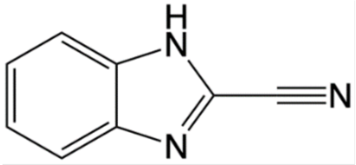
**2-chlorobenzimidazole** (2Cl-BZ)	4857-06-1	152.58	2.2*[11]	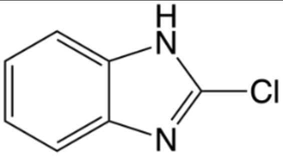
**2-nitrobenzimidazole** (2N-BZ)	5709-67-1	163.13	1.4[12]*	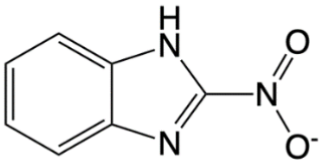

Compound Name *(Abbreviation Used)*	***CAS Number***	***Molecular Weight (g/mol)***	***Log K_{OW} (Experimental unless marked with an *, indicating a calculated value)***	***Structure***
**2-amino-7-chloro-benzimidazole** (2A7Cl-BZ)	701-14-4	167.59	1.8*[13]	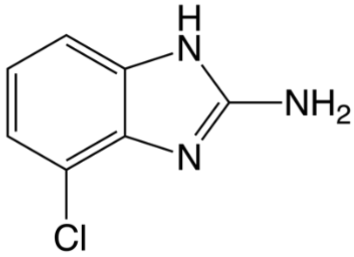
**Carbendazim** (Carb-BZ)	10605-21-7	191.19	1.37[14]	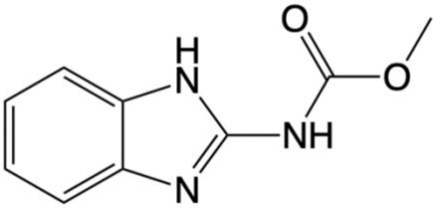
**Thiabendazole** (TBZ)	148-79-8	201.25	2.3[15]	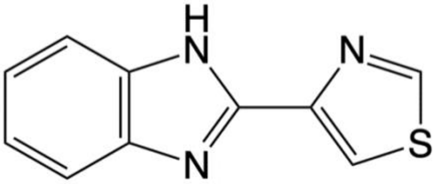
**Benzotriazole** (BT)	95-14-7	119.12	1.44[8]	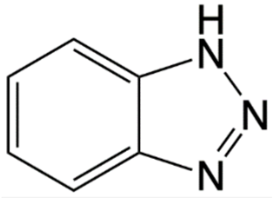
**1-aminobenzotriazole** (1A-BT)	1614-12-6	134.14	0.9[16]*	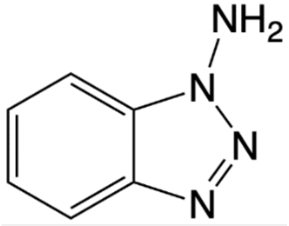

Seed Sterilization Procedure

A previously published seed sterilization procedure was used with *Arabidopsis thaliana* Columbia ecotype “0” (Col-0) seeds,[18] with the following minor modifications:

- Instead of conducting the procedures over a flame, seed sterilization was conducted in a biosafety cabinet.
- Rather than 50 µL of seed, between 10 and 50 µL of seed were used, depending on the quantity of plants required for the experiment.

Arabidopsis Growth Procedure

A previously published Arabidopsis growth procedure[18] was used to grow up the sterilized seeds, before exposure to isothiazolinones, with the following modifications:

- 30 +/- 2 seeds per box were used, placed into boxes with a pipette tip and visually counted
- Growth chamber temperatures were 23°C during the light period and 21°C during the dark period
- Plants were grown for 11–13 days before exposure to benzimidazole or benzotriazole based compounds. The average dry biomass of plants (per box) after this growth period was 0.03±0.005g (ave±stdev).

Plant Exposure Experiment Details

The exposure experiments were modeled on previous work.[1,17,18] After a 11–13 day period of growth in unspiked sterile hydroponic medium, the boxes were taken from the growth chamber into a biological safety cabinet and the following procedures conducted using sterile technique.

A master mix of medium (i.e., a large volume of medium was prepared to ensure consistency rather than each box being prepared separately) was spiked with the benzimidazole or benzotriazole of interest (one compound per treatment). 3–4 samples were taken from the master mix, at 0.6 mL each, and filtered with nylon filters (0.2 µm, 13 mm diameter, mdi SY13NN) into LC vials. These medium samples were frozen at -20°C at the end of each timepoint and kept frozen until analysis.

After master mix sampling for the t=0 timepoint, the microporous tape was removed from each plant Magenta box and the box tilted to allow for the medium to leave the box while the plant tissue remained in the box. The box lid was then removed and freshly spiked plant growth medium was added to each box, at 25 mL per box. The box lid and microporous tape were then replaced.

Additionally, an abiotic control was created at t=0 for each treatment. Each control replicate consisted of the same amount of master mix of medium as the plant boxes (25 mL), pipetted into a washed and autoclaved Magenta box. The lid was replaced and microporous tape applied in the same manner as the plant boxes. Each treatment and control was conducted at n=3–4.

Except for sampling, boxes were kept in the Percival growth chamber alternating between 16 hours light at 23° C and 8 hours dark at 21° C. Relative humidity was maintained at 50%. All sampling was conducted using sterile technique in the biosafety cabinet, as described above.

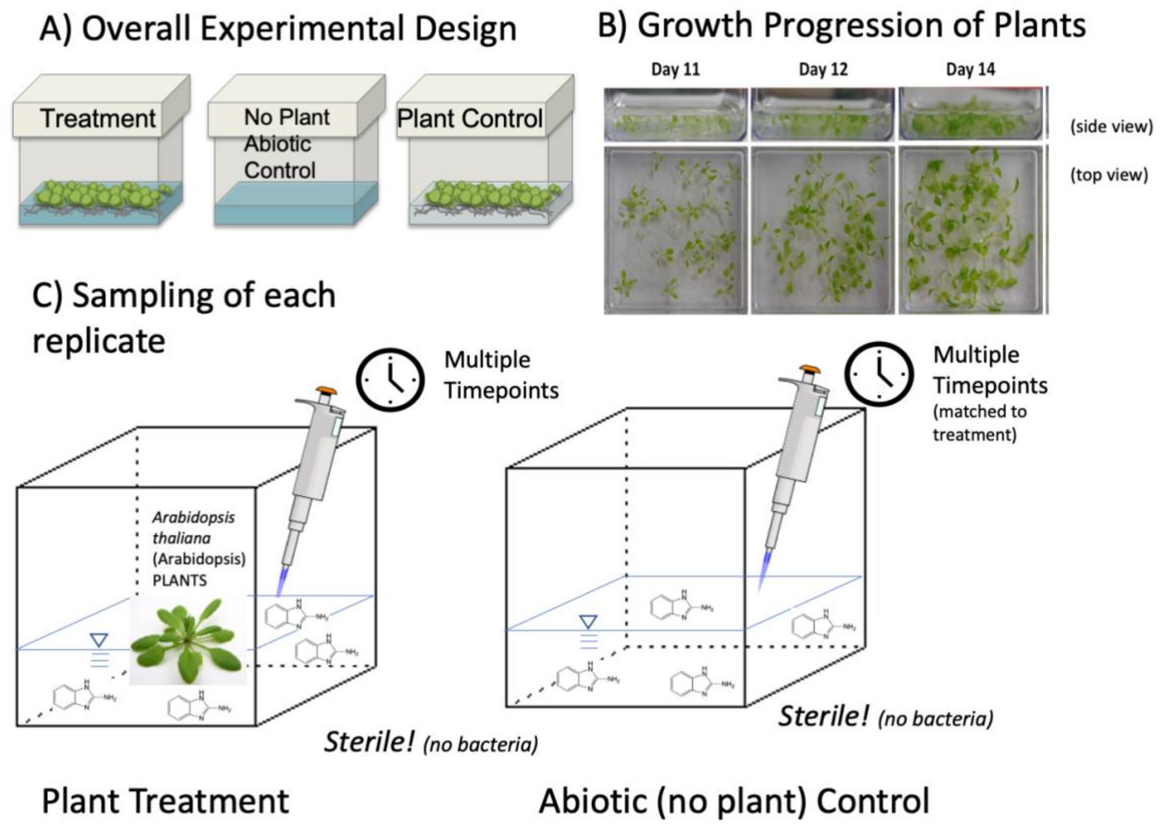

Figure S1: Experimental design for the plant exposure experiments. In all cases the hydroponic medium was filter-sterilized to exclude any bacteria. (A): Overall experimental design, showing the treatment with added trace organic contaminant and plant, no plant abiotic control containing the trace organic contaminant but no plant (to quantify any abiotic losses), and plant (positive) controls with no chemical exposure to demonstrate that plants growth conditions were adequate for healthy growth. Sorption controls (not illustrated) used plant tissues grown without contaminant exposure, which were exposed to contaminants for five minutes and then sampled to quantify immediate short-term sorption to biomass and exclude the majority, if not all, of plant uptake. (B): Photo illustrations [photos: LeFevre] of hydroponic Arabidopsis seedlings on days 11, 12, and 14. (C): Detailed illustrated experimental design diagram of the sampling scheme for each Magenta box replicate, with paired treatment and abiotic controls.

Neural network plant uptake model

The authors of a previously published plant uptake model focused on TSCF prediction[8] generously provided their model's TSCF predictions of BZ and Carb-BZ by inputting those molecules' log K_{OW}, molecular weight, hydrogen bond donor, hydrogen bond acceptor, rotatable bonds, and polar surface area properties into the model.

Plant Tissue Harvest Details

The microporous tape was removed from each box and the box tilted with the lid still on to allow for the medium to drain out while the plants were retained in the box. The box was then inverted onto a clean paper towel, and the box removed. The plant tissue was gently patted with the paper towel to remove any remaining medium. Clean tweezers were then used to move the tissue into 1.5 or 2 mL microcentrifuge tubes with locking lids, with the tweezers cleaned with ethanol between each box. Plant tissue was then frozen at -20 °C until overnight freeze drying and extraction (below).

Plant Extraction for Metabolomics

The following procedure from previous work[1,18] was used after overnight freeze drying:

A single stainless steel homogenization bead (5 mm) and 1.0 mL of 1:1 methanol/water solution were added to freeze-dried plant tissues in a microcentrifuge tube. The tubes were frozen at −80 °C for 30 min. Samples were thawed and placed on a Retsch mixer mill for 5 min at 30 Hz. The samples were then sonicated for 10 min, vortexed for 1 min, and centrifuged at 10 000 × g for 10 min. Following centrifugation the supernatant was removed with a 22G x 1 ½ BD precision glide needles needle syringe and filtered through a 0.2 μm, 13 mm diameter PFTE filter (mdi) into an empty autosampler vial. The extraction procedure was repeated sequentially two additional times by adding only 0.5 mL (rather than 1.0 mL) of the methanol:water solution for each subsequent extraction and otherwise exactly repeating the extraction procedure (i.e., homogenization, sonication, vortex, centrifugation, filtration). All three fractions were combined in a single autosampler vial for analysis.

Analytical Methods

Samples were analyzed via high performance liquid chromatography (Agilent 1260) coupled to a triple quadrupole mass spectrometer (LC-MS/MS; Agilent 6460 Triple Quadrupole MS with MassHunter, version B.07.00) operating in multiple reaction monitoring (MRM) positive ionization mode. LC-MS/MS method details are given below. The sample tray was kept at 4 °C. Peak area was quantified from chromatograms using Agilent MassHunter Qualitative Analysis software and used as a proxy for concentration.

Standards were found to reliably give a peak down to 500 ng/L. Between non-detect values and the lowest non-zero standard for the given MS/MS run, the standard curve was extended and used to estimate the concentration. This was deemed appropriate because the lowest measured non-zero samples had signal to noise ratios of 12:1 (2N-BZ), 8:1 (CN-BZ) and 5:1 (BT).

Dipole Moment and Plant Uptake Correlation

Dipole moment calculated using https://molcalc.org/.

MolCalc Citation information: J. H. Jensen and J. C. Kromann, The Molecule Calculator: A Web Application for Fast Quantum Mechanics-Based Estimation of Molecular Properties, J. Chem. Educ., 2013, 90 (8), pp 1093–1095. DOI: 10.1021/ed400164n

Table S2: Dipole moment values for molecules in this work

Molecule	Dipole (Debye)
1A-BT	3.25
1A-BZ	3.69
2Cl-BZ	4.49
BZ	4.57
2A-BZ	4.48
BT	4.97

Carb-BZ	Could not calculate*
CN-BZ	Could not calculate*
2N-BZ	Could not calculate*
2A7Cl-BZ	Could not calculate*

*Dipole moment values calculated using molecalc.org could not converge for some compounds.

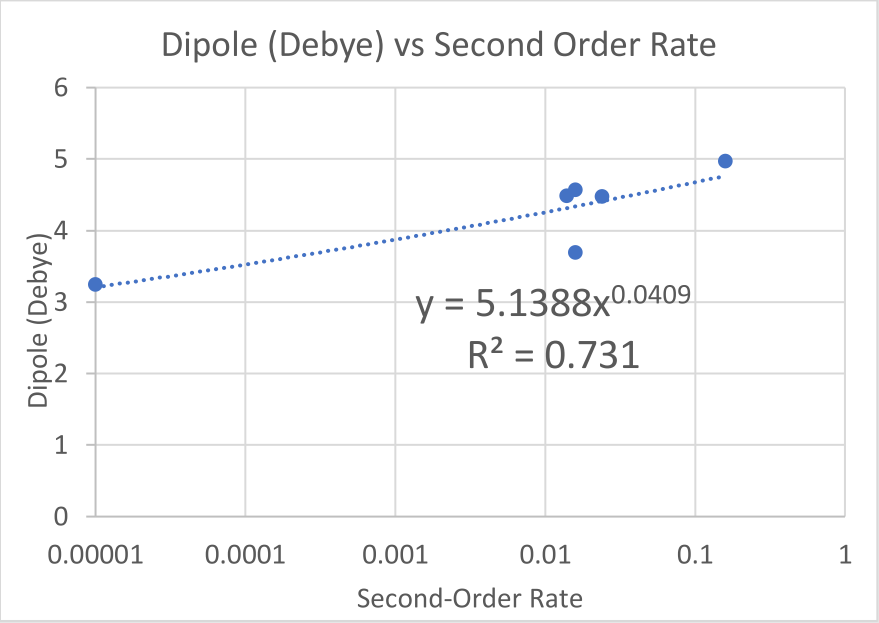

Figure S2: Dipole (Debye) vs second order rate for the molecules used in this work

QSAR Descriptors

Quantitative structure-activity relationships (QSAR) were generated with the Spartan '20 Parallel Suite Quantum Mechanics Program (Win/64b), release 1.0.0 (Dec 15, 2020) Wavefunction, Inc. QSAR parameters were determined based on each compound's ground state equilibrium geometry in water (we note that these computations can be impacted by the redox conditions, which can introduce some uncertainty). Computations were performed using a ωB97X-D/6-31G* (method/basis set) and restricted hybrid model (SCF model HF-DFT using Pulay DIIS + Geometric direct minimization, polarizable continuum solvation model). Electrostatic potential and local ionization potential were mapped using the Spartan '20 Parallel Suite graphics program

at a resolution of 0.0002 e/au^3. A total of 33 descriptors were obtained directly from Spartan and describe the electrical, quantum, and geometric molecular properties of a given compound (Tables S7 and S8).

LC-MS/MS and MRM Transition Details

Table S3: LC-MS/MS and MRM Transition Details

Target Compound(s)	Chromatography	Method Parameters	Qualitative or Quantitative Transition	Q1 *m/z*	Q3 *m/z*	Dwell time (ms)	Fragmentor voltage (V)	Collision energy (V)	Cell Accelerator Voltage (V)
Benzimidazole (BZ)	0.2 mL min^{-1} method for 20 min.<br><br>Mobile phase A = Fisher Optima LC/MS Water with 0.1% Optima LC/MS grade formic acid<br><br>Mobile phase B = Fisher Optima LC/MS acetonitrile with 0.1% Optima LC/MS grade formic acid<br><br>Gradient:<br>• 0 min: 95% A, 5% B<br>• 6.0 min: 30% A, 70% B<br>• 10.0 min: 10% A, 90% B<br>• 15.0 min: 95% A, 5% B<br><br>Approximate retention time: 10.2 min<br><br>Column used: Agilent Eclipse Plus C18 (5 µm, 4.6 x 150 mm)	Injection volume: 10 µL<br>Column temperature: 50 °C<br>Gas temperature: 300 °C<br>Gas flow: 5 L min^{-1}<br>Nebulizer pressure: 20 PSI<br>Sheath gas temperature: 250 °C<br>Sheath gas flow: 11 L min^{-1}<br>Positive and negative capillary voltage: each 3,500 V<br>Positive and negative nozzle voltage: each 500 V<br>Polarity for all transitions: positive	Quantitative	119.06	65.1	200	69	36	7
			Qualitative	119.06	92.1	200	69	27	7

Target Compound(s)	Chromatography	Method Parameters	Qualitative or Quantitative Transition	Q1 *m/z*	Q3 *m/z*	Dwell time (ms)	Fragmentor voltage (V)	Collision energy (V)	Cell Accelerator Voltage (V)
2-aminobenzimidazole (2A-BZ)	0.2 mL min^{-1} method for 20 min.<br><br>Mobile phase A = Fisher Optima LC/MS Water with 0.1% Optima LC/MS grade formic acid<br><br>Mobile phase B = Fisher Optima LC/MS acetonitrile with 0.1% Optima LC/MS grade formic acid<br><br>Gradient:<br>• 0 min: 95% A, 5% B<br>• 6.0 min: 30% A, 70% B<br>• 10.0 min: 10% A, 90% B<br>• 15.0 min: 95% A, 5% B<br><br>Approximate retention time: 16.0 min<br><br>Column used: Agilent Eclipse Plus C18 (5 µm, 4.6 x 150 mm)	Injection volume: 10 µL<br>Column temperature: 50 °C<br>Gas temperature: 300 °C<br>Gas flow: 5 L min^{-1}<br>Nebulizer pressure: 35 PSI<br>Sheath gas temperature: 250 °C<br>Sheath gas flow: 11 L min^{-1}<br>Positive and negative capillary voltage: each 3,500 V<br>Positive and negative nozzle voltage: each 500 V<br>Polarity for all transitions: positive	Quantitative	134.07	65.1	200	118	36	7
			Qualitative	134.07	92.1	200	118	24	7

Target Compound(s)	Chromatography	Method Parameters	Qualitative or Quantitative Transition	Q1 *m/z*	Q3 *m/z*	Dwell time (ms)	Fragmentor voltage (V)	Collision energy (V)	Cell Accelerator Voltage (V)
2-cyanobenzimidazole (CN-BZ)	0.2 mL min^{-1} method for 20 min.<br><br>Mobile phase A = Fisher Optima LC/MS Water with 0.1% Optima LC/MS grade formic acid<br><br>Mobile phase B = Fisher Optima LC/MS acetonitrile with 0.1% Optima LC/MS grade formic acid<br><br>Gradient:<br>• 0 min: 95% A, 5% B<br>• 6.0 min: 30% A, 70% B<br>• 10.0 min: 10% A, 90% B<br>• 15.0 min: 95% A, 5% B<br><br>Approximate retention time: 18.4 min<br><br>Column used: Agilent Eclipse Plus C18 (5 µm, 4.6 x 150 mm)	Injection volume: 5 µL<br>Column temperature: 50 °C<br>Gas temperature: 300 °C<br>Gas flow: 5 L min^{-1}<br>Nebulizer pressure: 25 PSI<br>Sheath gas temperature: 250 °C<br>Sheath gas flow: 11 L min^{-1}<br>Positive and negative capillary voltage: each 3,500 V<br>Positive and negative nozzle voltage: each 500 V<br>Polarity for all transitions: positive	Quantitative	144.06	65.1	200	124	33	7
			Qualitative	144.06	92.0	200	124	25	7

Target Compound(s)	Chromatography	Method Parameters	Qualitative or Quantitative Transition	Q1 *m/z*	Q3 *m/z*	Dwell time (ms)	Fragmentor voltage (V)	Collision energy (V)	Cell Accelerator Voltage (V)
2-chlorobenzimidazole (2Cl-BZ)	0.2 mL min^{-1} method for 20 min.<br><br>Mobile phase A = Fisher Optima LC/MS Water with 0.1% Optima LC/MS grade formic acid<br><br>Mobile phase B = Fisher Optima LC/MS acetonitrile with 0.1% Optima LC/MS grade formic acid	Injection volume: 10 µL<br>Column temperature: 50 °C<br>Gas temperature: 300 °C<br>Gas flow: 5 L min^{-1}<br>Nebulizer pressure: 35 PSI<br>Sheath gas temperature: 250 °C<br>Sheath gas flow: 11 L min^{-1}<br>Positive and negative capillary voltage:	Quantitative	154.61	118.1	200	126	28	4

Target Compound(s)	Chromatography	Method Parameters	Qualitative or Quantitative Transition	Q1 *m/z*	Q3 *m/z*	Dwell time (ms)	Fragmentor voltage (V)	Collision energy (V)	Cell Accelerator Voltage (V)
	Gradient:<br>• 0 min: 95% A, 5% B<br>• 6.0 min: 30% A, 70% B<br>• 10.0 min: 10% A, 90% B<br>• 15.0 min: 95% A, 5% B<br><br>Approximate retention<br><br>time: 18.8 min<br><br>Column used: Agilent Eclipse Plus C18 (5 μm, 4.6 x 150 mm)	each 3,500 V<br>Positive and negative nozzle voltage:<br>each 500 V<br>Polarity for all transitions: positive	Qualitative	154.61	65.2	200	126	40	4
2-amino-7-chloro-benzimidazole (2A7Cl-BZ)	0.2 mL min^{-1} method for 23 min.<br><br>Mobile phase A = Fisher Optima LC/MS Water with 0.1% Optima LC/MS grade formic acid<br><br>Mobile phase B = Fisher Optima LC/MS acetonitrile with 0.1% Optima LC/MS grade formic acid	Injection volume:<br>10 μL<br>Column temperature:<br>50 °C<br>Gas temperature:<br>350 °C<br>Gas flow: 5 L min^{-1}<br>Nebulizer pressure:<br>45 PSI<br>Sheath gas temperature: 250 °C<br>Sheath gas flow:<br>11 L min^{-1}	Quantitative	168.04	133	200	135	24	7
			Qualitative	168.04	105	200	135	36	7

Target Compound(s)	Chromatography	Method Parameters	Qualitative or Quantitative Transition	Q1 *m/z*	Q3 *m/z*	Dwell time (ms)	Fragmentor voltage (V)	Collision energy (V)	Cell Accelerator Voltage (V)
	Gradient:<br>• 0 min: 95% A, 5% B<br>• 6.0 min: 30% A, 70% B<br>• 10.0 min: 10% A, 90% B<br>• 15.0 min: 95% A, 5% B<br><br>Approximate retention time: 16.0 min<br><br>Column used: Agilent Eclipse Plus C18 (5 µm, 4.6 x 150 mm)	Positive capillary voltage: 4,000 V<br>Negative capillary voltage: 3,500 V<br>Positive and negative nozzle voltage: each 500 V<br>Polarity for all transitions: positive							
Carbendazim (Carb-BZ)	0.2 mL min^{-1} method for 20 min.<br><br>Mobile phase A = Fisher Optima LC/MS Water with 0.1% Optima LC/MS grade formic acid<br><br>Mobile phase B = Fisher Optima LC/MS acetonitrile with 0.1% Optima LC/MS grade formic acid<br><br>Gradient:<br>• 0 min: 95% A, 5% B	Injection volume: 10 µL<br>Column temperature: 50 °C<br>Gas temperature: 300 °C<br>Gas flow: 5 L min^{-1}<br>Nebulizer pressure: 35 PSI<br>Sheath gas temperature: 250 °C<br>Sheath gas flow: 11 L min^{-1}<br>Positive and negative capillary voltage: each 3,500 V<br>Positive and negative nozzle voltage:	Quantitative	192.08	160.0	200	74	16	7
			Qualitative	192.08	132.0	200	74	36	7

Target Compound(s)	Chromatography	Method Parameters	Qualitative or Quantitative Transition	Q1 *m/z*	Q3 *m/z*	Dwell time (ms)	Fragmentor voltage (V)	Collision energy (V)	Cell Accelerator Voltage (V)
	• 6.0 min: 30% A, 70% B<br>• 10.0 min: 10% A, 90% B<br>• 15.0 min: 95% A, 5% B<br><br>Approximate retention time: 16.2 min<br><br>Column used: Agilent Eclipse Plus C18 (5 µm, 4.6 x 150 mm)	each 500 V<br>Polarity for all transitions: positive							
Thiabendazole (TBZ)	0.2 mL min^{-1} method for 20 min<br><br>Mobile phase A = Fisher Optima LC/MS Water with 0.1% Optima LC/MS grade formic acid<br><br>Mobile phase B = Fisher Optima LC/MS acetonitrile with 0.1% Optima LC/MS grade formic acid<br><br>Gradient:<br>• 0 min: 95% A, 5% B<br>• 6.0 min: 30% A, 70% B<br>• 10.0 min: 10% A, 90% B	Injection volume: 10 µL<br>Column temperature: 50 °C<br>Gas temperature: 300 °C<br>Gas flow: 9 L min^{-1}<br>Nebulizer pressure: 35 PSI<br>Sheath gas temperature: 250 °C<br>Sheath gas flow: 8 L min^{-1}<br>Positive and negative capillary voltage: each 3,500 V<br>Positive and negative nozzle voltage: each 500 V<br>Polarity for all transitions: positive	Quantitative	202.05	175.0	200	65	24	7
			Qualitative	202.05	131.0	200	65	36	7

Target Compound(s)	Chromatography	Method Parameters	Qualitative or Quantitative Transition	Q1 *m/z*	Q3 *m/z*	Dwell time (ms)	Fragmentor voltage (V)	Collision energy (V)	Cell Accelerator Voltage (V)
	• 15.0 min: 95% A, 5% B<br><br>Approximate retention time: 16.1 min<br><br>Column used: Agilent Eclipse Plus C18 (5 µm, 4.6 x 150 mm)								
1-amino benzimidazole (1A-BZ)	0.2 mL min^{-1} method for 22 min<br><br>Mobile phase A = Fisher Optima LC/MS Water with 0.1% Optima LC/MS grade formic acid<br><br>Mobile phase B = Fisher Optima LC/MS acetonitrile with 0.1% Optima LC/MS grade formic acid<br><br>Gradient:<br>• 0 min: 95% A, 5% B<br>• 6.0 min: 30% A, 70% B<br>• 10.0 min: 10% A, 90% B<br>• 15.0 min: 95% A, 5% B<br><br>Approximate retention time: 15.6 min	Injection volume: 10 µL<br>Column temperature: 50 °C<br>Gas temperature: 350 °C<br>Gas flow: 5 L min^{-1}<br>Nebulizer pressure: 45 PSI<br>Sheath gas temperature: 250 °C<br>Sheath gas flow: 11 L min^{-1}<br>Positive capillary voltage: 4,000 V<br>Negative capillary voltage: 3,500 V<br>Positive and negative nozzle voltage: each 0 V<br>Polarity for all transitions: positive	Quantitative	134.07	65.0	200	100	25	7
			Qualitative	134.07	92.0	200	100	20	7

Target Compound(s)	Chromatography	Method Parameters	Qualitative or Quantitative Transition	Q1 *m/z*	Q3 *m/z*	Dwell time (ms)	Fragmentor voltage (V)	Collision energy (V)	Cell Accelerator Voltage (V)
	Column used: Agilent Eclipse Plus C18 (5 µm, 4.6 x 150 mm)								
2-nitrobenzimidazole (2N-BZ)	Same as 1A-BZ except approximate retention time: 19.4 min	Same as 1A-BZ	Quantitative	164.05	118.0	200	90	20	7
			Qualitative	164.05	133.9	200	90	12	7
Benzotriazole (BT)	0.2 mL min^{-1} method for 18 min.<br><br>Mobile phase A = Fisher Optima LC/MS Water with 0.1% Optima LC/MS grade formic acid<br><br>Mobile phase B = Fisher Optima LC/MS acetonitrile with 0.1%	Injection volume: 10 µL<br>Column temperature: 50 °C<br>Gas temperature: 300 °C<br>Gas flow: 8 L min^{-1}<br>Nebulizer pressure: 40 PSI<br>Sheath gas temperature: 250 °C<br>Sheath gas flow: 5 L min^{-1}	Quantitative	120.06	65.1	200	75	24	4

Target Compound(s)	Chromatography	Method Parameters	Qualitative or Quantitative Transition	Q1 *m/z*	Q3 *m/z*	Dwell time (ms)	Fragmentor voltage (V)	Collision energy (V)	Cell Accelerator Voltage (V)
	Optima LC/MS grade formic acid<br><br>Gradient:<br>• 0 min: 90% A, 10% B<br>• 4.0 min: 20% A, 80% B<br>• 6.5 min: 80% A, 20% B<br>• 9.1 min: 90% A, 10% B<br><br>Approximate retention time: 15.0 min<br><br>Column used: Agilent Eclipse Plus C18 (5 µm, 4.6 x 150 mm)	Positive and negative e capillary voltage: each 3,500 V<br>Positive and negative nozzle voltage: each 500 V<br>Polarity for all transitions: positive	Qualitative	120.06	92	200	75	16	4
1-aminobenzotriazole (1A-BT)	Same as BT except approximate retention time: 15.1 min	Same as BT	Quantitative	135.15	80	200	90	16	4
			Qualitative	135.15	53.1	200	90	28	4

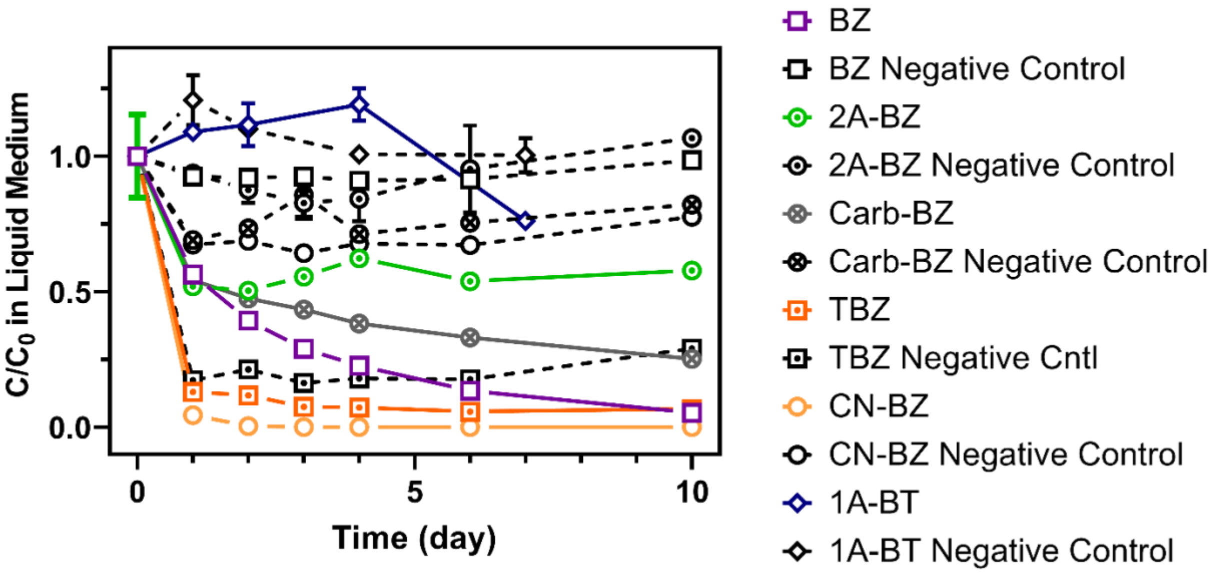

Figure S3: 7-day 1A-BT and 10-day benzimidazole plant uptake data. Nominal C_0 of 20 µg/L for each compound, with each compound tested separately with both plant exposure treatment and negative control. n=3–4, error bars represent standard error and are obscured by symbols for some datapoints. Plant treatments are shown with solid lines, corresponding negative (no-plant abiotic) controls are shown in black dashed lines with matching symbol shape to their corresponding plant treatment.

Notes on these results: For a limited number of the compounds, data were collected to 10 days after exposure to the chemicals. We focused on the 48h period in the main presentation because contact time between plants and water is often limited (e.g., irrigation, bioinfiltration). These results revealed further separation of some of the 'moderate' removal group of compounds. 2A-BZ maintains the moderate removal found at 48 hours, ending at a C/C_0 of 58% after 10 days. In contrast, the C/C_0 of Carb-BZ was 25% at 10 days, and BZ is almost completely removed by 10 days (C/C_0=5%). We also note differences within compounds in the 'moderate' removal group at the 48h point. For example, although 2-CBZ and 1-ABZ group together when compared with the 'no removal' and 'greatest removal' groups, at 48 hours each have a significantly higher C/C_0 than Carb-BZ and 2-ABZ (both compounds vs. Carb-BZ p=0.03, both compounds vs. 2-ABZ p=0.0007), and BZ's C/C_0 is significantly higher than 2-ABZ (p=0.02). Please also note that plant uptake is focused on depletion from the hydroponic medium; subsequent in planta degradation of compounds taken up by plants is not treated separately. Plant degradation of compounds outside the plant (i.e., via root exudates) has been previously shown to be not significant for other related TOrCs under aseptic hydroponic conditions.[1]

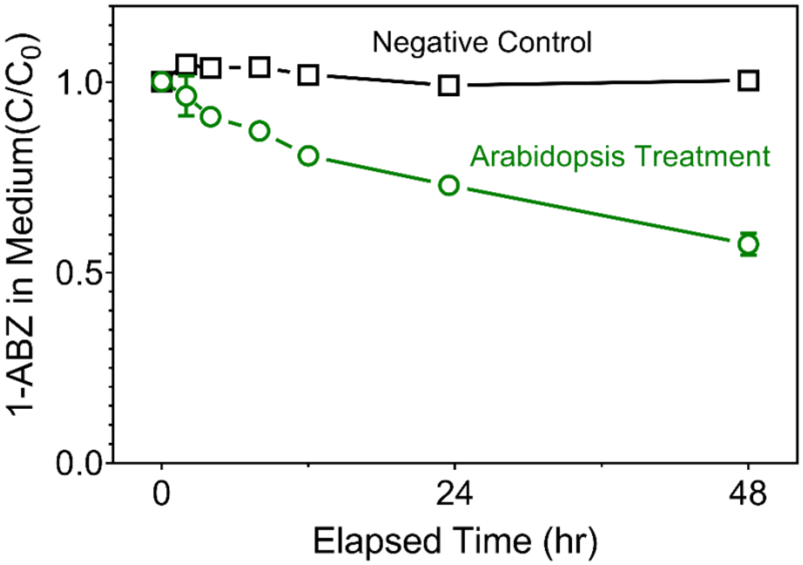

Figure S4: 1A-BZ 48-hour plant uptake data. Nominal C_0 of 20 µg/L. n=3–4, error bars represent standard error and are obscured by symbols for some datapoints.

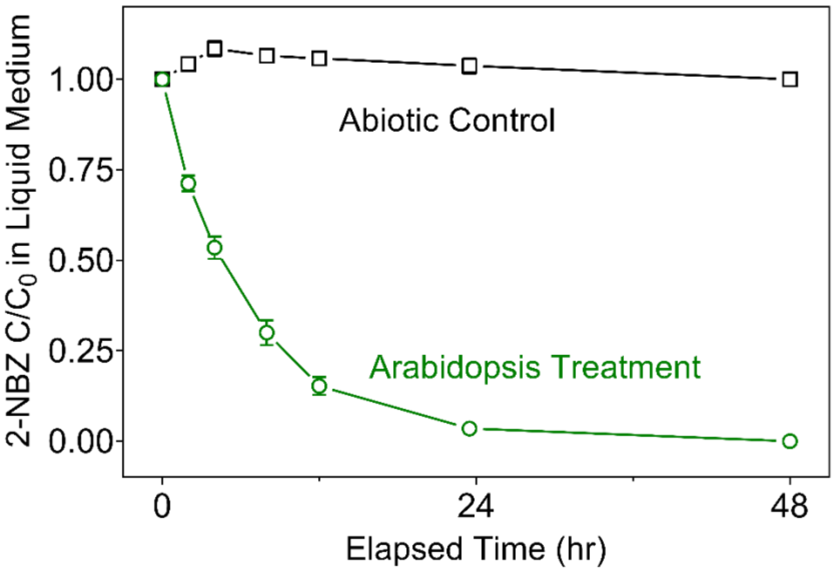

Figure S5: 2N-BZ 48-hour plant uptake data. Nominal C_0 of 20 µg/L. n=3–4, error bars represent standard error and are obscured by symbols for some datapoints.

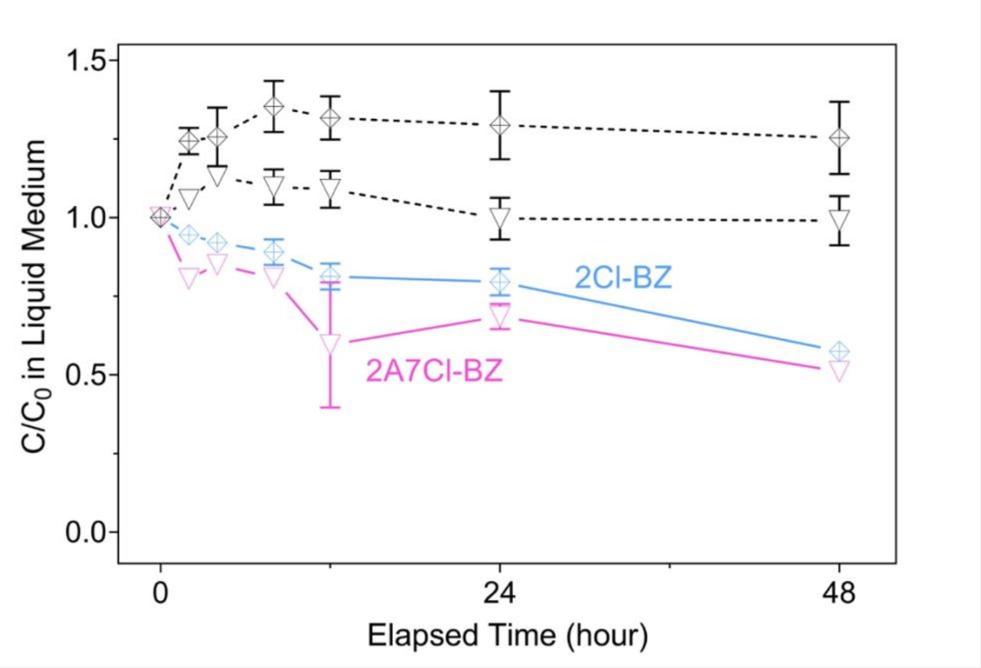

Figure S6: 2A7Cl-BZ and 2Cl-BZ 48-hour plant uptake data. Nominal C_0 of 20 µg/L. n=3–4, error bars represent standard error and are obscured by symbols for some datapoints. Black lines denote no-plant controls matching the symbols of the plant treatments.

Table S4: Sorption results. To test for sorption to plant tissue, 11 or 12-day old unexposed Arabidopsis plants were placed in medium spiked to a nominal 20 µg/L. n=2–3 medium samples were collected from the master mix at t=0 and after 5 minutes of Arabidopsis exposure. A very slight amount of plant uptake may occur during that time, but the majority of removal was attributed to sorption.

Compound	*Percent Removal of Nominal 20 µg/L from Medium After Five Minutes*
1A-BT	2%
2Cl-BZ	3%
1A-BZ	4%
BZ	12%
2A7Cl-BZ	6%
Carb-BZ	0%
2A-BZ	3%
BT	9%
CN-BZ	33%
2N-BZ	1%

Table S5: Uptake rate k values, including abiotic control info, for compounds in this study. Data are taken from Figures 1, S2, S3, S4, and S5. Determination of significant removal used detailed 48-hour data for all Arabidopsis treatments and most negative controls, but for four compounds' negative controls (BZ, 2A-BZ, CN-BZ, and Carb-BZ) only the 10-day data (Figure S3) was available. These compounds are noted with an * below. Second-order curve fits (using least-squares regression) had equal to or higher r^2 values for all compounds than first- or zero-order curve fits except for BZ (first-order $r^2 = 0.97$ vs. second-order $r^2 = 0.95$); 2Cl-BZ ($r^2 = 0.81$ for first-, second-, and zero-order); and 2N-BZ (first-order $r^2 = 0.99$ vs. second-order $r^2 = 0.96$), but the second-order rate values for these two compounds are provided below for comparative purposes. Second-order kinetics have been previously reported for similar hydroponic plant uptake kinetics results.[17–19] Note that rate constants here are unitless because the values are derived from relative concentration (C/C_0) values.

**Compound**	**Treatment**	**Second-order rate (*k_2*), no units since derived from C/C_0 data** (*: See figure caption)	**r^2 for Curve Fit**
1A-BT	Arabidopsis	N/A, no significant removal ($p = 0.16$)	N/A, no significant removal
	Negative (no-plant) control	N/A, no significant removal ($p = 0.39$)	N/A, no significant removal
2Cl-BZ	Arabidopsis	0.014	0.81
	Negative (no-plant) control	N/A, no significant removal ($p = 0.41$)	N/A, no significant removal
1A-BZ	Arabidopsis	0.016	0.90
	Negative (no-plant) control	Removal is significant ($p = 0.05$), but C/C0 is never less than 0.97	N/A
BZ	Arabidopsis	0.016	0.78
	Negative (no-plant) control	N/A, no significant removal ($p = 0.68$)*	N/A, no significant removal
2A7Cl-BZ	Arabidopsis	0.017	0.84
	Negative (no-plant) control	N/A, no significant removal ($p = 0.16$)	N/A, no significant removal
Carb-BZ	Arabidopsis	0.023	0.54
	Negative (no-plant) control	N/A, no significant removal ($p = 0.51$)*	N/A, no significant removal
2A-BZ	Arabidopsis	0.024	0.58
	Negative (no-plant) control	N/A, no significant removal ($p = 0.29$)*	N/A, no significant removal
BT	Arabidopsis	0.165	0.95
	Negative (no-plant) control	Previously established to not experience significant abiotic removal in this plant growth medium[1]	N/A
CN-BZ	Arabidopsis	0.350	0.96

**Compound**	**Treatment**	**Second-order rate (k_2), no units since derived from C/C_0 data** (*: See figure caption)	**r^2 for Curve Fit**
	Negative (no-plant) control	N/A, no significant removal (p = 0.33)*	N/A, no significant removal
2N-BZ	Arabidopsis	0.284	0.96
	Negative (no-plant) control	N/A, no significant removal (p = 0.07)	N/A, no significant removal

Table S6: p-values for comparisons between benzimidazole uptake rates from Figure 1.

Compound Pair for Comparison	Paired t-test Comparison *p* Value (* denotes < 0.05)
1A-BT and BT	0.01*
2Cl-BZ and 1A-BZ	0.33
2Cl-BZ and CN-BZ	0.002*
2Cl-BZ and 2N-BZ	0.004*
2Cl-BZ and Carb-BZ	0.003*
2Cl-BZ and 2A-BZ	0.003*
1A-BZ and 2A7Cl-BZ	0.02*
1A-BZ and BZ	0.006*
2A7Cl-BZ and BZ	0.12
2A7Cl-BZ and 2A-BZ	0.03*
BZ and Carb-BZ	0.004*
BZ and 2A-BZ	0.003*
BZ and BT	0.02*
BZ and CN-BZ	0.003*
BZ and 2N-BZ	0.008*
Carb-BZ and 2A-BZ	0.40
2A-BZ and BT	0.02*
BT and CN-BZ	0.01*
CN-BZ and 2N-BZ	0.37

Table S7: QSAR descriptors as obtained from Spartan'20 parallel suite computations. Descriptors A-AG were obtained directly from Spartan. Each descriptor is accompanied by units or "NA" if no units are applicable, a short description of what each parameter means, and the tab each descriptor can be found in within the Spartan program.

Decriptor label	QSAR Descriptor	Units	Description	Tab
A	Energy (au)	au (arbitrary units)	A molecule's total energy (Hartree-Fock molecular orbital, density functional, Moller Plesset, and Post Hartree-Fock calculations).	molecule
B	E HOMO (eV)	eV	Energy of the highest occupied molecular orbital	molecule
C	Conformers	NA	Number of singly-bonded conformers in a systematic equilibrium conformer or conformer distribution calculation.	molecule
D	Dipole (debye)	debye	Measure of charge separation.	molecule
E	E LUMO (eV)	eV	Energy of the lowest unoccupied molecular orbital.	molecule
F	Molecular Wt. (amu)	amu (atomic mass units)	Molecular weight a molecule	molecule
G	Molecular Mass (amu)	amu (atomic mass units)	Molecular mass of a molecule	molecule
H	CPK Area (Å²)	Å²	Surface area of a molecule	QSAR-CPK model
I	CPK Volume (Å³)	Å³	Volume of a molecule	QSAR-CPK model
J	Acc. Area (Å²)	Å²	Accessible surface area based on the electron density surface.	QSAR-computed electron density
K	Min ElPot (kJ/mol)	kJ/mol	Minimum electrostatic potential at the electron density surface	QSAR-computed electron density
L	Max ElPot (kJ/mol)	kJ/mol	Maximum electrostatic potential at the electron density surface.	QSAR-computed electron density
M	Log P	NA	method	QSAR tab
N	HBD Count	NA	Number of hydrogen bond donors	QSAR tab
O	PSA (Å²)	Å²	Polar surface area of a molecule determined by the summation of the area of nitrogen, oxygen, and hydrogen atoms attached to nitrogen and oxygen.	QSAR-CPK model
P	CPK Ovality	NA	Measurment of the deviation from a spherical shape	QSAR-CPK model
Q	Polar Area(75) (Å²)	Å²	Area of electron density for which the absolute value of the electrostatic potential is above and below 75 kJ/mol in the electrostatic potential map.	QSAR-computed electron density
R	Acc. Polar Area(75) (Å²)	Å²	Accessible area of electron density for which the absolute value of the electrostatic potential is above and below 75 kJ/mol in the electrostatic potential map.	QSAR-computed electron density
S	Min LocIonPot (ev)	eV	Minimum local ionization potential at the electron density surface.	QSAR-computed electron density
T	Polarizability	NA	Empirically estimated polarizability	QSAR tab
U	HBA Count	NA	Number of hydrogen bond acceptors	QSAR tab
V	Property Min(Surface1) kJ	kJ	Minimum displayed electron ionization energy mapped between -250 and 250 kJ and plot with an iso val of 99.92% (0.0002 e-/arbitrary unit volume).	surface 1 properites: electrostatic potential map
W	Property Max(Surface1) kJ	kJ	Maximum displayed electron ionization energy mapped between -250 and 250 kJ and plot with an iso val of 99.92% (0.0002 e-/arbitrary unit volume).	surface 1 properites: electrostatic potential map
X	Surf Area(Surface1)(Å²)	Å²	Surface area in square angstroms	surface 1 properites: electrostatic potential map
Y	Acc. Area(Surface1)(Å²)	Å²	Accessible area on the electron density surface determined.	surface 1 properites: electrostatic potential map
Z	Polar Area(Surface1)(Å²)	Å²	Polar area on the electron density surface determined.	surface 1 properites: electrostatic potential map
AA	Surf Volume(Surface1)(Å³)	Å³	Surface volume in cubic angstroms.	surface 1 properites: electrostatic potential map
AB	Acc. P-Area(Surface1)(Å²)	Å²	Accessible polar area on the electron density surface .	surface 1 properites: electrostatic potential map
AC	Property Min(Surface2) eV	eV	Minimum displayed local ionization potential mapped between 5-15 eV and plot with an iso val of 99.92% (0.0002 e-/arbitrary unit volume).	surface 2 properites local ionization potential map
AD	Property Max(Surface2) eV	eV	Maximum displayed local ionization potential mapped between 5-15 eV and plot with an iso val of 99.92% (0.0002 e-/arbitrary unit volume).	surface 2 properites local ionization potential map
AE	Surf Area(Surface2)(Å²)	Å²	Surface area on the ionization potential determined.	surface 2 properites local ionization potential map
AF	Acc. Area(Surface2)(Å²)	Å²	Accessible area on the ionization potential determined.	surface 2 properites local ionization potential map
AG	Surf Volume(Surface2)(Å³)	Å³	Surface volume in cubic angstroms.	surface 2 properites local ionization potential map

Table S8: QSAR data obtained in Spartan '20 for the fungicides studied. (see also Table S7 for detailed QSAR descriptors)

		Compound										
**QSAR Descriptor (units)**	**Label**	carbendazim	thiabendazole	benzotriazole	1-aminobenzotriazole	1-aminobenimidazole	1-H benzimidazole-2-carbonitrile	2-nitrobenzimidazole	aminobenzimidazole	benzimidazole	2-chlorobenzimidaxole	2-amino-7-chloro-benzimidazole
Energy (au)	**A**	-662.9254	-947.546715	-395.74843	-451.054	-435.057	-471.957	-584.184	-435.106	-379.754	-839.33	-894.688
E HOMO (eV)	**B**	-7.8	-7.83	-8.58	-8.52	-8.2	-8.53	-8.65	-7.47	-8.19	-8.3	-7.64
Conformers	**C**	4	2	1	3	3	1	1	1	1	1	1
Dipole (debye)	**D**	4.55	3.83	5.71	4.16	5.52	6.98	7.13	6.08	5.02	4.97	8.6
E LUMO (eV)	**E**	1.4	0.52	0.63	0.57	1.36	0.11	-1.03	1.8	1.42	1.24	1.52
Molecular Wt. (amu)	**F**	191.191	201.251	119.127	134.142	133.154	143.149	163.137	133.154	118.139	152.584	167.599
Molecular Mass (amu)	**G**	191.069	201.036	119.048	134.059	133.064	143.048	163.038	133.064	118.053	152.014	167.025
CPK Area (Å²)	**H**	208.01	207.35	134.25	150.75	157.16	163.02	168.03	154.91	141.23	156.79	170.54
CPK Volume (Å³)	**I**	184.86	189.77	117	129.06	137.11	144.76	146.56	135.32	125.09	138.44	148.91
Acc. Area (Å²)	**J**	164.2	173.38	119.04	126.83	129.1	138.91	137.35	128.5	121.01	132.75	139.23
Min ElPot (kJ/mol)	**K**	-226.37	-229.52	-255.51	-245.73	-262	-217.17	-246.66	-262.94	-259.33	-244.52	-292.89

		Compound										
**QSAR Descriptor (units)**	**Label**	carbendazim	thiabendazole	benzotriazole	1-aminobenzotriazole	1-aminobenimidazole	1-H benzimidazole-2-carbonitrile	2-nitrobenzimidazole	aminobenzimidazole	benzimidazole	2-chlorobenzimidaxole	2-amino-7-chloro-benzimidazole
Max ElPot (kJ/mol)	**L**	254.8	256.87	339.93	243.27	265.25	382.44	351.38	346.39	340.48	354.43	374.7
Log P	**M**	1.79	3.31	1.95	1.71	NA	2.71	2.33	1.49	1.78	2.48	2.05
HBD Count	**N**	0	0	1	1	1	0	0	0	0	0	0
PSA (Å²)	**O**	48.824	27.583	38.551	55.881	38.047	35.902	60.24	45.6	20.548	20.656	45.531
CPK Ovality	**P**	1.33	1.3	1.16	1.22	1.22	1.22	1.25	1.22	1.17	1.21	1.26
Polar Area(75) (Å²)	**Q**	94.89	79.06	62.79	61.12	73.54	76.47	81.88	87.59	69.88	62.06	109.82
Acc. Polar Area(75) (Å²)	**R**	82.22	65.17	54.32	55.6	60.63	62.69	63.05	73.18	60.4	46.44	88.66
Min LocIonPot (ev)	**S**	11.02	10.99	11.28	11.21	10.87	11.48	11.59	10.7	10.85	11.33	10.93
Polarizability	**T**	54.2	54.8	48.69	49.7	50.24	51.08	51.46	50.16	49.26	50.36	51.29
HBA Count	**U**	2	3	3	4	2	2	4	1	1	1	1
Property Min(Surface1) kJ	**V**	-153.89	-155.01	-186.19	-178.83	-183.79	-151.88	-196.18	-183.66	-179.92	-168.01	-208.27

		Compound										
**QSAR Descriptor (units)**	**Label**	carbendazim	thiabendazole	benzotriazole	1-aminobenzotriazole	1-aminobenimidazole	1-H benzimidazole-2-carbonitrile	2-nitrobenzimidazole	aminobenzimidazole	benzimidazole	2-chlorobenzimidaxole	2-amino-7-chloro-benzimidazole
Property Max(Surface1) kJ	**W**	140.66	150.4	216.44	144.76	169.78	250.05	238.14	233.39	217.33	217	250.28
Surf Area(Surface1)(Å²)	**X**	254.09	257.48	174.33	190.24	196.3	207.47	205.41	195.72	181.21	208.95	221.02
Acc. Area(Surface1)(Å²)	**Y**	231.5	238.73	166.85	176.29	182.06	193.89	186.04	181.04	173.72	195.63	202.47
Polar Area(Surface1)(Å²)	**Z**	26.76	29.31	44.63	39.18	43.33	53.23	52.87	62.72	38.52	32.66	79.78
Surf Volume(Surface 1)(Å³)	**AA**	285.62	299.39	189.58	209.66	219.77	228.26	224.5	216.33	199.62	236.85	254.74
Acc. P-Area(Surface1)(Å²)	**AB**	23.71	23.58	40.13	38.03	35.38	45.35	41.93	53.69	33.36	23.54	67.26
Property Min(Surface2) eV	**AC**	10.77	10.79	11	11.01	10.68	10.98	11.09	10.5	10.66	10.86	10.68
Property Max(Surface2) eV	**AD**	17.7	16.59	17.74	18.32	18.04	17.16	18.36	17.3	17.4	16.91	17.41

		Compound										
**QSAR Descriptor (units)**	**Label**	carbendazim	thiabendazole	benzotriazole	1-aminobenzotriazole	1-aminobenimidazole	1-H benzimidazole-2-carbonitrile	2-nitrobenzimidazole	aminobenzimidazole	benzimidazole	2-chlorobenzimidaxole	2-amino-7-chloro-benzimidazole
Surf Area(Surface2)(Å²)	**AE**	253.81	257.48	174.33	190.24	196.3	207.47	205.41	195.72	181.21	208.95	221.02
Acc. Area(Surface2)(Å²)	**AF**	231.03	238.73	166.85	176.29	182.06	193.89	186.04	181.04	173.72	195.63	202.47
Surf Volume(Surface2)(Å³)	**AG**	285.06	299.39	189.58	209.66	219.77	228.26	224.5	216.33	199.62	236.85	254.74

(a)

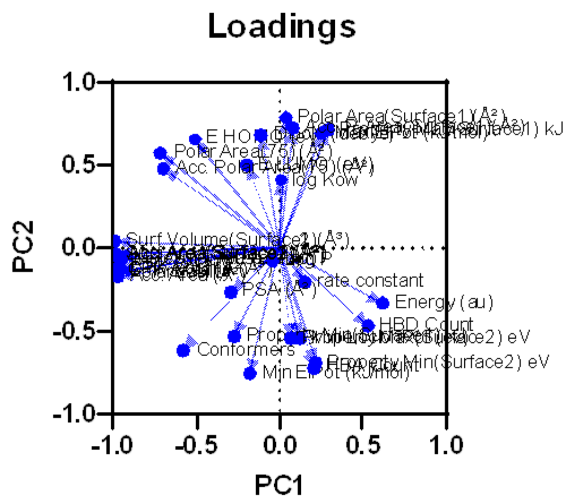

(b)

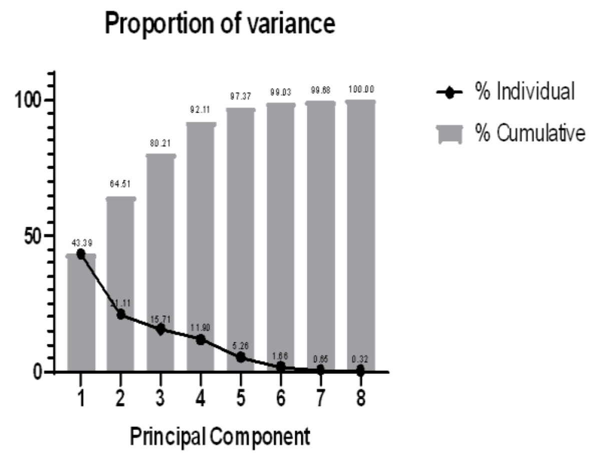

(c)

Var	PC1	PC2	PC3	PC4	PC5	PC6	PC7	PC8
rate constant	0.155	-0.209	0.832	-0.053	0.467	-0.043	0.096	-0.090
log Kow	0.010	0.406	0.450	0.479	-0.629	-0.008	0.072	-0.038
Energy (au)	0.620	-0.337	-0.266	-0.179	0.616	0.057	0.125	-0.021
E HOMO (eV)	-0.508	0.650	-0.522	-0.073	0.114	-0.001	-0.127	-0.110
Conformers	-0.579	-0.622	-0.461	-0.181	-0.048	-0.146	0.047	0.087
Dipole (debye)	-0.110	0.675	0.646	-0.308	0.079	-0.088	0.078	0.016
E LUMO (eV)	-0.196	0.496	-0.767	0.289	-0.068	-0.192	0.015	-0.047
Molecular Wt. (amu)	-0.949	-0.066	0.226	-0.073	-0.174	0.072	-0.028	-0.045
Molecular Mass (amu)	-0.950	-0.071	0.225	-0.076	-0.169	0.074	-0.026	-0.045
CPK Area (Å²)	-0.982	-0.143	0.054	-0.057	0.081	0.051	0.011	-0.014
CPK Volume (Å³)	-0.980	-0.132	0.058	0.006	0.112	0.059	0.041	-0.020
Acc. Area (Å²)	-0.971	-0.178	0.106	0.018	0.106	-0.020	0.041	-0.029
Min ElPot (kJ/mol)	-0.180	-0.761	0.192	0.378	0.440	-0.075	-0.097	-0.008
Max ElPot (kJ/mol)	0.253	0.688	0.600	0.267	0.153	0.041	0.026	-0.079
Log P	-0.041	-0.079	0.871	0.449	-0.044	-0.127	-0.028	0.118
HBD Count	0.532	-0.472	-0.219	-0.360	-0.282	-0.453	0.100	-0.149
PSA (Å²)	-0.291	-0.270	0.146	-0.876	0.039	-0.009	-0.210	-0.083
CPK Ovality	-0.963	-0.121	0.031	-0.221	0.008	0.037	-0.081	0.013
Polar Area(75) (Å²)	-0.715	0.568	0.065	-0.363	0.126	0.009	0.118	0.005
Acc. Polar Area(75) (Å²)	-0.700	0.474	-0.123	-0.431	0.196	-0.071	0.197	0.035
Min LocIonPot (ev)	0.119	-0.547	0.811	0.078	-0.128	-0.046	-0.052	-0.028
Polarizability	-0.968	-0.152	0.122	-0.034	0.125	0.083	0.030	-0.019
HBA Count	0.207	-0.727	0.296	-0.574	-0.101	0.011	-0.022	-0.027
Property Min(Surface1) kJ	-0.273	-0.537	-0.104	0.613	0.440	-0.226	-0.066	0.030
Property Max(Surface1) kJ	0.293	0.708	0.596	0.098	0.192	0.084	-0.023	-0.060
Surf Area(Surface1)(Å²)	-0.994	-0.046	0.065	0.067	-0.018	-0.031	-0.008	-0.006
Acc. Area(Surface1)(Å²)	-0.981	-0.042	0.031	0.167	-0.010	-0.066	0.049	-0.005
Polar Area(Surface1)(Å²)	0.040	0.779	0.316	-0.483	0.089	-0.183	-0.116	0.063
Surf Volume(Surface1)(Å³)	-0.988	0.035	0.044	0.088	-0.093	-0.059	-0.011	0.017
Acc. P-Area(Surface1)(Å²)	0.083	0.719	0.213	-0.569	0.126	-0.276	-0.078	0.094
Property Min(Surface2) eV	0.218	-0.698	0.623	-0.121	-0.203	-0.130	0.066	0.008
Property Max(Surface2) eV	0.068	-0.549	0.057	-0.784	-0.144	0.217	0.074	0.063
Surf Area(Surface2)(Å²)	-0.994	-0.045	0.066	0.067	-0.019	-0.031	-0.009	-0.006
Acc. Area(Surface2)(Å²)	-0.981	-0.040	0.034	0.168	-0.011	-0.067	0.047	-0.004
Surf Volume(Surface2)(Å³)	-0.988	0.037	0.047	0.088	-0.094	-0.059	-0.013	0.018

Figure S7: Principal Component Analysis (PCA) of QSAR descriptors and uptake rate constants. (a) PCA loadings plot projection plotted onto the first and second principal components, illustrating relationships between QSAR data (b) PCA cumulative proportion of variance plot, demonstrating the relative and cumulative variance explained from each principal component in the PCA (c) tabular representation of QSAR variables' values in each of the principal components.

Table S9: Pearson rho correlations and p-values (for descriptors with p <0.1) for the correlation of QSAR parameters to experimental rate constants (for all compounds).

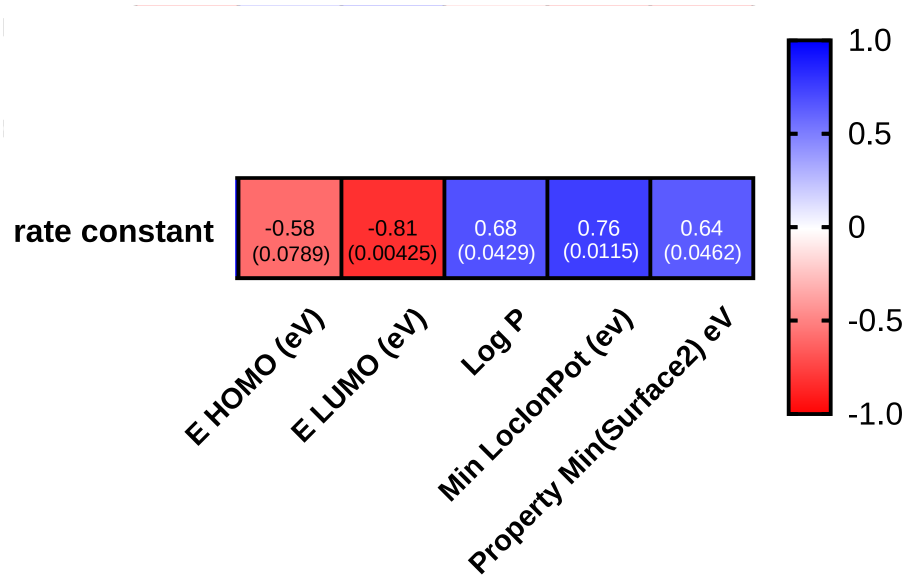

Full details are in Table S7. For the parameters above:
E HOMO is the Energy of the highest occupied molecular orbital (eV)
E LUMO is the Energy of the lowest unoccupied molecular orbital (eV)
Log P is the partitioning coeffient
Min LocIonPot is the Minimum local ionization potential at the electron density surface (eV)
Property Min(Surface2) is Minimum displayed local ionization potential mapped between 6-15 eV

PLANT METABOLOMICS SETTINGS

Metabolomics via high-resolution mass spectrometry (*overall summary*)

Extracted plant tissues were analyzed on a Thermo Q-Exactive High-Resolution Mass Spectrometer, run in MS as well as both positive and negative data-dependent MS/MS modes. The chromatography and method parameters from the Agilent Triple Quadrupole MS BZ method (Table S3) was used on the Q-Exactive for these samples. Q-Exactive data of the metabolomics samples were analyzed via Compound Discoverer 3.1. The workflow “Untargeted Metabolomics with Statistics Detect Unknowns with ID using Online Databases and mzLogic” was used. The results were filtered to remove background with the “background is false” filter, and to those compounds with a p-value ≤0.05 in the fold-change ratio (the ratio of the peak area of the treatment plant tissue extracts to the peak area in the positive control plant tissue extracts) for the compound of interest. Filtered results were then sorted from greatest to least peak area ratio. Results with a peak area ratio ≥100 were selected for further analysis. Those compounds were then sorted by retention time, and similar retention time compounds (within 0.07 min) were grouped as likely in-source fragments. The compound was listed as a proposed structure if the accurate mass deviation between the proposed compound and measured m/z was <10ppm.

Compound Discoverer Analysis

Compound Discoverer (Thermo Scientific) analysis was run April 7, 2021 (Compound Discoverer version used not noted) using the .RAW files produced by the Q-Exactive on September 23, 2020. Run as samples were the three carbendazim-exposed plant tissue extract sample MS scan files, the seven benzimidazole-exposed plant tissue extract sample MS scan files, the seven 2-cyanobenzimidazole-exposed plant tissue extract sample MS scan files, and seven unexposed plant tissue extract sample MS scan files. Polarity switching was used for the MS scan (i.e., both positive and negative modes were run in the same sample run). Run as Identification only were composited samples with 100 to 200 uL from each replicate, e.g. 100 uL from each carbendazim replicate for the carbendazim composited sample. The composited samples were run in polarity switching MS as well as both (run separately) negative and positive ddMS2. Composited sample results were used for identification only in Compound Discoverer. Additionally, seven blanks from throughout the run were input to Compound Discoverer as blanks. The established workflow within Compound Discoverer, "Untargeted Metabolomics with Statistics Detect Unknowns with ID using Online Databases and mzLogic" was used. An image of the workflow tree is shown in Figure S8. All workflow settings are listed below. mzCloud, Metabolika, and ChemSpider searches were based on online databases and were completed using the versions available on April 7, 2021.

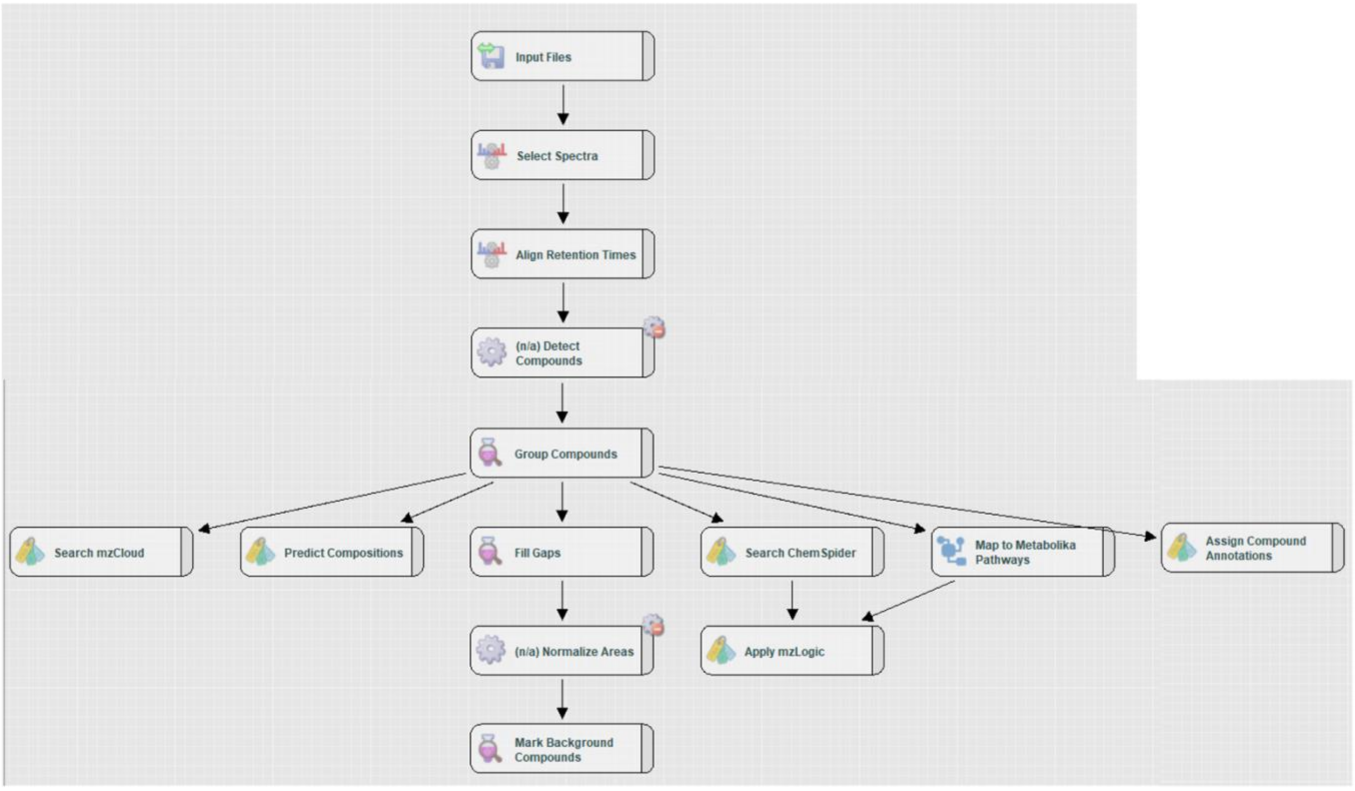

Figure S8: Workflow tree showing the components of the Compound Discoverer automated analysis (screenshot).

Note the **gear symbol with a minus sign on "Detect Compounds" and "Normalize Areas" indicates obsolete nodes as of February 9, 2023 when the screenshot was taken, but there were functional on April 7, 2021 when the analysis was run.**

Workflow node details:

- Select Spectra
    - Lower RT Limit: 0 (if set to 0, the lowest available retention time is used)
    - Upper RT Limit: 0 (if set to 0, the highest available retention time is used)
    - Polarity mode: Any
- Align Retention Times
    - Alignment model: Adaptive curve
    - Maximum Shift (min): 2
    - Mass Tolerance: 5 ppm
- Detect Compounds
    - Ions: [2M+ACN+H]+1; [2M+ACN+Na]+1; [2M+FA-H]-1; [2M+H]+1; [2M+K]+1; [2M+Na]+1; [2M+NH4]+1; [2M-H]-1; [2M-H+HAc]-1; [M+2H]+2; [M+3H]+3; [M+ACN+2H]+2; [M+ACN+H]+1; [M+ACN+Na]+1; [M+Cl]-1; [M+DMSO+H]+1; [M+FA-H]-1; [M+H]+1; [M+H+K]+2; [M+H+MeOH]+1; [M+H+Na]+2; [M+H+NH4]+2; [M+HH2O]+1; [M+H-NH3]+1; [M+K]+1; [M+Na]+1; [M+NH4]+1; [M-2H]-2; [M-2H+K]-1; [M-H]-1; [M-H+HAc]-1; [M-H+TFA]-1; [M-H-H2O]-1
- Group Compounds
    - Mass Tolerance: 5 ppm
    - RT Tolerance [min]: 0.2 ppm
    - Align Peaks: False
    - Preferred Ions: [M+H]+1; [M-H]-1
    - Area Integration: Most Common Ion
    - Peak Rating Contributions:
        - Area Contribution: 3
        - CV Contribution: 10

        - FWHM to Base Contribution: 5
        - Jaggedness Contribution: 5
        - Modality Contribution: 5
        - Zig-Zag Index Contribution: 5
    - Peak Rating Filter
        - Peak Rating Threshold: 0
        - Number of Files: 0
- Fill Gaps
    - Mass Tolerance: 5 ppm
    - S/N Threshold: 1.5
- Normalize Areas
    - Max. QC Area RSD [%]: 30
    - Min. QC Coverage [%]: 50
    - Exclude Blanks: True
    - Normalization Type: [None]
- Mark Background Compounds
    - Max. Sample/Blank: 5
    - Max Blank/Sample: 0
    - Hide Background: True
- Search mzCloud
    - Compound Classes: All
    - Library: Autoprocessed; Reference
    - Search MSn Tree: False
    - DDA Search
        - Identity Search: Cosine
        - Match Activation Type: True
        - Match Activation Energy: Match with Tolerance
        - Activation Energy Tolerance: 20
        - Apply Intensity Threshold: True
        - Similarity Search: Confidence Forward
        - Match Factor Threshold: 60

  - DIA Search
    - Use DIA Scans for Search: False
    - Max. Isolation Width [Da]: 500
    - Match Activation Type: False
    - Match Activation Energy: Any
    - Activation Energy Tolerance: 100
    - Apply Intensity Threshold: False
    - Match Factor Threshold: 20
- Predict Compositions
  - Prediction Settings
    - Mass Tolerance: 5 ppm
    - Min. Element Counts: C H
    - Max. Element Counts: C90 H190 Br3 Cl4 N10 O18 P3 S5
    - Min. RDBE: 0
    - Max. RDBE: 40
    - Min. H/C: 0.1
    - Max. H/C: 4
    - Max. # Candidates: 10
  - Pattern Matching
    - Intensity Tolerance [%]: 30
    - Intensity Threshold [%]: 0.1
    - S/N Threshold: 3
    - Use Dynamic Recalibration: True
  - Fragments Matching
    - Use Fragments Matching: True
    - Mass Tolerance: 5 ppm
    - S/N Threshold: 3
- Search Chem Spider
  - Database(s): BioCyc; Human Metabolome Database; KEGG
  - Search Mode: By Formula or Mass
  - Mass Tolerance: 5 ppm

    - Max. # of results per compound: 100
    - Max. # of Predicted Compositions to be searched per Compound: 3
- Apply mzLogic
    - Max. # of Compounds: 0
    - Max. # mzCloud Similarity Results to consider per Compound: 10
    - Match Factor Threshold: 30
- Map to Metabolika Pathways
    - Search Mode: By Formula or Mass
    - By Mass Search Settings: Mass Tolerance: 5 ppm
    - By Formula Search Settings: Max. # of Predicted Compositions to be searched per Compound: 3
    - Display Settings: Max. # Pathways in ‘Pathways’ column: 20
- Assign Compound Annotations
    - General Settings: Mass Tolerance: 5 ppm
    - Data Sources
        - Data Source #1: mzCloud Search
        - Data Source #2: Predicted Compositions
        - Data Source #3: MassList Search
        - Data Source #4: ChemSpider Search
        - Data Source #5: Metabolika Search
    - Scoring Rules
        - Use mzLogic: True
        - Use Spectral Distance: True
        - SFit Threshold: 20
        - SFit Range: 20
    - Reprocessing: Clear Names: False

VOLCANO PLOTS OF METABOLITE RESULTS

<table>
<tr><th></th><th>Log 0 Fold Change</th><th>Log 1 Fold Change</th></tr>
<tr><td>BZ</td><td>(a)
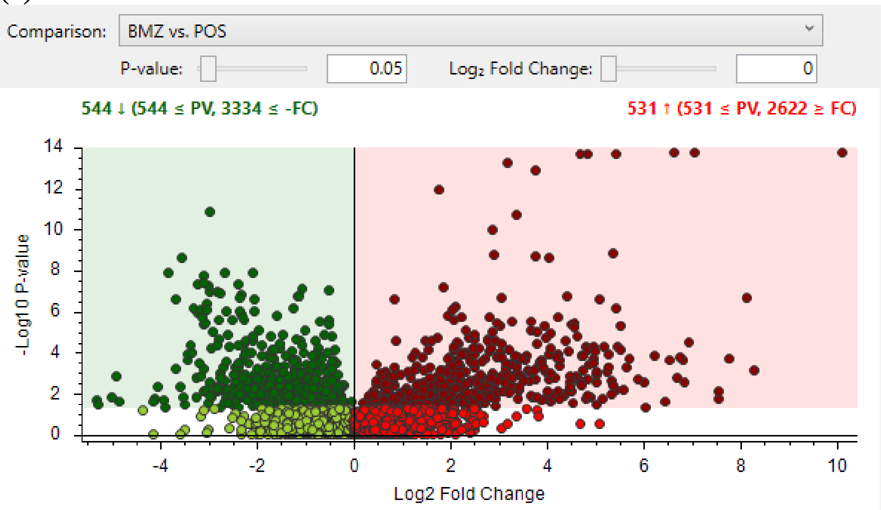
</td><td>(b)
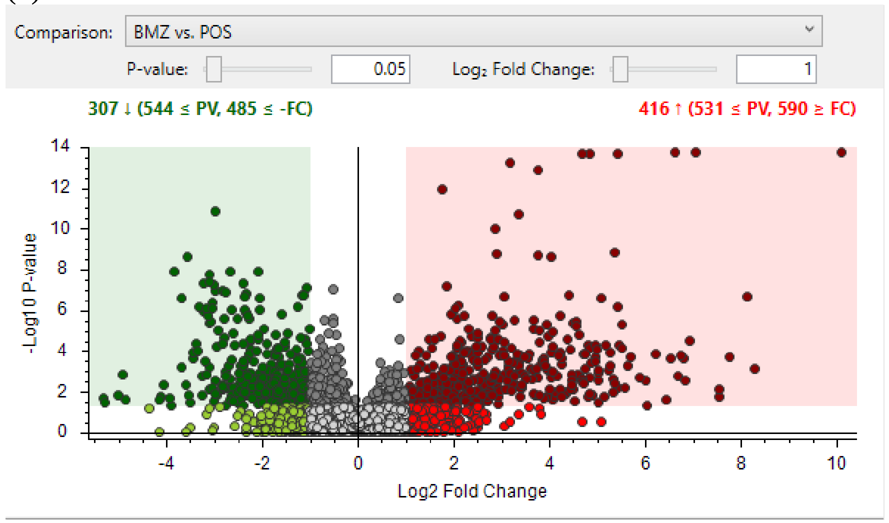
</td></tr>
<tr><td>CN-BZ</td><td>(c)
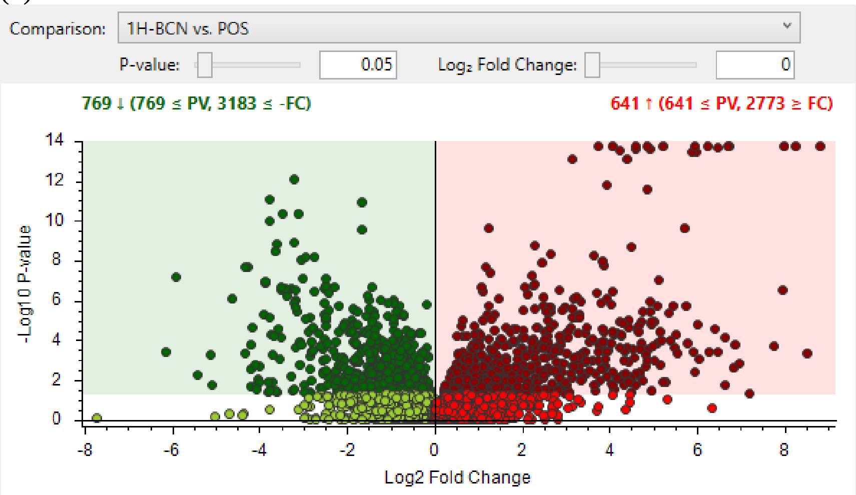
</td><td>(d)
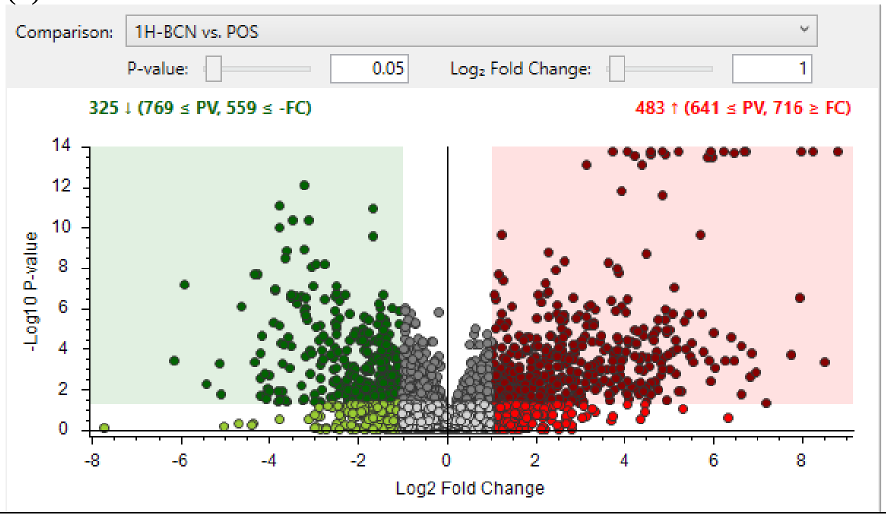
</td></tr>
</table>

CDM	(e) 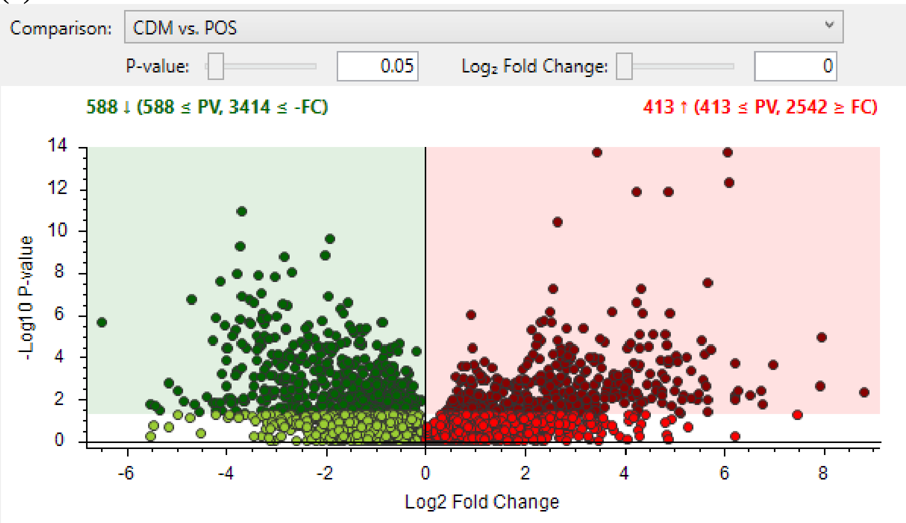	(f) 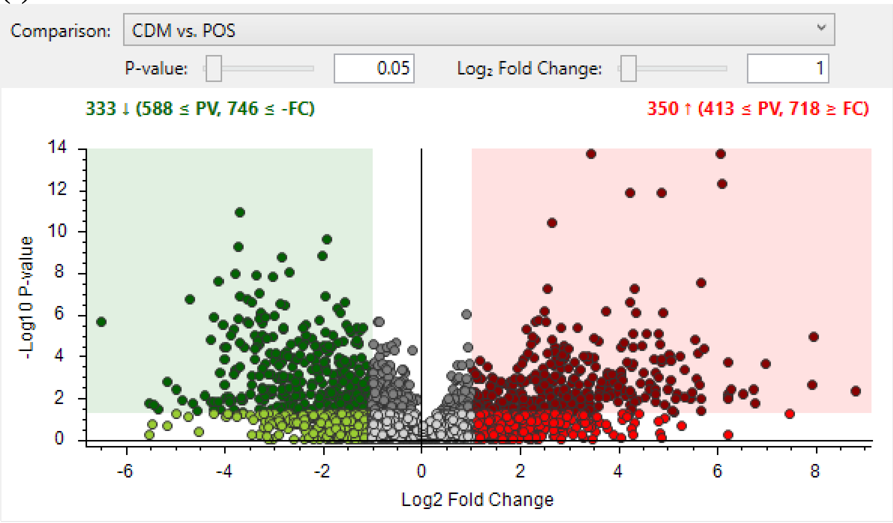

Figure S9: Volcano plots of features changed in plant tissue exposed to BZ (a and b), CN-BZ (c and d), or CDM (e and f). Plant extract metabolites with a p-value of ≤0.05 in the fold-change ratio between exposed plants and unexposed plants and (a, c, e) a log2 fold change >0 are indicated in the red-shaded area (increased in exposed plants) or green-shaded area (reduced in exposed plants) and (b, d, f) a log2 fold change > |1|, indicating a two-fold or greater change between the treatments. (a, c, and e) are meant to illustrate the number of features overall affected by fungicide exposure: BZ: 544 upregulated, 531 downregulated, CN-BZ: 641 upregulated, 769 downregulated, CDM: 413 upregulated, 588 downregulated. (b) illustrates the number of features changed at least two-fold by fungicide exposure: BZ: 307 upregulated, 416 downregulated, CN-BZ: 483 upregulated, 325 downregulated, CDM: 350 upregulated, 333 downregulated. A much more restrictive 100-fold or greater change cutoff along with p-value of ≤0.05 was used for metabolite analysis in this paper. Features decreased in peak area in fungicide-exposed vs. unexposed plants were not examined further in this work, but HRMS data for these features is available upon request.

METABOLITE DETAILS

*ppm[68] = ((difference between measured m/z and exact mass of proposed ionized formula, in atomic mass units)/exact mass of proposed ionized formula)x10^6

Table S10: Metabolites Shared Between All Three Fungicides (Benzimidazole, Carbendazim, and CN-BZ)

									Fragments (Shaded = MS Data)	
Compound Name (Linked to Spectra Below)	Proposed Metabolite Structure (Unionized)	Proposed Metabolite Formula (Unionized)	Confidence Level[69]	Retention Time (min)	Fold Change: (peak area in exposed plants)/(peak area in unexposed plants)	Measured m/z, Positive/ Negative Ion	Exact Mass of Proposed Ionized Formula	Accurate Mass Deviation (ppm)*	Fragment Measured m/z	Proposed Unionized Fragment Molecular Formula
Gamma-glutamyl phosphate with Asp	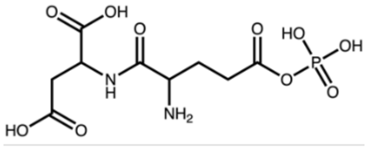	$C_9H_{15}N_2O_{10}P$	Level 3: MS, MS^2, experimental data	8.61	CN-BZ: 114<br>BZ: 104<br>CDM: 109	341.03903 $[M-H]^{-1}$	341.03915	0.4	128.03406	$C_5H_7NO_3$
									96.96821	H_3O_4P
									78.95763	HO_3P^{2-}
Unknown M209	N/A	N/A	Level 5: Accurate Mass of Interest	8.97	CN-BZ: 247<br>BZ: 281<br>CDM: 248	209.06610 $[M-H]^{-1}$	N/A	N/A	128.03407	N/A
Unknown M275	N/A	N/A	Level 5: Accurate Mass of Interest	10.17	CN-BZ: 218<br>BZ: 216<br>CDM: 242	275.02179 $[M-H]^{-1}$	N/A	N/A	274.02530	N/A
									128.03406	N/A
									133.01300	N/A
									115.00240	N/A

Table S11: Metabolites Shared by Benzimidazole and CN-BZ

									Fragments (Shaded = MS Data)	
Compound Name (Linked to Spectra Below)	Proposed Structure (Unionized)	Proposed Formula (Unionized)	Confidence Level[69]	Retention Time (min)	Fold Change: (peak area in exposed plants)/(peak area in unexposed plants)	Measured m/z, Positive/ Negative Ion	Exact Mass of Proposed Ionized Formula	Accurate Mass Deviation (ppm)*	Fragment Measured m/z	Proposed Unionized Fragment Molecular Formula
Unknown M412	N/A	N/A	Level 5: Accurate Mass of Interest	6.31	BZ: 122<br>CN-BZ: 101	412.87012 $[M-H]^{-1}$	N/A	N/A	245.91798	N/A
									128.03410	N/A
									278.87228	N/A
									78.95766	N/A
Unknown M427	N/A	N/A	Level 5: Accurate Mass of Interest	8.02	BZ: 189<br>CN-BZ: 147	427.06427 $[M-H]^{-1}$	N/A	N/A	194.04842	N/A
									128.03404	N/A
									79.95603	N/A
									194.04854	N/A
Unknown M437	N/A	N/A	Level 5: Accurate Mass of Interest	8.10	BZ: 116<br>CN-BZ: 127	437.13818 $[M-H]^{-1}$	N/A	N/A	194.04851	N/A
									128.03412	N/A
									292.02625	N/A
									194.04854	N/A
									79.95594	N/A
L-gamma-glutamyl phosphate	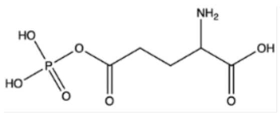	$C_5H_{10}NO_7P$	Level 2a: MS, MS^2, Metabolika and ChemSpider matches	9.75	BZ: 186<br>CN-BZ: 100	226.01173 $[M-H]^{-1}$	226.01221	2	128.03403	$C_5H_7NO_3$
									78.95763	HO_3P^{2-}
									96.96826	H_3O_4P

Table S12: Benzimidazole-Only Metabolites

Compound Abbreviation (Linked to Spectra Below)	Compound Name	Proposed Structure (Unionized)	Proposed Formula (Unionized)	Confidence Level[69]	Retention Time (min)	Fold Change: (peak area in exposed plants)/(peak area in unexposed plants)	Measured m/z, Positive/ Negative Ion	Exact Mass of Proposed Ionized Formula	Accurate Mass Deviation (ppm)*	Fragments (Shaded = MS Data): Fragment Measured m/z	Fragments (Shaded = MS Data): Proposed Unionized Fragment Molecular Formula
Unknown M297	N/A	N/A	N/A^{-}	Level 5: Accurate Mass of Interest	10.47	134	297.10764 $[M+H]^{+1}$	N/A	N/A	130.04982	N/A
										84.04491	N/A
										135.05521	N/A
										297.10770	N/A
BZ	Benzimidazole (parent compound)	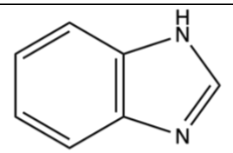	$C_7H_6N_2$	Level 1: MS, MS^2, retention time, reference standard	11.54	1,093	119.06052 $[M+H]^{+1}$	119.06092	3	119.06049	$C_7H_6N_2$
										92.04988	$C_6H_5N^{-}$

Table S13: CN-BZ-Only Metabolites

Compound Name	Proposed Structure (Unionized)	Proposed Formula (Unionized)	Confidence Level[69]	Retention Time (min)	Fold Change: (peak area in exposed plants)/(peak area in unexposed plants)	Measured m/z, Positive/ Negative Ion	Exact Mass of Proposed Ionized Formula	Accurate Mass Deviation (ppm)*	Fragments (Shaded = MS Data): Fragment Measured m/z	Fragments (Shaded = MS Data): Proposed Unionized Fragment Molecular Formula
Unknown M179	N/A	N/A	Level 5: Accurate Mass of Interest	11.50	451	179.04468 $[M+H]^{+1}$	N/A	N/A	161.04425	N/A
									132.10181	N/A
									130.04988	N/A
									86.09690	N/A
									133.04938	N/A
									161.04449	N/A

									Fragments (Shaded = MS Data)	
Compound Name	Proposed Structure (Unionized)	Proposed Formula (Unionized)	Confidence Level[69]	Retention Time (min)	Fold Change: (peak area in exposed plants)/(peak area in unexposed plants)	Measured m/z, Positive/ Negative Ion	Exact Mass of Proposed Ionized Formula	Accurate Mass Deviation (ppm)*	Fragment Measured m/z	Proposed Unionized Fragment Molecular Formula
									179.04495	N/A
Cyano-hydrolyzed CN-BZ	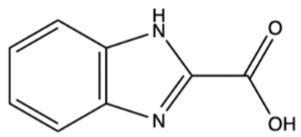	$C_8H_6N_2O_2$	Level 3: MS^2, exp. data	11.78	255	163.05005 $[M+H]^{+1}$	163.05075	4	163.04999	$C_8H_6N_2O_2$
CN-BZ conjugated with N-acetylcysteine	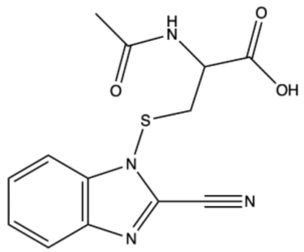	$C_{13}H_{12}N_4O_3S$	Level 2b: MS, MS^2, exp. data	12.36	307	305.06979 $[M+H]^{+1}$	305.07084	3	130.05079	$C_5H_7NO_3^{2-}$
									207.06496	$C_{10}H_{10}N_2OS$
									202.04312	$C_6H_7N_3O_3S^{4-}$
									305.06989	$C_{13}H_{12}N_4O_3S$
Benzimidazole acetyl alanine	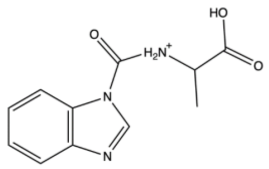	$C_{11}H_{12}N_3O_3^+$	Level 3: MS, MS fragment, exp. data	14.60	107	235.09727 $[M+H]^{+1}$	235.09514	9	130.04980	$C_4H_5N_2O_3^{3-}$
									234.09773	$C_{11}H_{11}N_3O_3^+$
									(No MS2 Data)	

Table S14: CDM-Only Metabolite

Compound Name	Proposed Structure (Unionized)	Proposed Formula (Unionized)	Confidence Level[69]	Retention Time (min)	Fold Change: (peak area in exposed plants)/(peak area in unexposed plants)	Measured m/z, Positive/ Negative Ion	Exact Mass of Proposed Formula	Accurate Mass Deviation (ppm)*	Fragments (Shaded = MS Data): Fragment Measured m/z	Fragments (Shaded = MS Data): Proposed Fragment Molecular Formula
Unknown M780	N/A	N/A	Level 5: Accurate mass of interest	13.21	126	780.33734 $[M-H]^{-1}$	N/A	N/A	128.03404	N/A
									78.95762	N/A
									110.98400	N/A
									277.21680	N/A

Metabolite Mass Spectra and Structures

Metabolites in each category are arranged from smallest to largest retention time.

(NOTE: all proposed metabolites and fragment structures are drawn unionized. Exact masses of proposed ionized formulas presented in the tables above correspond to the ionization state given in the numbers presented in the spectra—generally plus or minus a proton from the structure drawn below).

Metabolites Shared Between All Three Fungicides

Gamma-glutamyl phosphate with Asp

See Purwaha et al. 2015[20] for evidence of the cyclic fragment shown below.

(a) MS1

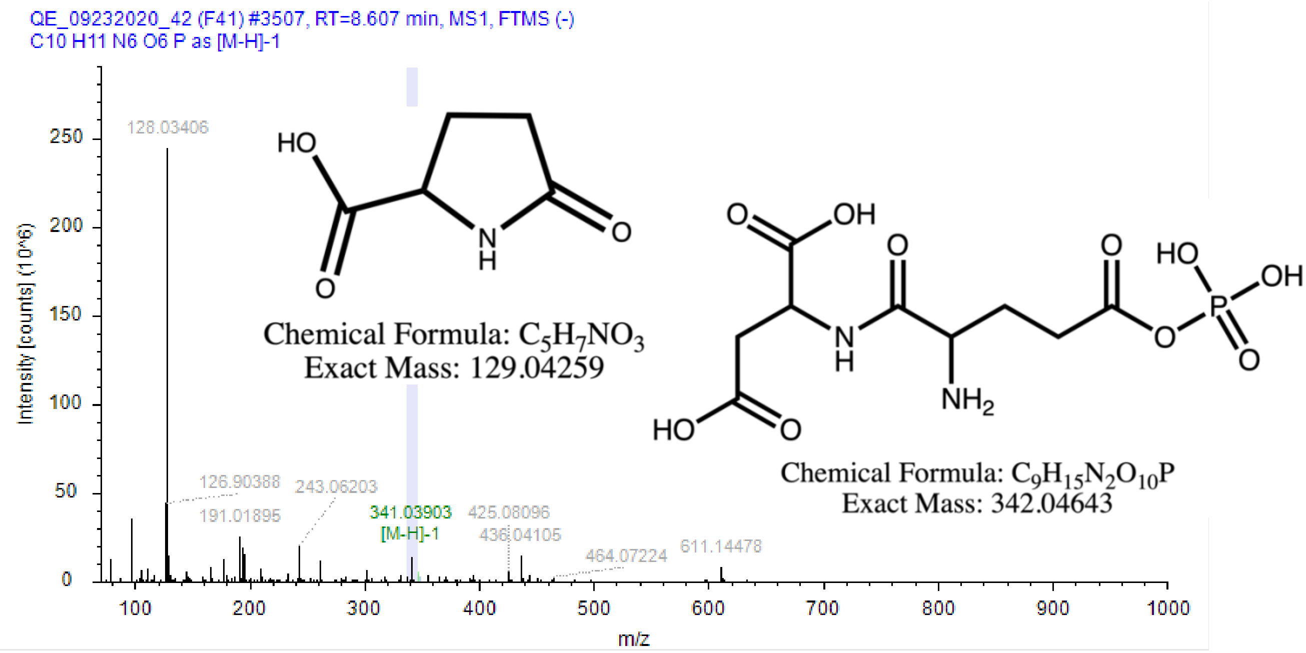

(b) MS2

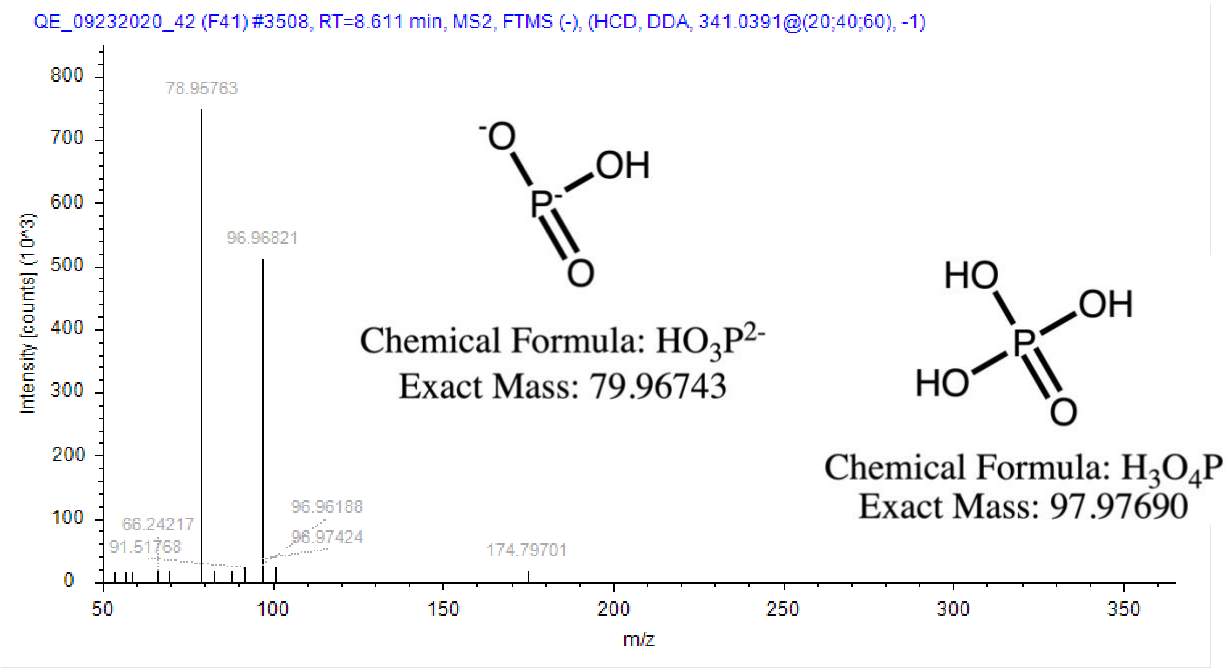

Figure S10: Mass Spectra (a: MS1, b: MS2) and proposed structures for gamma-glutamyl phosphate with Asp

(Link to Summary Table)

<u>Unknown M209</u>

MS1:

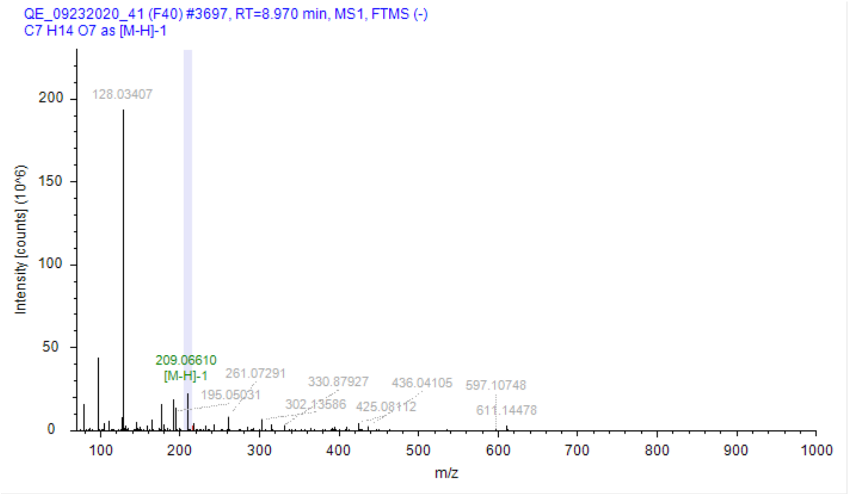

Figure S11: Mass spectra (MS1 only) for unknown M209

(Link to Summary Table)

Unknown M275

(a) MS1:

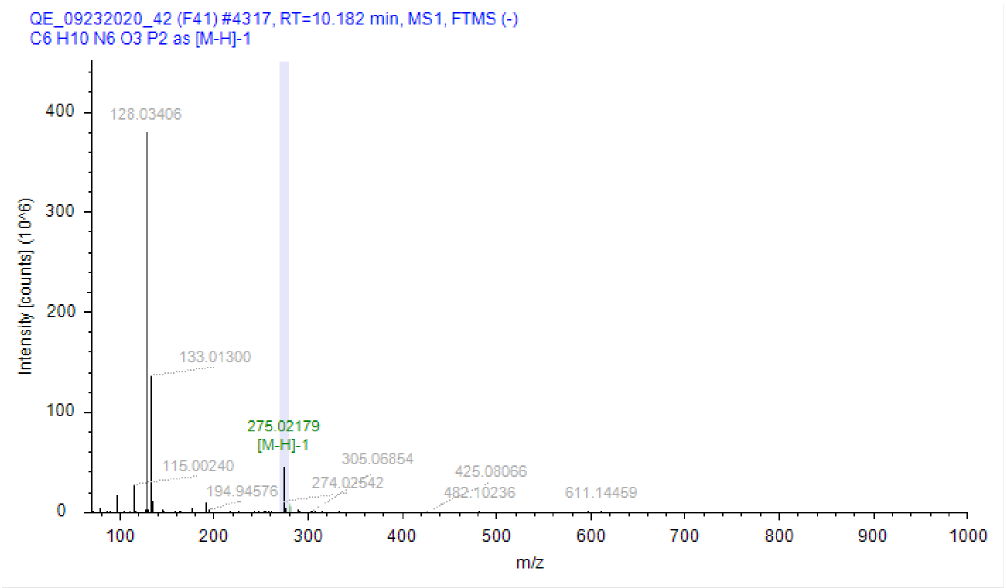

(b) MS1:

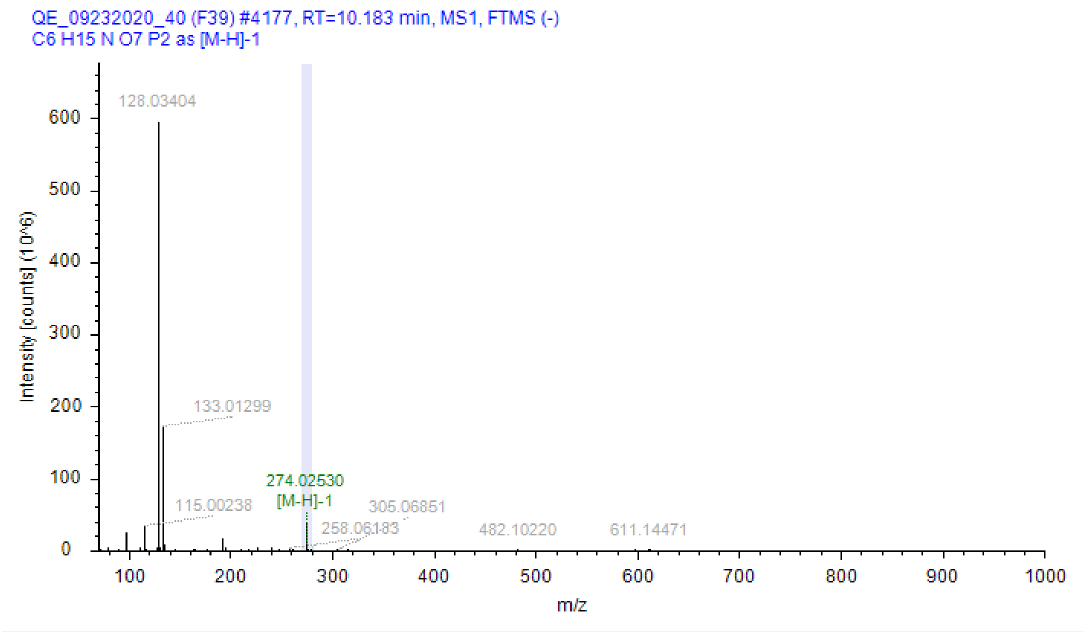

(c) MS2:

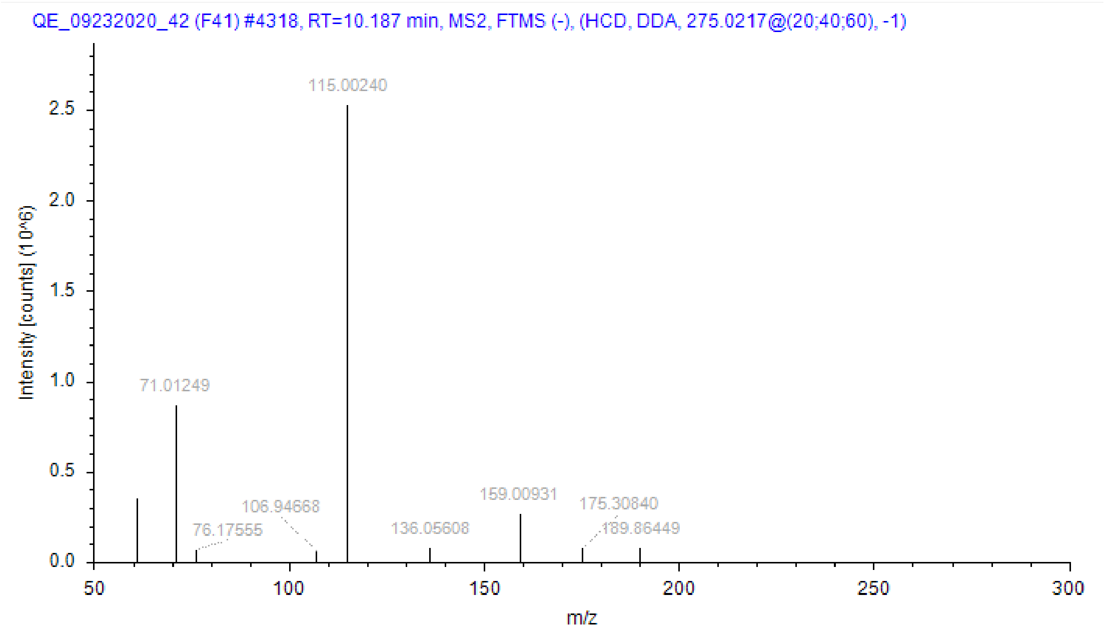

Figure S12: Mass Spectra (a and b: MS1, c: MS2) and proposed structures for unknown M275

(Link to Summary Table)

Metabolites Shared Between Benzimidazole and CN-BZ

Unknown M412

(a) MS1

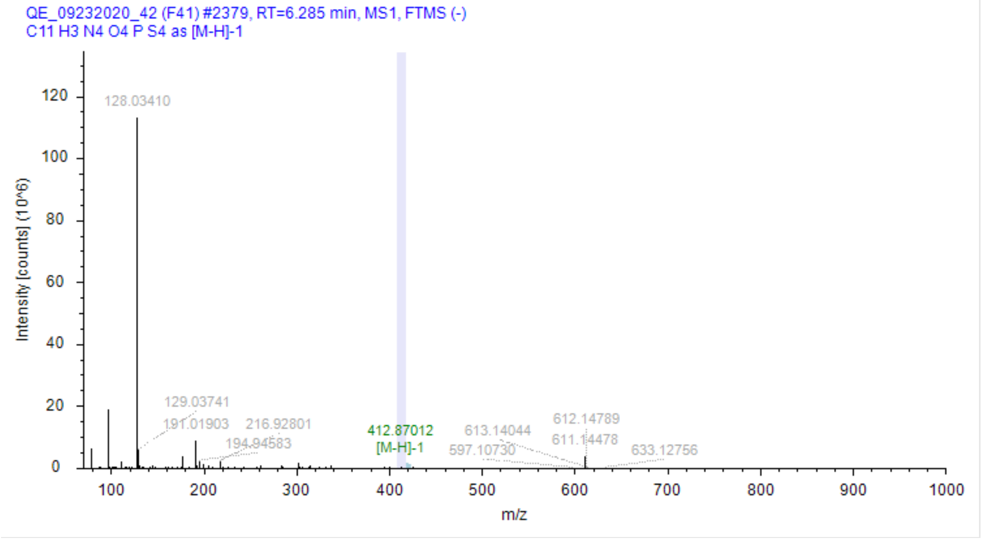

(b) MS2

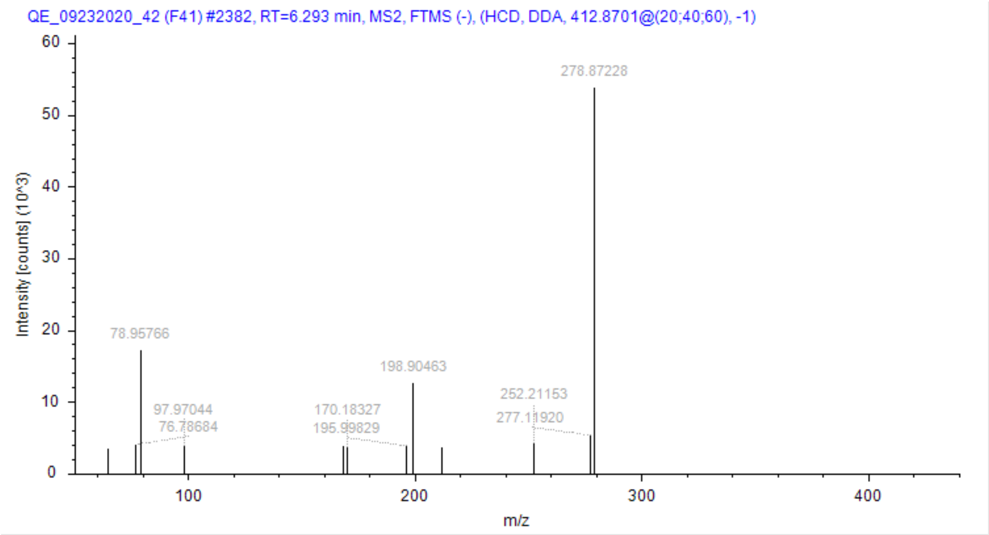

Figure S13: Mass Spectra (a: MS1 b: MS2) and proposed structures for unknown M412

(Link to Summary Table)

Unknown M427

(a) MS1

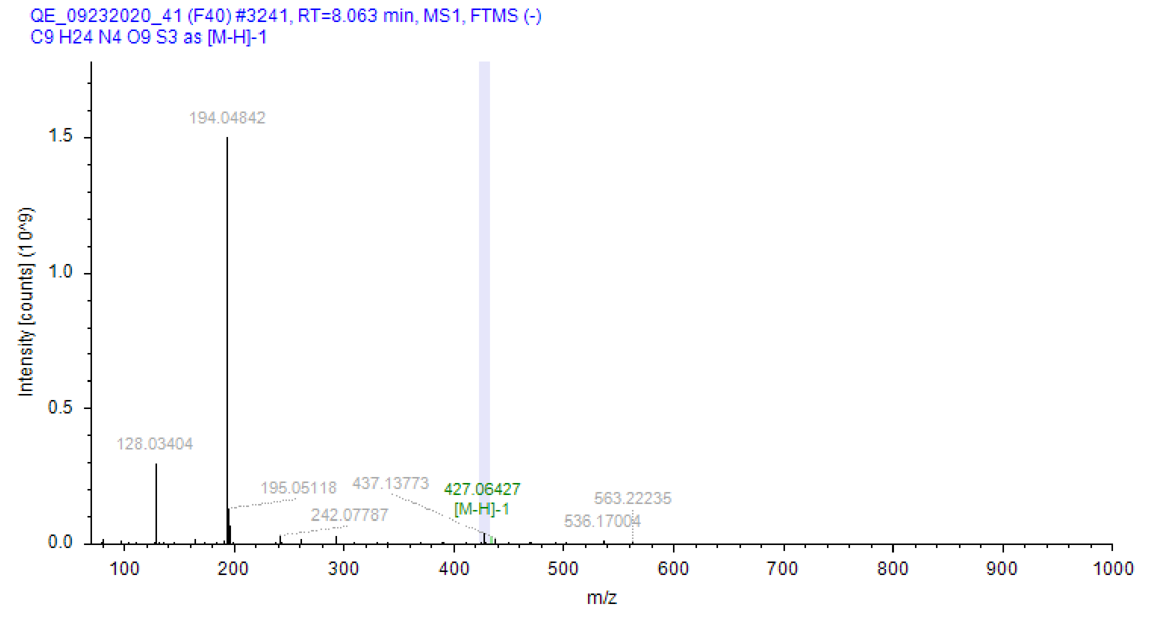

(b) MS2

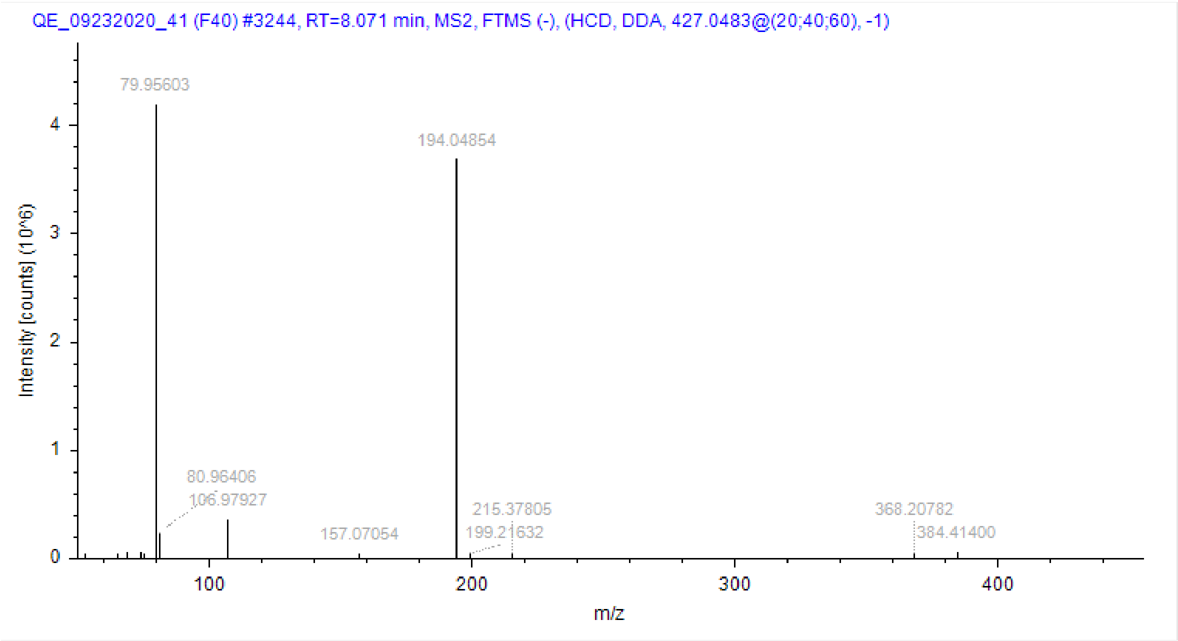

Figure S14: Mass Spectra (a: MS1 b: MS2) and proposed structures for unknown M427

(Link to Summary Table)

Unknown M437

(a) MS1:

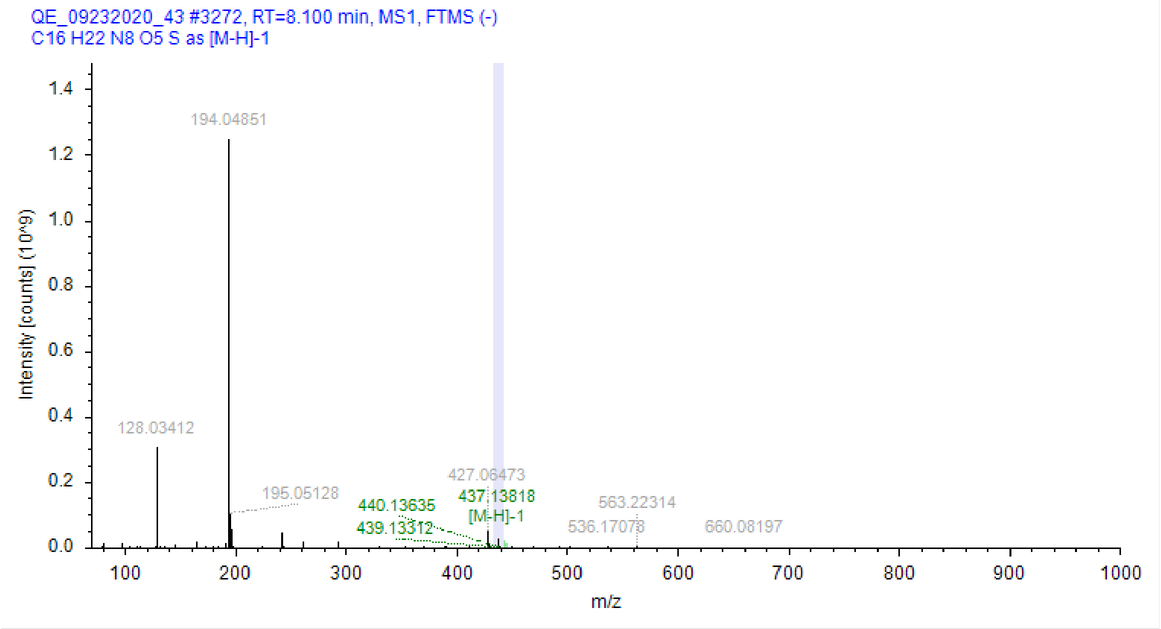

(b) MS2:

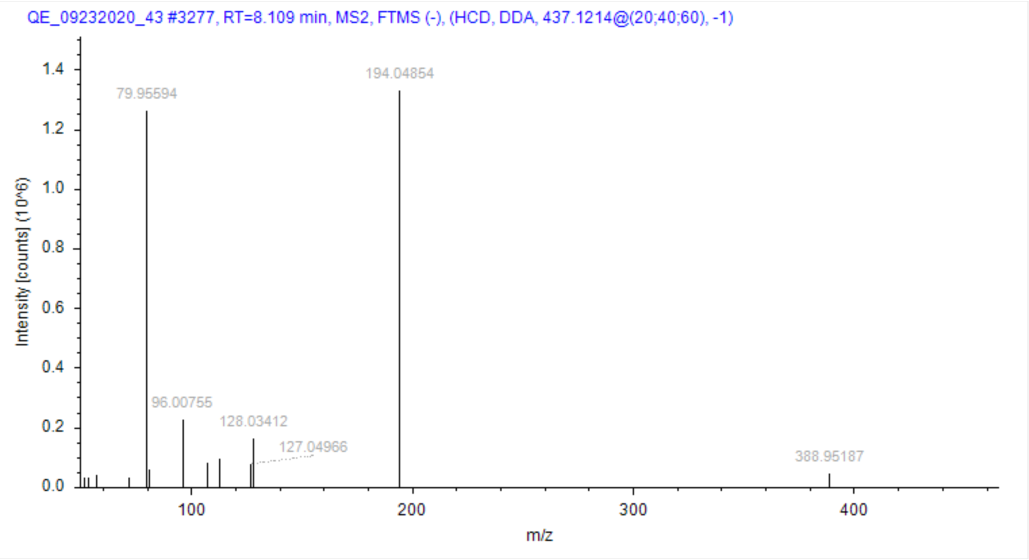

(c) MS1: Additional peak at Accurate Mass 293.03345:

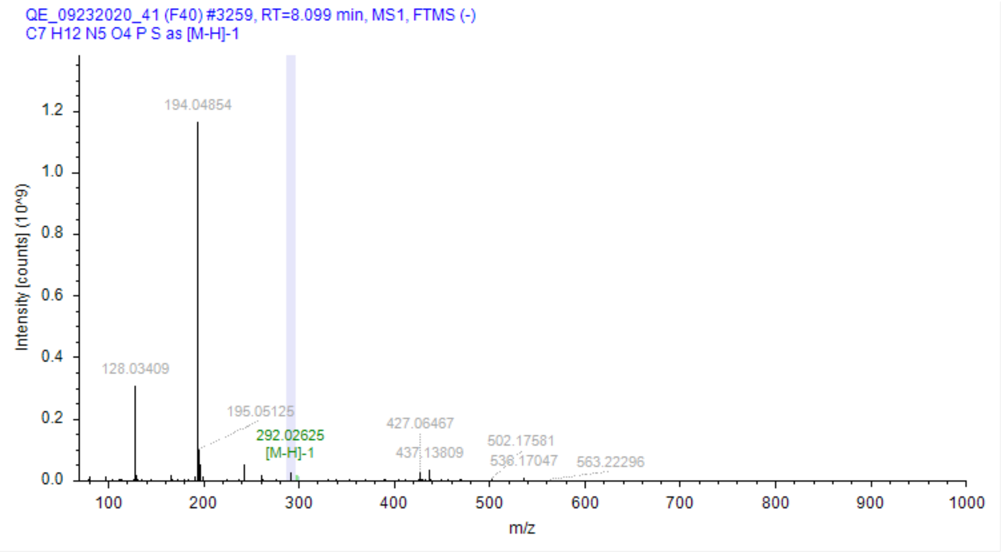

Figure S15: Mass Spectra (a: MS1 b: MS2 c: Additional MS1 peak) and proposed structures for unknown M437

(Link to Summary Table)

L-gamma-glutamyl phosphate

See Purwaha et al. 2015[20] for evidence of the cyclic fragment shown below.

(a) MS1

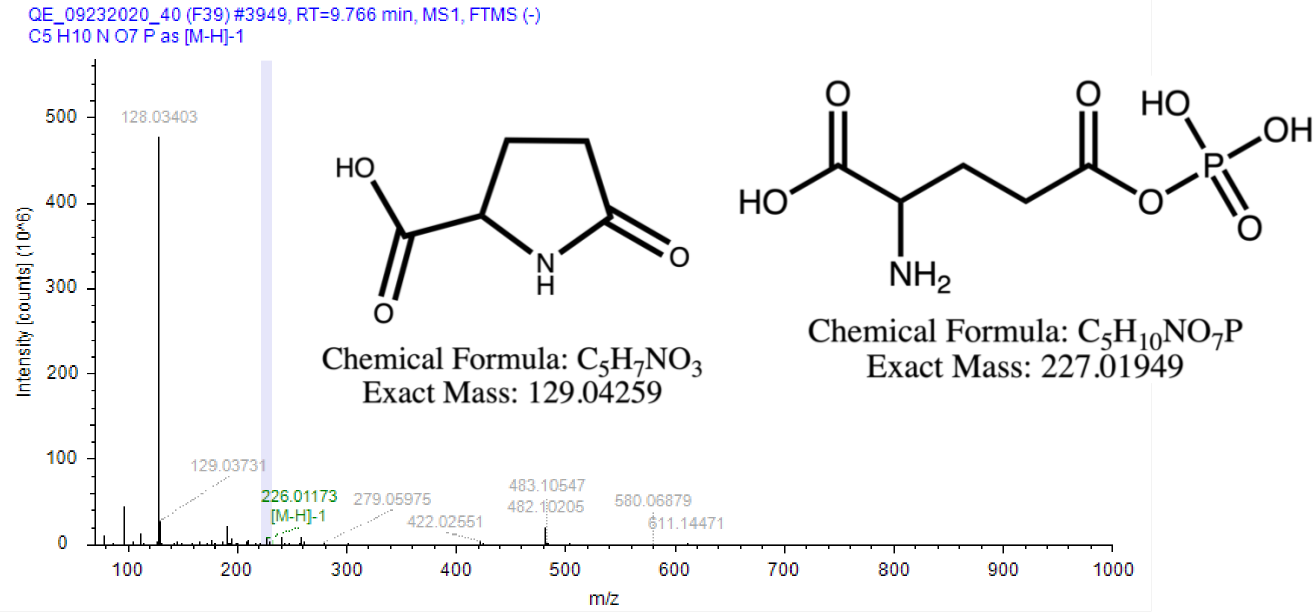

(b) MS2

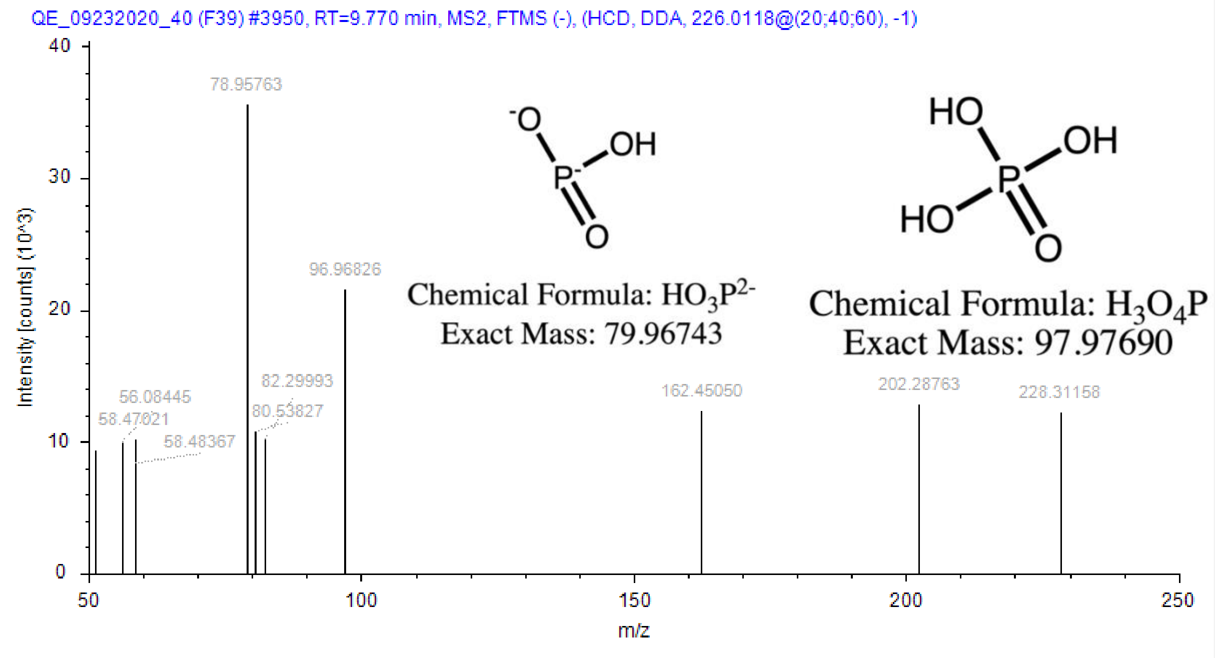

Figure S16: Mass Spectra (a: MS1 b: MS2) and proposed structures for L-gamma-glutamyl phosphate

(Link to Summary Table)

Benzimidazole-Only Metabolites

Unknown M297

(a) MS1

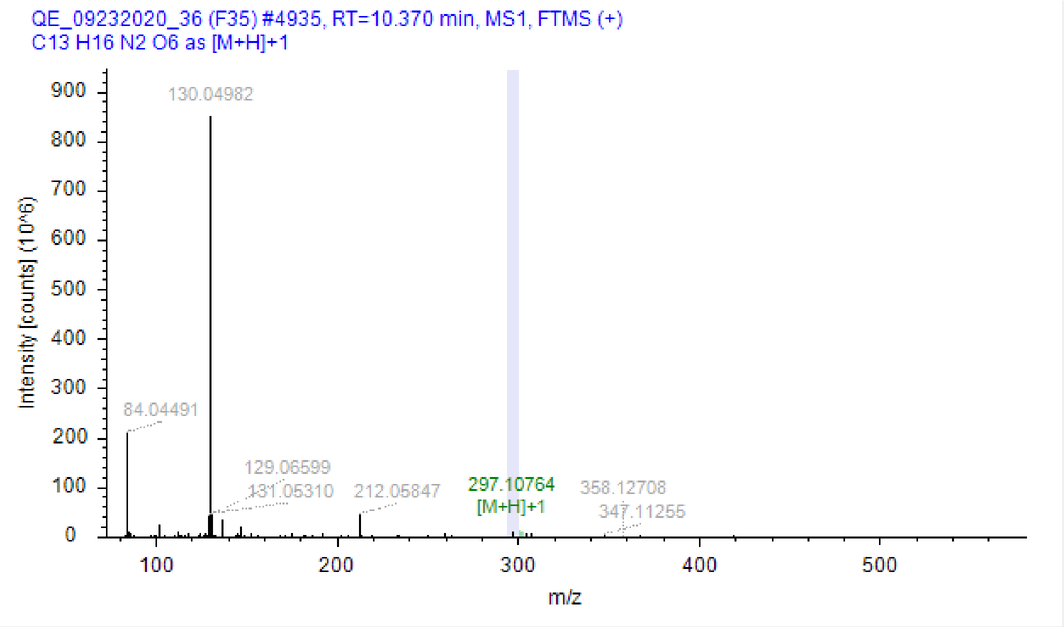

(b) MS2

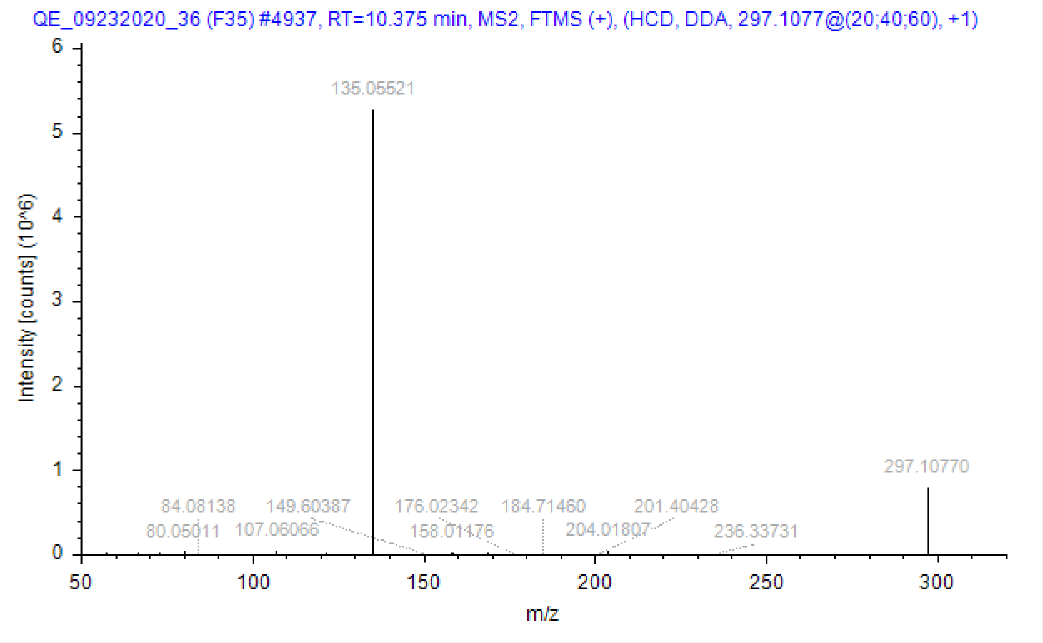

Figure S17: Mass Spectra (a: MS1 b: MS2) and proposed structures for unknown M297

(Link to Summary Table)

118.05327 Accurate Mass: Benzimidazole (parent compound)

(a) MS1

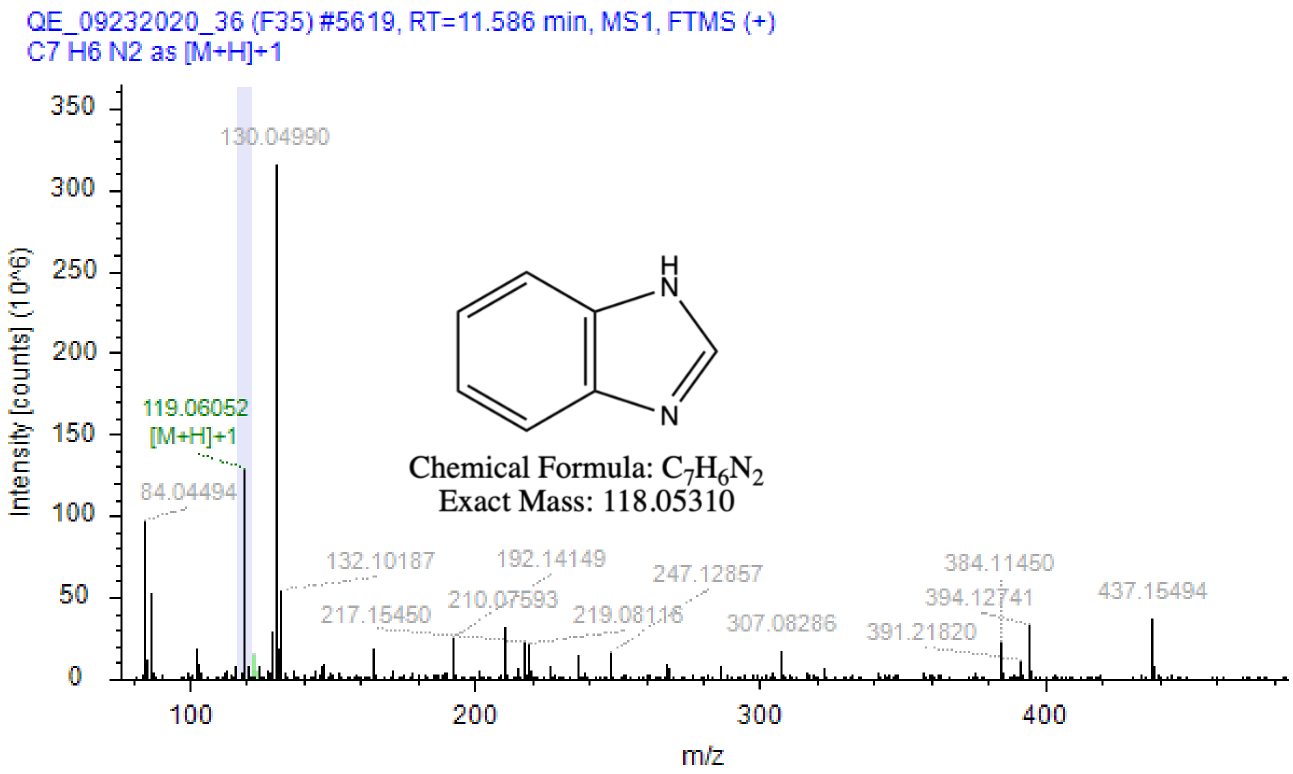

(b) MS2

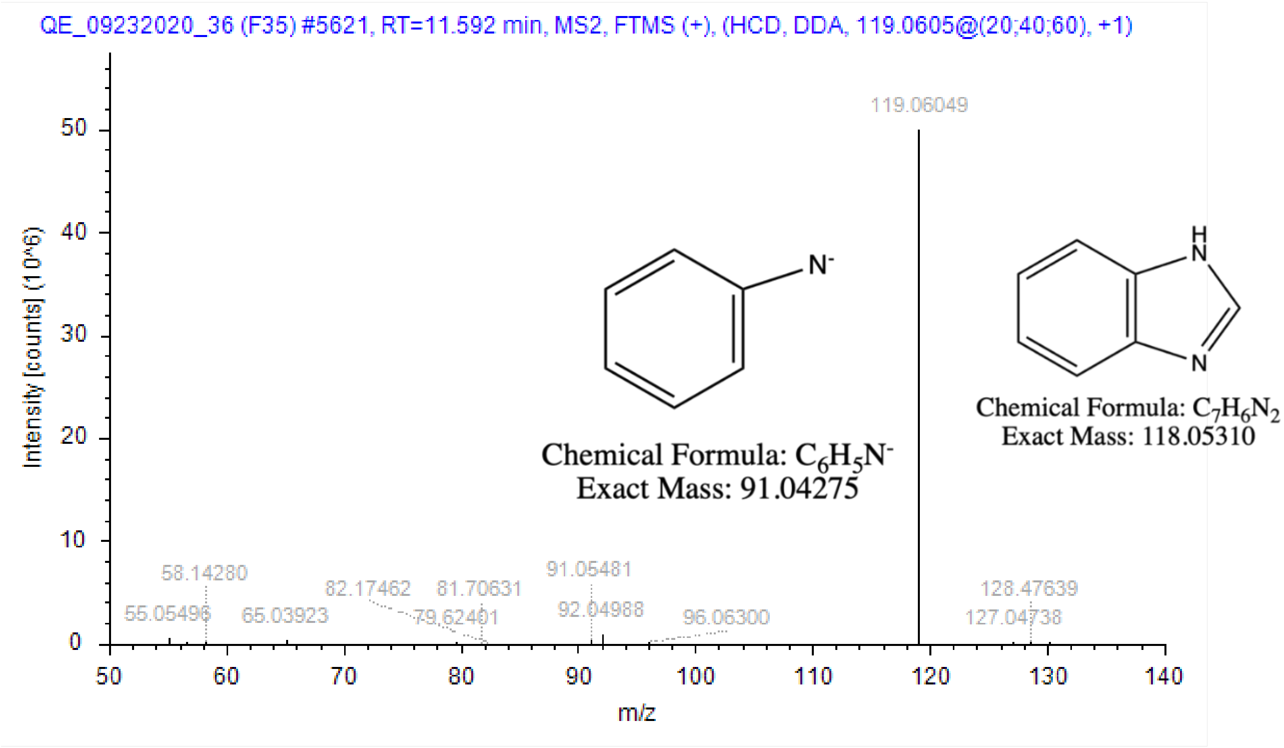

Figure S18: Mass Spectra (a: MS1 b: MS2) and structures for benzimidazole (parent compound)

Standard addition:

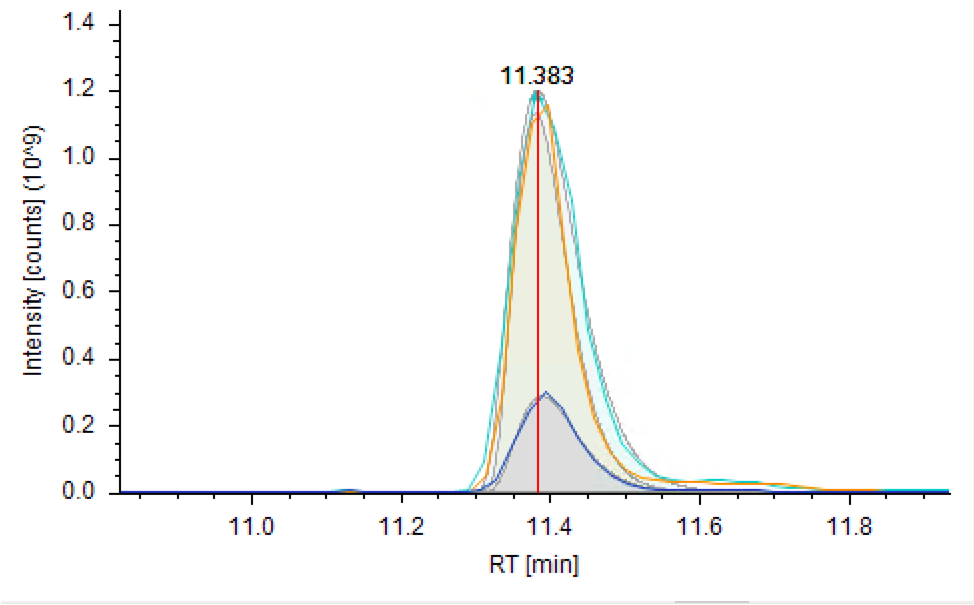

Figure S19: Results of standard addition for benzimidazole

The dark blue lower peak is the original composite BZ plant extract sample. The orange peak is the BZ standard in 50% methanol-50% DI water, run alone. The light blue peak is the BZ-spiked composite plant extract sample

(a) Standard addition MS spectra:

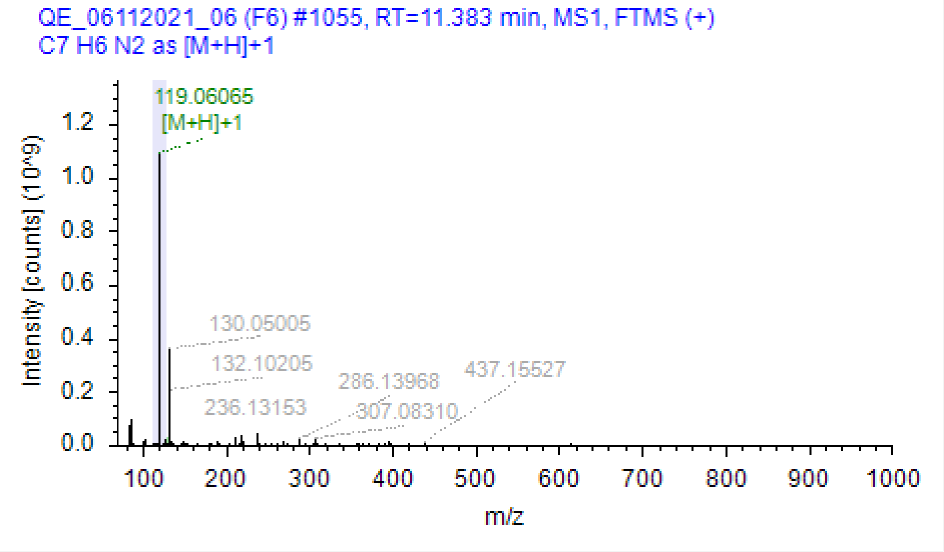

(b) Standard MS spectra:

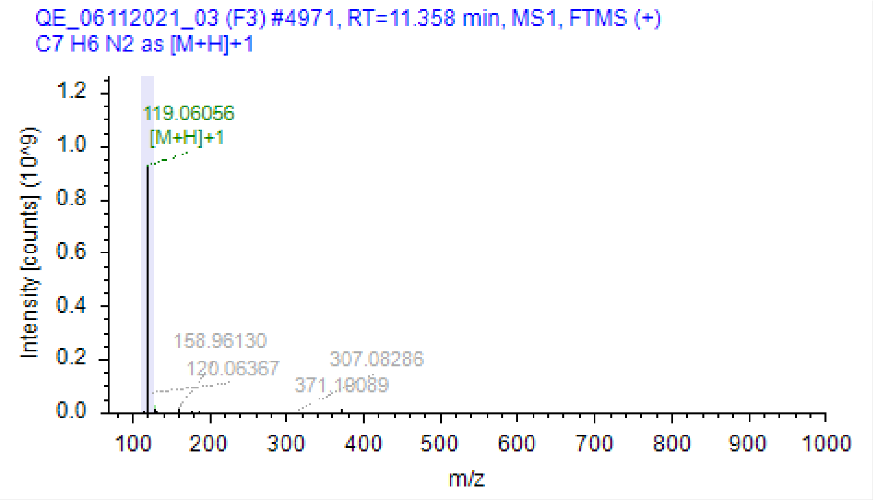

Figure S20: Mass spectra of standard addition for benzimidazole (a: Standard addition MS spectra b: Standard MS spectra)

(Link to Summary Table)

CN-BZ-Only Metabolites

<u>Unknown M179</u>

(a) MS1

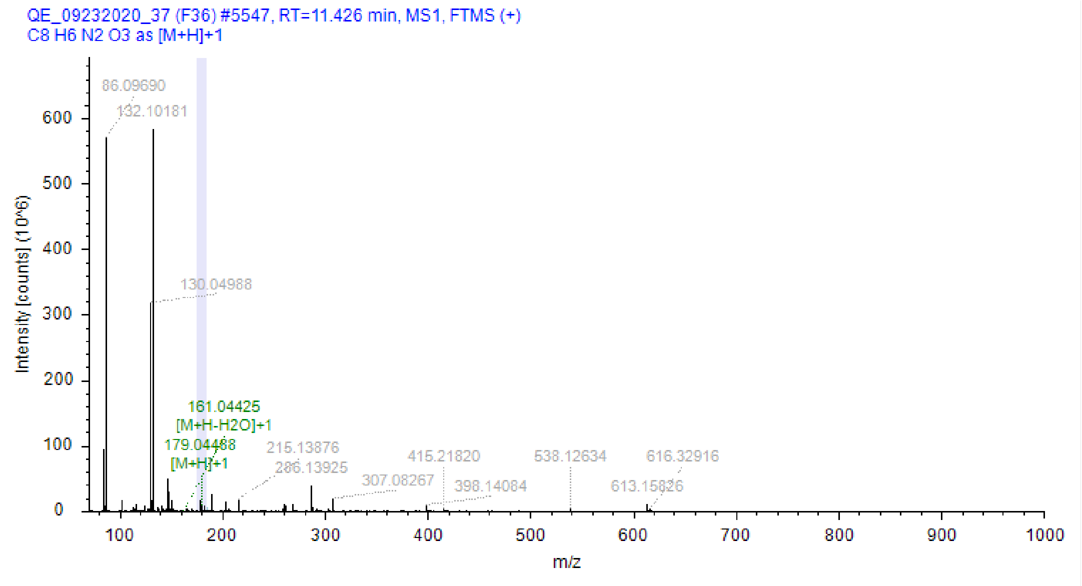

(b) MS1, additional peak in negative mode:

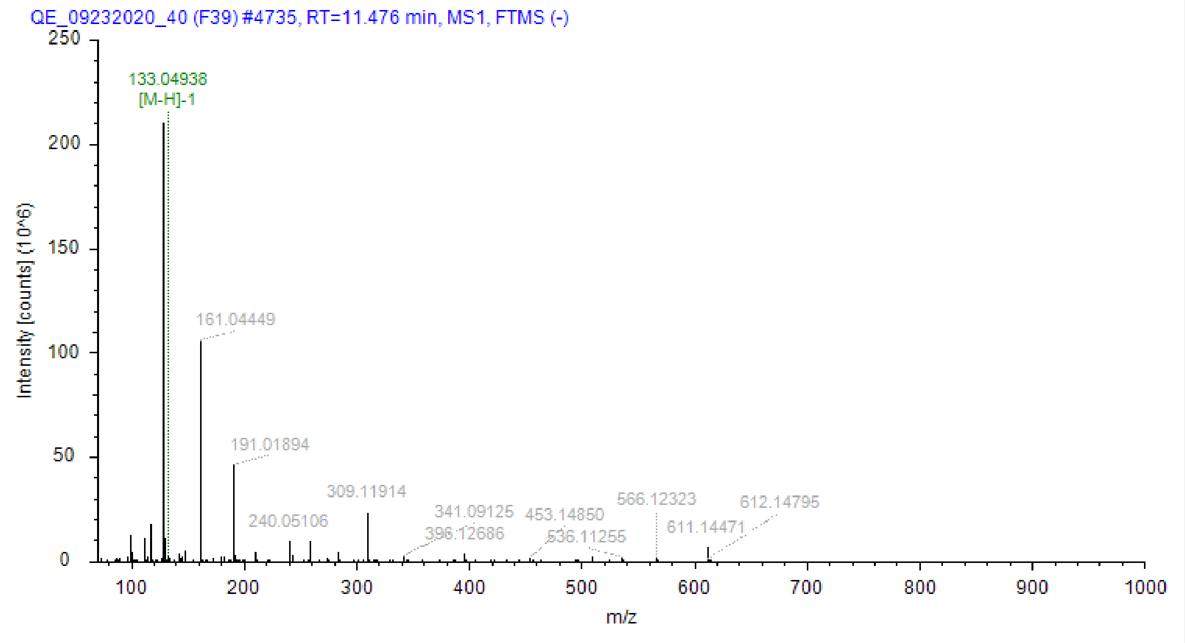

(c) MS2

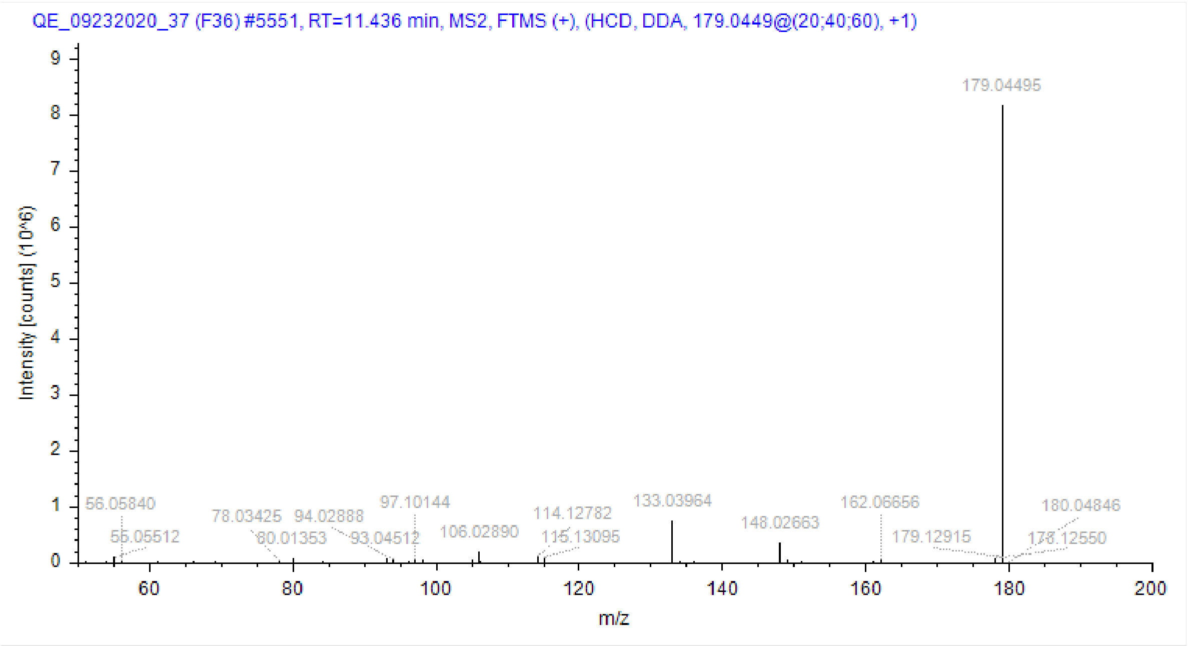

Figure S21: Mass spectra of unknown M179 (a: MS1 b: MS1, additional peak in negative mode c: MS2)

(Link to Summary Table)

Cyano-hydrolyzed CN-BZ

(a) MS1

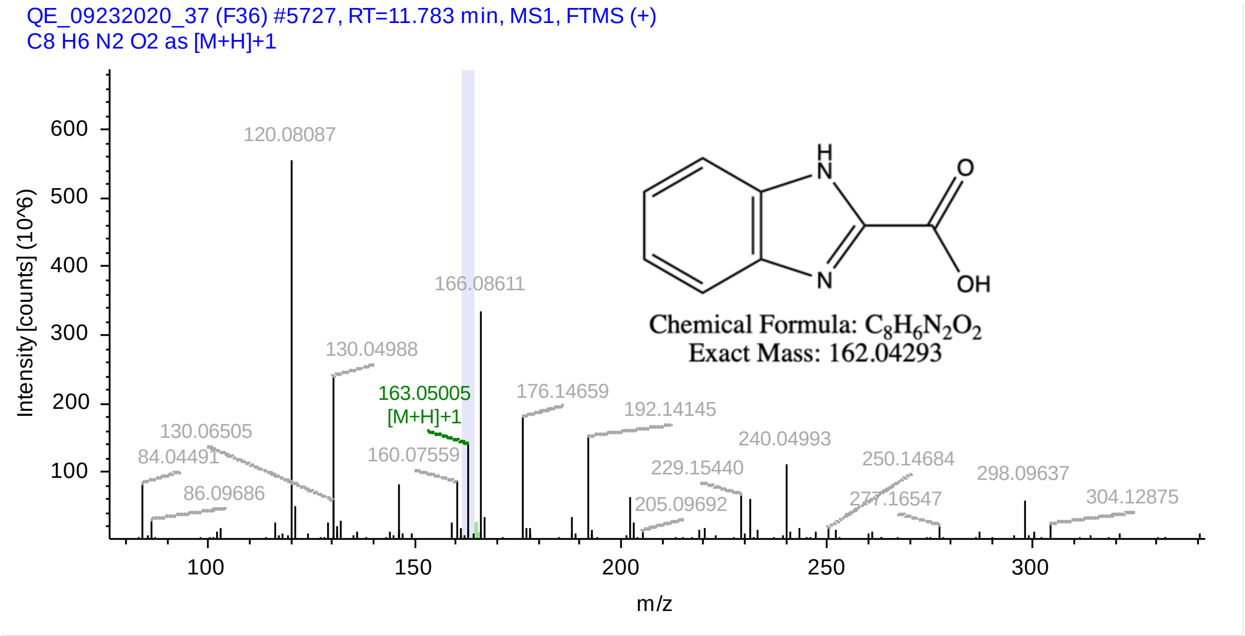

(b) MS2

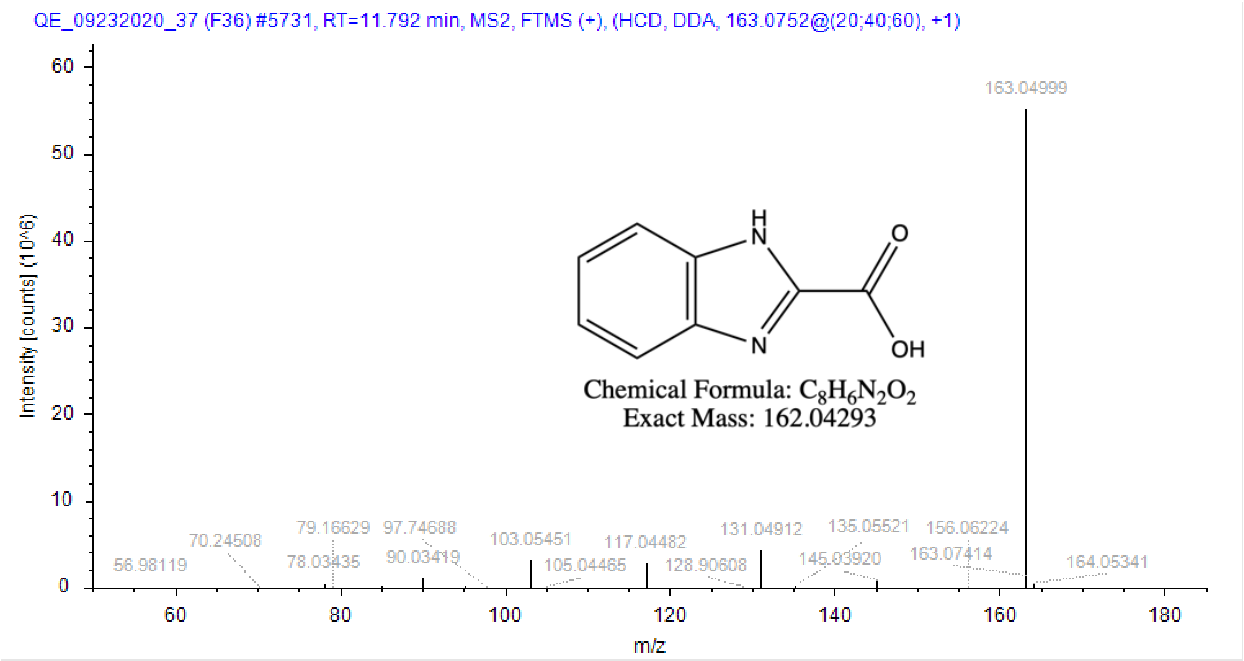

Figure S22: Mass spectra of cyano-hydrolyzed CN-BZ (a: MS1 b: MS2)

(Link to Summary Table)

CN-BZ plus N-acetylcysteine

(a) MS1

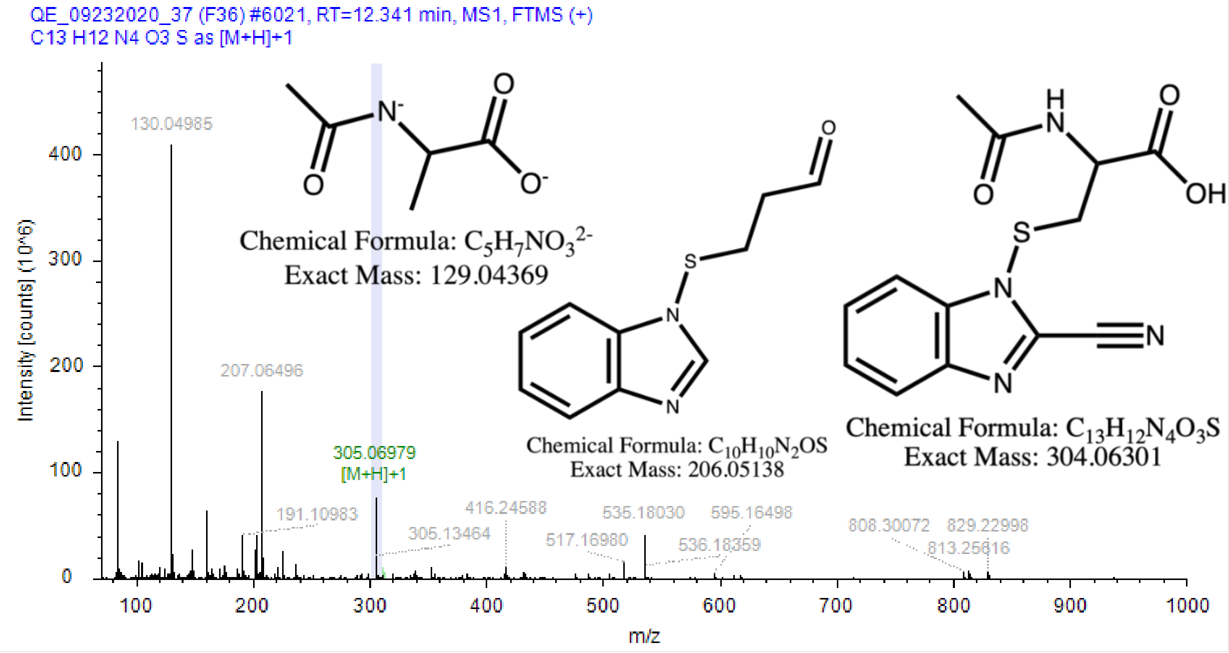

(b) MS2

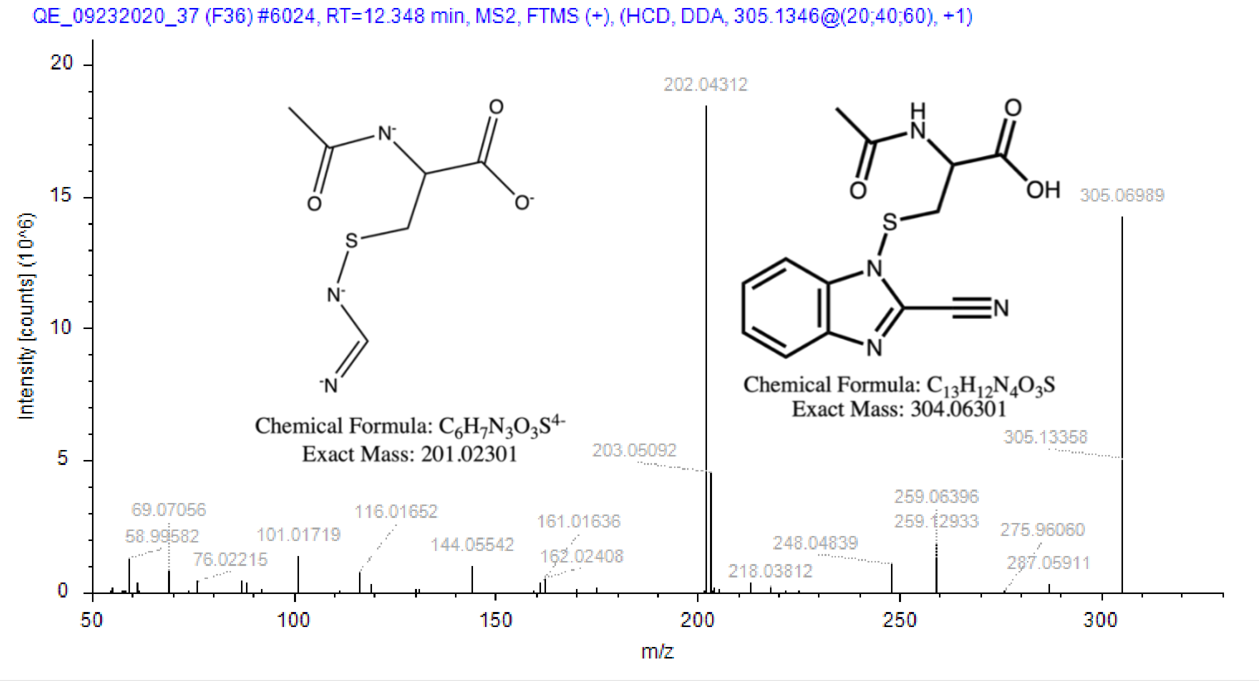

Figure S23: Mass spectra of cyano-hydrolyzed CN-BZ plus N-acetylcysteine (a: MS1 b: MS2)

(Link to Summary Table)

234.08989 Accurate Mass: BZ acetyl alanine

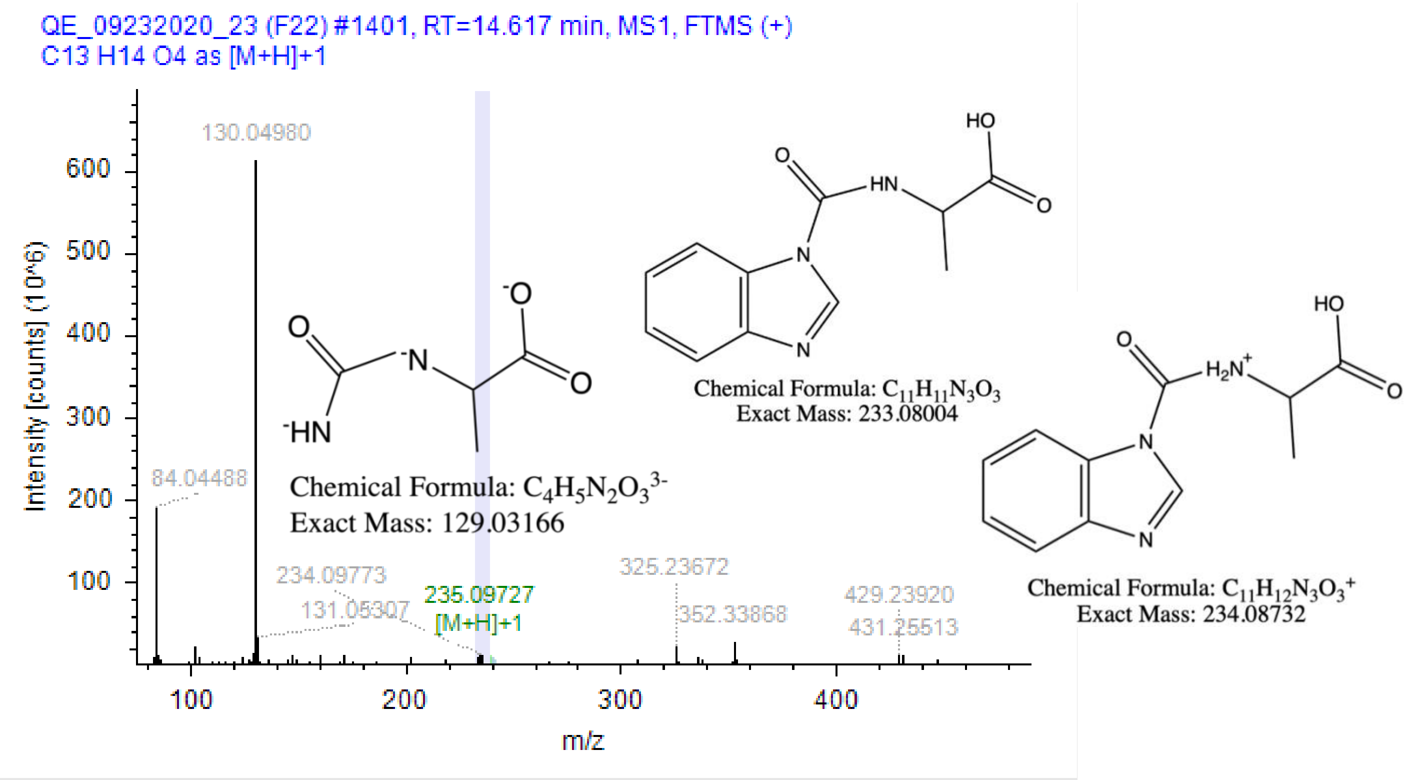

(No MS2 available)

Figure S24: Mass spectrum (MS1) of BZ acetyl alanine

(Link to Summary Table)

CDM-Only Metabolites

Unknown M780

(a) MS1

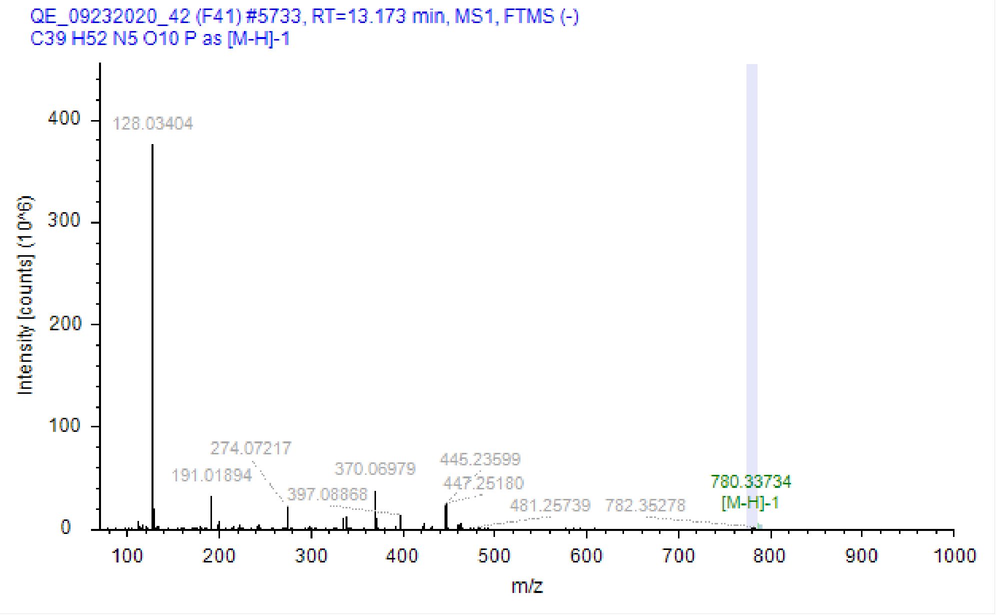

(b) MS2

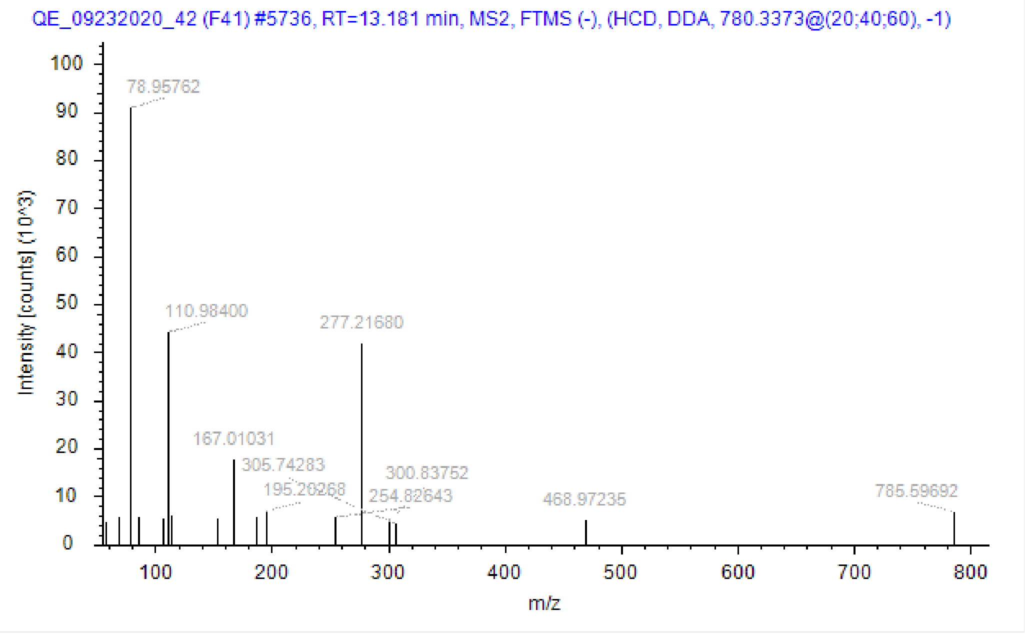

Figure S25: Mass spectra of unknown M780 (a: MS1 b: MS2)

(Link to Summary Table)

References

(1) LeFevre, G. H.; Müller, C. E.; Li, R. J.; Luthy, R. G.; Sattely, E. S. Rapid Phytotransformation of Benzotriazole Generates Synthetic Tryptophan and Auxin Analogs in Arabidopsis. *Environ. Sci. Technol.* **2015**, *49* (18), 10959–10968.

(2) Schooley, J. B.-. A Comparison of the Modes of Action of Three Benzimidazoles. *Phytopathology* **1971**, *61* (7), 816. https://doi.org/10.1094/phyto-61-816.

(3) Heneberg, P.; Svoboda, J.; Pech, P. Benzimidazole Fungicides Are Detrimental to Common Farmland Ants. *Biol. Conserv.* **2018**, *221*, 114–117. https://doi.org/10.1016/j.biocon.2018.03.004.

(4) Keri, R. S.; Hiremathad, A.; Budagumpi, S.; Nagaraja, B. M. Comprehensive Review in Current Developments of Benzimidazole-Based Medicinal Chemistry. *Chem. Biol. Drug Des.* **2015**, *86* (1), 799–845. https://doi.org/10.1111/cbdd.12462.

(5) Salahuddin; Shaharyar, M.; Mazumder, A. Benzimidazoles: A Biologically Active Compounds. *Arabian Journal of Chemistry*. Elsevier B.V. February 1, 2017, pp S157–S173. https://doi.org/10.1016/j.arabjc.2012.07.017.

(6) Huntscha, S.; Hofstetter, T. B.; Schymanski, E. L.; Spahr, S.; Hollender, J. Biotransformation of Benzotriazoles: Insights from Transformation Product Identification and Compound-Specific Isotope Analysis. *Environ. Sci. Technol.* **2014**, *48* (8), 4435–4443. https://doi.org/10.1021/es405694z.

(7) Zubrod, J. P.; Bundschuh, M.; Arts, G.; Brühl, C. A.; Imfeld, G.; Knäbel, A.; Payraudeau, S.; Rasmussen, J. J.; Rohr, J.; Scharmüller, A.; Smalling, K.; Stehle, S.; Schulz, R.; Schäfer, R. B. Fungicides: An Overlooked Pesticide Class? *Environ. Sci. Technol.* **2019**, *53* (7), 3347–3365. https://doi.org/10.1021/acs.est.8b04392.

(8) Hansch, C.; Leo, A.; Hoekman, D. *Exploring QSAR - Hydrophobic, Electronic, and Steric Constants*; American Chemical Society: Washington DC, 1995.

(9) National Center for Biotechnology Information. *PubChem Compound Summary for CID 239699, 1H-Benzimidazol-1-amine*. https://pubchem.ncbi.nlm.nih.gov/compound/239699 (accessed 2021-05-21).

(10) National Center for Biotechnology Information. *PubChem Compound Summary for CID 843858, 1H-Benzimidazole-2-carbonitrile*. https://pubchem.ncbi.nlm.nih.gov/compound/843858#section=Chemical-and-Physical-Properties (accessed 2021-05-21).

(11) Information, N. C. for B. *PubChem Compound Summary for CID 78572, 2-Chlorobenzimidazole*. https://pubchem.ncbi.nlm.nih.gov/compound/78572#section=Chemical-and-Physical-Properties (accessed 2023-02-09).

(12) National Center for Biotechnology Information. *PubChem Compound Summary for CID 97234, 2-Nitrobenzimidazole*. https://pubchem.ncbi.nlm.nih.gov/compound/97234#section=Chemical-and-Physical-Properties (accessed 2021-05-21).

(13) National Center for Biotechnology Information. *PubChem Compound Summary for CID 19017767, 4-Chloro-1H-benzo[d]imidazol-2-amine*. https://pubchem.ncbi.nlm.nih.gov/compound/19017767#section=Chemical-and-Physical-Properties (accessed 2023-02-09).

(14) Sicbaldi, F.; Sacchi, G. A.; Trevisan, M.; Del Re, A. A. M. Root Uptake and Xylem Translocation of Pesticides from Different Chemical Classes. *Pestic. Sci.* **1997**, *50* (2),

111–119. https://doi.org/10.1002/(SICI)1096-9063(199706)50:2<111::AID-PS573>3.0.CO;2-3.

(15) National Center for Biotechnology Information. *PubChem Compound Summary for CID 5430, Thiabendazole*. https://pubchem.ncbi.nlm.nih.gov/compound/5430#section=LogP (accessed 2021-05-21).

(16) National Center for Biotechnology Information. *PubChem Compound Summary for CID 1367, 1-Aminobenzotriazole*. https://pubchem.ncbi.nlm.nih.gov/compound/1367#section=Chemical-and-Physical-Properties (accessed 2021-05-21).

(17) LeFevre, G. H.; Portmann, A. C.; Müller, C. E.; Sattely, E. S.; Luthy, R. G. Plant Assimilation Kinetics and Metabolism of 2-Mercaptobenzothiazole Tire Rubber Vulcanizers by Arabidopsis. *Environ. Sci. Technol.* **2016**, *50* (13), 6762–6771.

(18) Muerdter, C. P.; Powers, M. M.; Chowdhury, S.; Mianecki, A. L.; LeFevre, G. H. Rapid Plant Uptake of Isothiazolinone Biocides and Formation of Metabolites by Hydroponic Arabidopsis. *Environ. Sci. Process. Impacts* **2022**, *24* (10), 1735–1747.

(19) Macherius, A.; Eggen, T.; Lorenz, W.; Moeder, M.; Ondruschka, J.; Reemtsma, T. Metabolization of the Bacteriostatic Agent Triclosan in Edible Plants and Its Consequences for Plant Uptake Assessment. *Environ. Sci. Technol.* **2012**, *46* (19), 10797–10804. https://doi.org/10.1021/es3028378.

(20) Purwaha, P.; Silva, L. P.; Hawke, D. H.; Weinstein, J. N.; Lorenzi, P. L. An Artifact in LC-MS/MS Measurement of Glutamine and Glutamic Acid: In-Source Cyclization to Pyroglutamic Acid. *Anal. Chem.* **2014**, *86* (12), 5633–5637. https://doi.org/10.1021/ac501451v.
